# Macrophage pyroptosis drives luminal senescence and club-cell reprogramming in 5α-reductase inhibitor-treated prostatic hyperplasia

**DOI:** 10.1038/s12276-026-01864-3

**Published:** 2026-10-07

**Authors:** Zheng Li, Zhenan Zhang, Qiancheng Zhao, Zhenpeng Zhu, Kaiwei Yang, Yisen Meng, Qian Zhang, Wei Yu

**Affiliations:** 1 Department of Urology, Peking University First Hospital, Institute of Urology, Beijing Key Laboratory of Urogenital Diseases (Male), Molecular Diagnosis and Treatment Center., National Research Center for Genitourinary Oncology, Peking University, Beijing, China; 2 Department of Urology, The Second Affiliated Hospital, School of Medicine, Zhejiang University, Hangzhou, China; 3 Department of Urology, Beijing Shijitan Hospital, Capital Medical University, Beijing, China

**Keywords:** Senescence, Cell death and immune response, Checkpoint signalling, Prostatic diseases, Prognostic markers

## Abstract

Benign prostatic hyperplasia (BPH) is a common age-associated disease whose prevalence rises sharply with advancing age. Therapeutic responses to 5α-reductase inhibitors (5-ARIs) in BPH are heterogeneous and may ultimately culminate in the need for surgery; however, the mechanisms that permit disease progression during treatment remain unclear. Here we integrate spatial and single-cell transcriptomics with functional perturbations and mouse genetics to delineate a macrophage-orchestrated pyroptosis–senescence–club axis in the human prostate. In 5-ARI-exposed tissue, we observe enrichment of cleaved-GSDMD⁺ macrophages, elevated epithelial p16^INK4A^ expression and expansion of SCGB1A1⁺ club cells. Spatial mapping pinpoints macrophage–epithelium hotspots with prominent MIF–CD74 and HMGB1–AGER signaling. Under 5-ARI pressure, luminal-cell secretomes recruit macrophages via MIF–CD74, and pyroptotic macrophages release HMGB1 that activates epithelial AGER to trigger luminal G1 arrest and senescence, which in turn promotes luminal-to-club reprogramming through a partially Notch-associated program. Blockade of MIF–CD74 or HMGB1–AGER attenuates macrophage pyroptosis and luminal senescence, whereas Notch inhibition partially reverses epithelial remodeling. A 13-gene pyroptosis–macrophage signature (PM-13) stratifies patient responsiveness to 5-ARIs. In a castration/testosterone-induced mouse BPH model, 5-ARI treatment amplifies pyroptotic macrophages and club programs, whereas Gsdmd deletion blunts pyroptosis and dampens senescence/club transcriptional states. These findings nominate druggable nodes—MIF–CD74, HMGB1–AGER, Notch and GSDMD—as candidate targets for therapeutic intervention and patient stratification in BPH under 5-ARI therapy.

Keywords: spatial and single-cell transcriptomics; benign prostatic hyperplasia; 5-alpha-reductase inhibitors; cellular senescence; macrophage pyroptosis; luminal-to-club conversion; MIF–CD74/HMGB1–AGER signaling; GSDMD.

**Figure:**
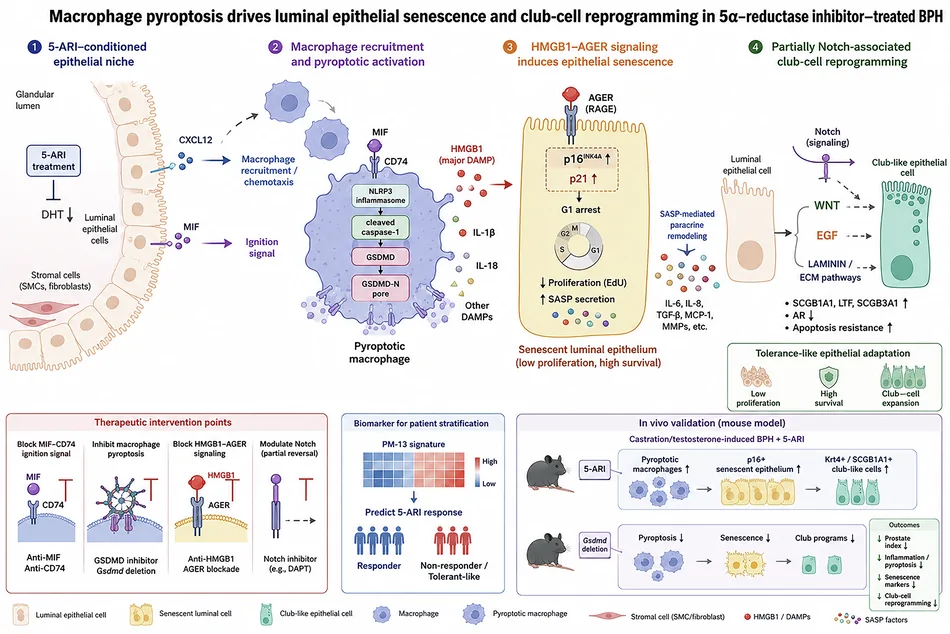

## Targeting MIF–CD74 and HMGB1–AGER in BPH therapy

Benign prostatic hyperplasia (BPH) is a prevalent condition in aging men, often leading to lower urinary tract symptoms and significant healthcare costs. This study explores the heterogeneous responses to 5α-reductase inhibitors (5-ARIs), commonly used to manage BPH. The authors propose that, under 5-ARI treatment, epithelial cells recruit macrophages, triggering a cascade of pyroptosis and inflammation, mediated by the MIF–CD74 and HMGB1–AGER axes. Using spatial transcriptomics, single-cell RNA sequencing and in vitro experiments, they demonstrate that 5-ARI exposure leads to epithelial lineage remodeling and senescence, with macrophages playing a central role. The study finds that macrophage pyroptosis contributes to epithelial senescence and lineage drift, suggesting a tolerance-like adaptation in BPH tissues. These findings highlight potential therapeutic targets for improving BPH treatment outcomes and suggest future research directions in targeting these pathways to enhance 5-ARI efficacy.

This summary was initially drafted using artificial intelligence, then revised and fact-checked by the author.

## Introduction

Benign prostatic hyperplasia (BPH) is common in aging men: histologic disease is present in ~50% by the sixth decade and in most thereafter, and BPH-attributable lower urinary tract symptoms (LUTS) affect many older adults^1–4^. The global burden keeps rising—~94 million men in 2019, over twice 1990—with the steepest growth in lower-sociodemographic regions while absolute numbers remain high in high-income settings^5,6^. Given links to nocturia, weak stream, urgency, infection, retention and downstream renal/bladder complications, BPH imposes substantial quality-of-life and healthcare costs^7^.

5α-reductase inhibitors (5-ARIs) reduce dihydrotestosterone, slow prostatic growth and lower risks of acute retention and surgery, and are widely used first-line therapies^3,8–10^. Yet, responses are heterogeneous: a sizable subset progresses despite treatment, and conventional stratifiers—prostate volume, prostate-specific antigen, symptom scores and related baseline clinical variables—capture part of progression risk but have limited patient-level predictive power^11,12^, suggesting that microenvironmental cues and cell-state programs also shape sensitivity.

Multi-omics increasingly depict BPH as a chronic ecosystem—coupling low-grade inflammation, epithelial/stromal senescence and lineage plasticity—rather than simple enlargement^13–15^. Although senescent cells exit the cell cycle, their senescence-associated secretory phenotype (SASP; for example, IL-1/IL-6 and TGF-β/BMP/NOTCH) can elevate apoptotic thresholds, rewire fate decisions and accelerate remodeling^16–18^. Across chronic pathologies, paracrine SASP and damage-associated molecular patterns (DAMPs) activate NF-κB/STAT3/MAPK survival circuitry and bias lineage choices; conversely, targeting senescence can restore drug sensitivity in part^19,20^. Whether these principles apply to human BPH under 5-ARI pressure remains unresolved.

Macrophages, central to immune surveillance and repair in aging tissues, are commonly increased in BPH^21–23^. Pyroptosis—an inflammasome–caspase-1–GSDMD-dependent death—releases IL-1β/IL-18 and DAMPs such as HMGB1, which can impose DNA-damage responses and senescence on neighboring cells^24–26^. In 5-ARI-treated BPH, it is unknown whether an in situ ‘macrophage recruitment → pyroptosis → DAMP cascade’ exists, and whether DAMP–receptor signaling enforces epithelial senescence and redirects lineage trajectories.

Single-cell RNA sequencing (scRNA-seq) and spatial transcriptomics (ST) provide complementary views. scRNA-seq resolves senescence states (p16/p21, DNA-damage responses, SASP), survival/apoptosis programs and developmental signaling at single-cell resolution and supports trajectory inference. ST maps these states onto tissue coordinates to define neighborhood architecture and ligand–receptor hotspots—who signals to whom, and where—thereby reducing bulk averaging and dissociation bias.

We hypothesized that, under 5-ARI exposure, luminal epithelial cues recruit macrophages and trigger pyroptosis, and that released DAMPs (exemplified by HMGB1) signal via epithelial AGER to sustain senescence, raise apoptotic thresholds and promote club-like fate drift. To test this, we integrated 14 human ST sections, used published human prostate scRNA-seq references for cell-type annotation, and reanalyzed public mouse prostate scRNA-seq data to define cellular composition, spatial trajectories and signaling programs, complemented by immunohistochemistry (IHC)/digital pathology and mechanistic in vitro perturbations (conditioned media (CM)/Transwell, pyroptosis blockade with orthogonal readouts, co-immunoprecipitation (co-IP) and genetic/pharmacologic tests). In an independent cohort, we derived and validated a 13-gene ‘Pyroptosis ⊕ Macrophage’ (PM-13) signature for 5-ARI response stratification, and we performed causal tests using Gsdmd deletion in a castration/testosterone-induced mouse BPH model exposed to 5-ARI treatment. Collectively, these data define a macrophage-sculpted luminal senescent niche in BPH, provide a mechanistic framework for 5-ARI-associated remodeling and tolerance-like adaptation, and nominate actionable nodes (MIF/CD74, HMGB1/AGER, Notch and GSDMD) to support stratified combination strategies.

## Materials and methods

### Patient samples and tissue collection

Paraffin-embedded prostate tissues were obtained from patients with BPH undergoing TURP for lower urinary tract symptoms. Exclusion criteria included prostate cancer history/current diagnosis and confounding histopathologic findings. All procedures complied with the Declaration of Helsinki and were approved by the institutional ethics committee; written informed consent was obtained. In total, 50 patients were included, comprising 25 patients with untreated BPH and 25 independent patients with BPH after 5-ARI treatment. The two groups were clinically comparable at the cohort level for major baseline variables, including age, body mass index and prostate volume. Serial adjacent sections from each tissue block were used for matched marker staining within the same patient sample.

### ST data processing and analysis (concise)

Public GEO ST datasets GSE179615 and GSE278936 (14 sections: normal *n* = 2; BPH without medication *n* = 6; BPH after 5-ARI* n* = 6) were integrated in R using Seurat/SCTransform. Tissue-covered spots were retained after excluding spots with fewer than 200 detected genes or more than 20% mitochondrial reads; genes detected in fewer than five spots were removed before downstream analysis. Data were normalized with SCTransform (~3,000 HVGs) and integrated using RPCA anchor integration. PCA/UMAP embedding and Leiden clustering were performed, yielding 48 clusters consolidated into 12 major cell classes by label transfer from published prostate scRNA-seq references with manual curation. Compositional analyses used nonparametric tests with Benjamini–Hochberg false discovery rate (FDR) control; epithelial subsets were analyzed by donor-level pseudobulk DE (DESeq2) and pathway enrichment (ORA/GSEA/GSVA). Pseudotime/lineage analyses used Monocle3 with Slingshot validation. Spatial neighborhood-aware ligand–receptor inference used CellChat and NicheNetR with permutation-based significance, highlighting epithelial–macrophage interactions (for example, MIF–CD74 and HMGB1–AGER). Detailed parameters/codes are presented in the Supplementary Detailed Methods.

To address potential mixed-spot artifacts in Visium-based ST, we further performed nearest-neighbor analysis in ST sections with available spatial coordinate files.

Spatial coordinates were obtained from the tissue_positions_list.csv file and matched to the integrated Seurat object using spatial spot barcodes and sample identifiers. Spot-level expression values were extracted from the normalized expression matrix. EPCAM, KRT8, KRT18 and KRT19 were used to calculate an epithelial module score to define epithelial-enriched spatial spots; CD68, LYZ, AIF1, CSF1R, C1QA, C1QB, C1QC, CD14 and FCGR3A were used to calculate a macrophage module score to define macrophage-enriched spatial spots. Within each section, epithelial-enriched spots were defined as those in the top quartile of epithelial module scores, whereas macrophage-enriched spots were defined as those in the top decile of macrophage module scores.

For the MIF–CD74/GSDMD axis, the targeted distance was calculated as the distance from MIF-high epithelial-enriched spots to the nearest CD74/GSDMD-high macrophage-enriched spots. The background distance was calculated as the distance from epithelial-enriched spots to the nearest macrophage-enriched spots. For the HMGB1/GSDMD–AGER axis, the targeted distance was calculated as the distance from HMGB1/GSDMD-high macrophage-enriched spots to the nearest AGER-high epithelial-enriched spots; the background distance was calculated as the distance from macrophage-enriched spots to the nearest epithelial-enriched spots. Ligand- or receptor-high spots were defined within each section based on the upper quartile of positive expression values. For combined macrophage states, CD74/GSDMD-high and HMGB1/GSDMD-high macrophage-enriched spots were defined using the average scaled expression of the indicated genes within macrophage-enriched spots, followed by upper-quartile thresholding within each section.

Euclidean nearest-neighbor distances were calculated using full-resolution pixel coordinates and converted to micrometers using the Visium spot diameter scaling factor. For statistical analysis, distances were summarized at the section level to avoid spot-level pseudoreplication. Across sections with available coordinates, targeted distances and background distances were compared in a paired manner. This analysis was interpreted as exploratory spatial evidence and integrated with results from multiplex immunofluorescence (IF) and functional perturbation experiments.

### IHC and image quantification (OD and semiquantitative scoring)

Formalin-fixed, paraffin-embedded (FFPE) sections (4 μm) underwent deparaffinization/rehydration, heat-induced antigen retrieval (citrate pH 6.0 or EDTA pH 9.0), peroxidase quenching and blocking; primary antibodies were incubated overnight and detected by HRP/DAB with hematoxylin counterstaining. IHC was performed on serial adjacent sections from all 50 patients, including 25 patients with untreated BPH and 25 independent patients with BPH after 5-ARI treatment. Consecutive sections from the same tissue block were used for matched marker staining within each sample. For digital-pathology quantification, five non-overlapping periglandular regions of interest (ROIs)/high-power fields (HPFs) were analyzed under identical imaging settings. Staining-intensity and percentage-positive-area measurements from the five fields were averaged to generate one patient-level value.

Semiquantitative IHC staining was assessed independently by two blinded pathologists using a four-level scale: 0, negative; 1, weak; 2, moderate; and 3, strong. Discrepant field-level scores were adjudicated by a third pathologist. For each patient, scores from five non-overlapping ROIs/HPFs were averaged to generate one patient-level mean semiquantitative IHC score. Field-level scores were retained in the Source Data file for transparency but were not treated as independent statistical observations.

The untreated BPH and post-5-ARI BPH groups each comprised 25 independent patients. Patient-level semiquantitative IHC scores were compared using two-sided Mann–Whitney *U* tests. Patient-level staining-intensity and percentage-positive-area measurements were compared using two-sided Welch’s unpaired *t*-tests. Exact *P* values and individual patient-level values are provided in the Source Data file.

### Multiplex IF quantification and spatial proximity analysis

Multiplex IF was performed on untreated BPH and 5-ARI-treated BPH tissue sections to assess epithelial–macrophage spatial organization at the protein level. A total of 50 patient samples were analyzed, including 25 untreated BPH samples and 25 5-ARI-treated BPH samples. For each tissue section, five non-overlapping gland-containing fields of view were selected and imaged under identical acquisition settings.

For the MIF–CD74 axis, KRT8^+^ luminal epithelial regions, MIF signal, CD68^+^ macrophages, CD74 signal and GSDMD-N signal were segmented from multiplex images. For the HMGB1–AGER axis, KRT8^+^ luminal epithelial regions, AGER signal, CD68^+^ macrophages, GSDMD-N signal and HMGB1 signal were segmented. Mean MIF and AGER fluorescence intensities were quantified within KRT8^+^ epithelial masks after background subtraction and normalized to the mean value of the untreated BPH group for visualization.

For macrophage-state quantification, CD68^+^ macrophages were used as the denominator. CD68^+^CD74^+^GSDMD-N^+^ macrophages and CD68^+^GSDMD-N^+^HMGB1^+^ macrophages were expressed as percentages of total CD68^+^ macrophages. For spatial proximity analysis, object centroids were exported from segmented multiplex IF images. The nearest-neighbor distance from each MIF^+^KRT8^+^ epithelial object to the closest CD68^+^CD74^+^GSDMD-N^+^ macrophage object was calculated for the MIF–CD74 axis. The nearest-neighbor distance from each CD68^+^GSDMD-N^+^HMGB1^+^ macrophage object to the closest KRT8^+^AGER^+^ epithelial object was calculated for the HMGB1–AGER axis. Pixel distances were converted to micrometers using calibrated image metadata.

For each patient sample, measurements from five fields of view were averaged to generate one patient-level value before statistical analysis. Each dot in the multiplex IF quantification panels (Supplementary Fig. 8b–h) represents one patient-level value. These panels included 25 untreated BPH patients and 25 independent 5-ARI-treated patients with BPH, and patient-level comparisons were performed using two-sided Welch’s unpaired *t*-tests. For the ST nearest-neighbor analyses in Supplementary Fig. 8i,j, distances were summarized at the section level and compared using two-sided paired* t*-tests, with *n* = 4 paired sections per comparison. Exact *P* values are provided in the Source Data file.

### PM-13 composite signature: construction and validation

A public cohort (GSE262946) with paired baseline/follow-up samples was analyzed in a nested cross-validation framework. Candidate genes were drawn from pyroptosis/macrophage-related pathways; features were selected by sequential forward selection with local backward steps, with outer folds stratified by patient ID. The PM-13 score was computed as the mean of per-gene *z* scores with orientation such that higher scores indicate resistance. Full implementation details are presented in the Supplementary Detailed Methods.

#### Cell culture, primary luminal epithelium and macrophages

BPH-1 cells were obtained commercially and used under mycoplasma-negative conditions. Primary luminal epithelial cells (PrLCs) were derived from fresh TURP specimens and used at early passages. Unless otherwise specified, epithelial cells were cultured in RPMI-1640 with 10% fetal bovine serum and penicillin–streptomycin. Human monocyte-derived macrophages (hMDMs) were generated from PBMC-derived CD14⁺ monocytes and differentiated with M-CSF (50 ng/ml) for 7 days. Detailed isolation/enrichment steps (for example, EpCAM⁺/CD26^high^ workflow) and media formulations are presented in the Supplementary Detailed Methods.

#### Finasteride treatment and CM

BPH-1/PrLCs were seeded at 2 × 10^5^ cells/well (6-well plates) and treated with finasteride (10 μM, 48 h) or vehicle. Supernatants were clarified and 0.22-μm filtered to generate CM (5-ARI–CM versus Ctrl-CM) and used fresh or stored at −80 °C. CM handling/normalization rules are presented in the Supplementary Detailed Methods.

### Post-isolation culture

PrLCs were plated in 10-cm dishes and allowed to adhere for 24 h in DMEM/F12 epithelial growth medium supplemented with EGF and bFGF (see formulation and vendors listed in Supplementary Table. Non-adherent cells were removed, fresh medium was added and cultures were expanded until epithelial colonies formed. Experiments used early passages (≤P5).

#### Chemotaxis and blockade/neutralization

Macrophage chemotaxis was assessed in 24-well Transwells (8-μm pores) with CM in the lower chamber and 5 × 10^4^ hMDMs in the upper chamber for 8 h. Blockade/neutralization included anti-MIF (2 μg/ml; CM preincubation), anti-CXCL12 (100 μg/ml; CM preincubation), CD74-blocking antibody (2 μg/ml; macrophage pretreatment) and CXCR4 blockade (5 μg/ml; macrophage pretreatment), including combined conditions. Detailed counting/whole-membrane analysis procedures are presented in the Supplementary Detailed Methods.

### Secreted factors, qRT–PCR and cytotoxicity

Secreted analytes in epithelial CM and macrophage supernatants were quantified by enzyme-linked immunosorbent (ELISA) using standard curves and 4PL fitting, with biological and technical replication. RNA was extracted and quantified by SYBR-based qRT–PCR with GAPDH normalization (2^−ΔΔCt^). Cytotoxicity was assessed by LDH release. Complete kit/primer lists and QC thresholds are presented in the Supplementary Detailed Methods.

### Macrophage functional-state marker analysis

To assess macrophage functional-state changes after exposure to epithelial CM, hMDMs were treated with Ctrl-CM or 5-ARI-CM derived from BPH-1 cells or primary PrLCs. CM were prepared as described above from epithelial cells treated with vehicle or finasteride (10 μM, 48 h), clarified by centrifugation, filtered through 0.22-μm filters and applied to hMDMs under matched conditions.

After CM exposure, total RNA was extracted from hMDMs for qRT–PCR analysis of selected M1-like inflammatory markers (TNF, CXCL10 and CD86) and M2-like remodeling/regulatory markers (MRC1, CD163, IL10 and CCL18). qRT–PCR was performed using SYBR Green chemistry with the following human primer pairs: TNF, forward 5′-CTCTTCTGCCTGCTGCACTTTG-3′ and reverse 5′-ATGGGCTACAGGCTTGTCACTC-3′; CXCL10, forward 5′-GGTGAGAAGAGATGTCTGAATCC-3′ and reverse 5′-GTCCATCCTTGGAAGCACTGCA-3′; CD86, forward 5′-CCATCAGCTTGTCTGTTTCATTCC-3′ and reverse 5′-GCTGTAATCCAAGGAATGTGGTC-3′; MRC1, forward 5′-CCATCGAGGAAGAGGTTCGG-3′ and reverse 5′-GGTGGGTTACTCCTTCTGCC-3′; CD163, forward 5′-CCAGAAGGAACTTGTAGCCACAG-3′ and reverse 5′-CAGGCACCAAGCGTTTTGAGCT-3′; IL10, forward 5′-TCTCCGAGATGCCTTCAGCAGA-3′ and reverse 5′-TCAGACAAGGCTTGGCAACCCA-3′; and CCL18, forward 5′-GTTGACTATTCTGAAACCAGCCC-3′ and reverse 5′-GTCGCTGATGTATTTCTGGACCC-3′. GAPDH was used as the internal control. Relative expression was calculated using the 2^−ΔΔCt^ method, and values were normalized to the matched Ctrl-CM group, which was set to 1 for visualization.

Culture supernatants from the same experimental conditions were collected for ELISA-based quantification of inflammatory cytokines/chemokines and remodeling/regulatory factors using R&D Systems/Bio-Techne DuoSet ELISA kits: human TNF (DY210), IL-6 (DY206), CXCL10/IP-10 (DY266), IL10 (DY217B), TGF-β1 (DY240) and CCL18/PARC (DY394). Cytokine concentrations were calculated from standard curves generated for each ELISA plate. For graphical presentation, values were normalized to the matched Ctrl-CM group, which was set to 1, and are shown as relative cytokine concentrations.

All assays were performed using three independent biological replicates. For each qRT–PCR or ELISA readout, normalized values in the 5-ARI-CM group were compared with the theoretical control value of 1 using a two-sided one-sample* t*-test. Exact* P* values are provided in the Source Data file.

#### Pyroptosis and cell-death phenotyping

Macrophage membrane permeability and caspase-1 activation were assessed by Hoechst/propidium iodide (PI) staining plus FLICA imaging under identical exposure conditions, quantified across random fields. Caspase-1 activation was also measured by flow cytometry using FAM-FLICA (YVAD-FMK) with PI counterstaining and standardized compensation/gating; pyroptosis was defined as FAM⁺PI⁺ events. Detailed gating and instrument settings are provided in the Supplementary Detailed Methods.

#### Senescence and proliferation assays

Senescence was quantified by SA-β-gal staining (pH 6.0) after exposure to control/Ctrl+Mac/Pyro-CM ± anti-HMGB1, with blinded image-based quantification. Cell-cycle distribution was assessed by ethanol fixation and PI/RNase staining with doublet discrimination and modeling in FlowJo. DNA synthesis was evaluated by EdU incorporation during the last 2 h of treatment, followed by fluorescence imaging and EdU⁺ fraction quantification. Full protocols and analysis settings are presented in the Supplementary Detailed Methods.

### Recombinant HMGB1 stimulation and AGER knockdown

For ligand-sufficiency and receptor-dependency experiments, BPH-1 cells and primary PrLCs were transfected with negative-control siRNA (siNC) or AGER-targeting siRNA (siAGER) using the same siRNA transfection protocol applied in the AGER-perturbation experiments. After transfection, cells were treated with vehicle or recombinant human HMGB1 (rhHMGB1; BioLegend, cat. no. 557804; 3 μg/ml, 48 h). Vehicle-treated wells received an equivalent volume of the recombinant protein formulation buffer.

Senescence was assessed by SA-β-gal staining, cell-cycle distribution was analyzed by PI/RNase flow cytometry after ethanol fixation, and DNA synthesis was measured by EdU incorporation during the final 2 h of treatment. Secreted IL-1α, IL-6, IL-8, TNF and MCP-1 levels in culture supernatants were quantified by ELISA and normalized to the siNC+vehicle group. All assays were performed using three independent biological replicates per group. For each quantitative readout, group differences were analyzed using ordinary one-way analysis of variance (ANOVA) followed by Tukey’s multiple-comparisons test. For the cytokine measurements in panels m and n, each cytokine was analyzed separately. Exact *P* values are provided in the Source Data file.

#### HMGB1 receptor screening and co-IP validation

Candidate HMGB1 interactors were screened across BioGRID/IntAct/STRING with experimentally supported physical PPIs, yielding a focused candidate set (including AGER, TP53 and BECN1). Co-IP was performed in PrLCs exposed to control CM versus Pyro-CM, using anti-HMGB1 immunoprecipitation with IgG controls, followed by immunoblot validation. Detailed buffers/wash conditions and antibody specifics are presented in the Supplementary Detailed Methods.

### Mouse castration/testosterone-induced BPH model and 5-ARI treatment

To establish a reproducible testosterone-driven BPH model, endogenous androgen production was minimized by surgical castration, followed by exogenous testosterone administration to induce prostatic hyperplasia. This castration/testosterone protocol was used to standardize the androgenic background and reduce interanimal variability caused by endogenous testosterone fluctuations. Mice assigned to the 5-ARI arm received 5-ARI treatment in this standardized BPH model background. Thus, castration was part of the BPH model-establishment procedure and was not intended to model clinical androgen-deprivation therapy. The in vivo comparison was interpreted as 5-ARI exposure in a standardized castration/testosterone-induced BPH model. This modeling strategy is consistent with prior testosterone-driven BPH models using castration followed by testosterone replacement or testosterone propionate administration, including studies incorporating finasteride as a pharmacological comparator or standard 5-ARI intervention^27–30^.

#### Mouse prostate flow cytometry and multiplex IF

For mouse prostate immune profiling, dissociated single-cell suspensions were stained with viability dye and macrophage markers (CD45, F4/80 and CD64) followed by intracellular caspase-1 p20 staining; pyroptotic macrophages were quantified as caspase-1 p20⁺ within live CD45⁺F4/80⁺CD64⁺ cells. Multiplex IF was performed on FFPE sections with standardized imaging/ROI selection and per-animal averaging. Complete antibody panels, compensation controls and image-analysis pipelines are presented in the Supplementary Detailed Methods.

### Bulk RNA-seq and scRNA-seq (mouse)

Bulk RNA-seq reads were aligned to mouse GRCm39 (STAR two-pass) with gene-level counting (featureCounts), with alternative Salmon/tximport quantification as needed. Differential expression used DESeq2 (FDR <0.05; |log_2_fold change| ≥1) with pathway analyses by GSEA/GSVA. For scRNA-seq (GSE235514), Seurat was used with QC (200–6,000 genes per cell; ≤15% mitochondrial reads), SCTransform, SCT-anchor integration, UMAP embedding and Leiden clustering; major lineages were assigned by canonical markers with SingleR cross-checks. Full parameterization and code availability are presented in the Supplementary Detailed Methods.

### Statistics

Statistical tests were selected according to the experimental design and data distribution. Human untreated BPH and post-5-ARI BPH tissues represented two independent patient cohorts, with 25 independent patients per group. Measurements from multiple ROIs or fields of view within the same tissue sample were averaged to generate one patient-level value before statistical testing.

Patient-level semiquantitative IHC mean scores were compared using two-sided Mann–Whitney *U* tests. Patient-level digital-pathology staining-intensity and percentage-positive-area measurements were compared using two-sided Welch’s unpaired *t*-tests. Field-level observations were not treated as independent statistical units.

For ST nearest-neighbor analyses, distances were summarized at the section level to avoid spot-level pseudoreplication. Matched in vitro control and treatment conditions generated within the same biological replicate were analyzed using paired tests where appropriate. Multigroup experiments were analyzed using the panel-specific omnibus and multiplicity-adjusted post hoc tests reported in the figure legends and Supplementary Detailed Methods. All tests were two-sided. For experimentally generated quantitative panels, exact n definitions and statistical tests are provided in the figure legends and Supplementary Detailed Methods, whereas individual values and exact *P* values are provided in the Source Data file. Public-omics datasets and their accession numbers are described in the ‘Data availability’ section and Source Data file.

## Results

### ST reveals 5-ARI-induced epithelial lineage remodeling and senescence-associated niches in BPH prostates

We integrated 14 ST sections from GSE179615 and GSE278936, comprising normal (*n* = 2), untreated BPH (*n* = 6) and BPH after 5-ARI therapy (*n* = 6). After unified quality control and batch correction, 46,176 tissue-covered spots were retained. We applied an unsupervised workflow to the merged dataset (PCA → kNN/SNN → UMAP/Leiden) and assessed clustering stability across resolutions using clustree (Supplementary Fig. 1a,b). Based on continuity, marker interpretability and spatial coherence, we selected a resolution yielding 48 clusters (0–47; Supplementary Fig. 2a,b).

To annotate cell types, we transferred labels from published prostate scRNA-seq references and refined assignments using spatial context, resulting in 12 major classes (Supplementary Fig. 2c). UMAP, FeaturePlot, DotPlot and violin-plot visualization supported robust lineage separation and marker specificity across epithelial, stromal and immune compartments (Supplementary Fig. 2d,e and Supplementary Figs. 3 and 4). Full marker lists are provided in Supplementary Tables 1 and 2.

**Fig. 1:**
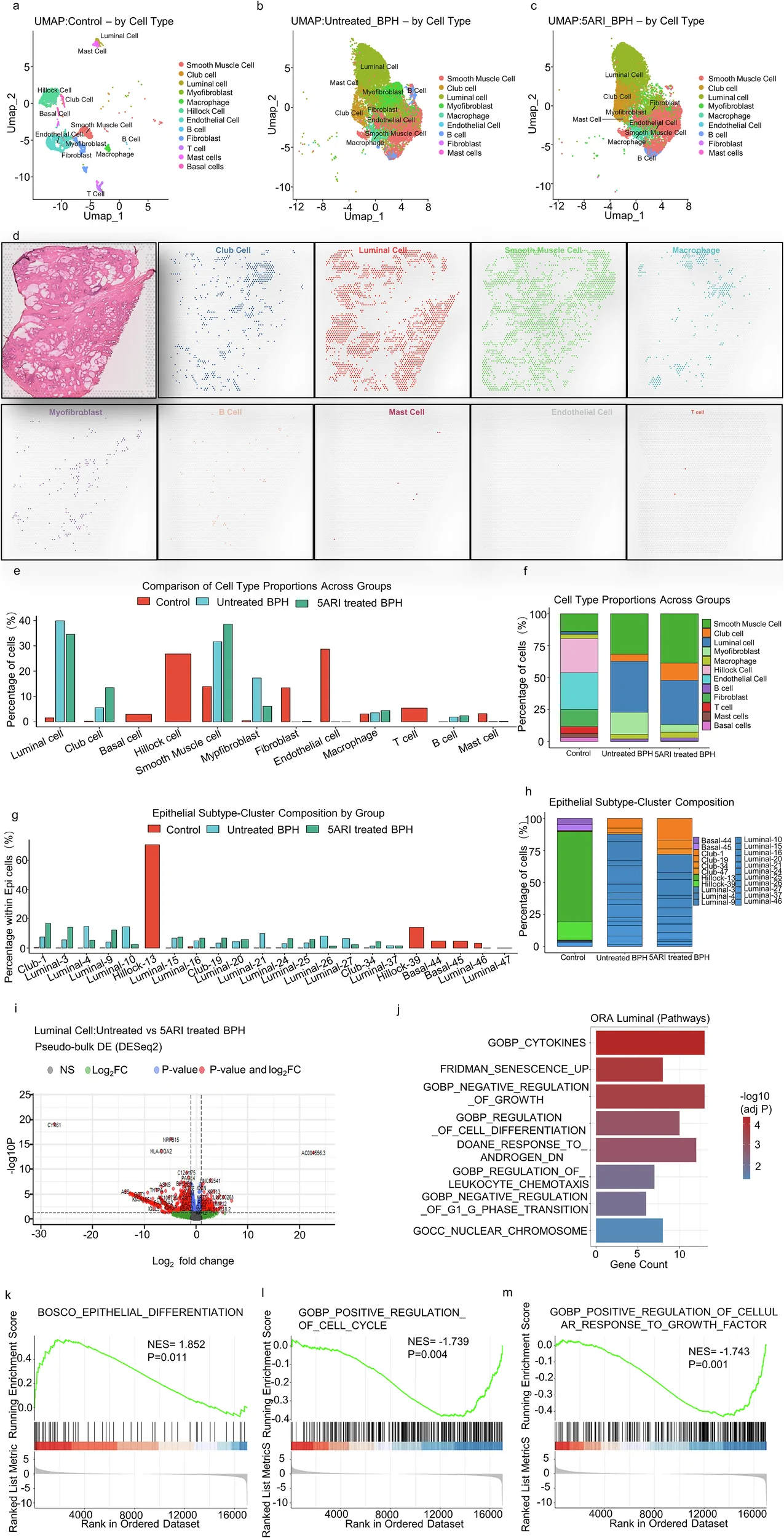
Cellular landscape of BPH and 5-ARI-associated lineage remodeling. **a**–**c**, UMAPs of the three groups colored by cell type: controls (*n* = 2; 6128 spots) (**a**), untreated BPH (*n *= 6; 20,072 spots) (**b**) and BPH after 5-ARI (*n* = 6; 19,976 spots) (**c**). Epithelial, immune and stromal compartments are cleanly separated in all groups. **d** Representative histology and its mapping to ST, validating cell-type annotations and spatial localization (examples: club, luminal, smooth muscle cell, macrophage, myofibroblast, B cell, mast cell, endothelial cell and T cell). Scale bars as indicated. **e**,**f** Cell-type compositions: group means ± s.e.m. (bars) with individual donors (dots) (**e**); stacked compositions per group (**f**). Group means (%, control → untreated BPH → 5-ARI): SMC 13.9 → 31.6 → 38.6; myofibroblast 0.47 → 17.3 → 6.1; luminal 1.58 → 39.9 → 34.5; club 0.26 → 5.59 → 13.5; B cell 0.05 → 1.88 → 2.38; macrophage 3.12 → 3.57 → 4.47. Basal, hillock, endothelial and T cell fractions decline markedly in BPH versus control (2.97/26.8/28.7/5.42% approaching ~0%). **g**,**h** Epithelial subcluster composition: group means ± s.e.m. (dots represent donors) (**g**); stacked subcluster profiles highlighting a reduction of luminal-dominant subclusters and expansion of club-related subclusters under 5-ARI (**h**). **i**–**m** Transcriptional programs in luminal regions: pseudobulk DE (DESeq2; **i**), over-representation analysis (ORA; **j**) and preranked GSEA (**k**–**m**). Compared with untreated BPH, 5-ARI upregulates epithelial differentiation while downregulating cell-cycle and growth-factor response programs (normalized enrichment scores (NES) and nominal *P* shown). For **e**–**h** donor-level proportions were tested by Kruskal–Wallis with Dunn’s post hoc test. For** i**–**m** donor-level GSVA/pathway scores were compared by two-sided Wilcoxon rank-sum tests; pseudobulk differential expression used DESeq2, and multiple testing was controlled by Benjamini–Hochberg FDR.

We jointly analyzed 2 normal controls (6,128 spots), 6 untreated BPH cases (20,072 spots) and 6 post-5-ARI cases (19,976 spots), and visualized group-wise UMAPs and spatial maps (Fig. 1a–d). Cell-type localizations were consistent across groups, enabling quantitative composition comparisons at both compartment and epithelial-subcluster levels (Fig. 1e–h).

Compared with normal controls, untreated BPH showed expansion of stromal and epithelial compartments, including smooth muscle cells (13.9% → 31.6%), myofibroblasts (0.47% → 17.3%), luminal epithelium (1.58% → 39.9%) and club cells (0.26% → 5.59%). Within immune cells, B cells (0.05% → 1.88%) and macrophages (3.12% → 3.57%) increased modestly, whereas basal, hillock, endothelial and T cell fractions decreased.

After 5-ARI therapy, we observed a lineage shift characterized by reduced luminal fraction (39.9% → 34.5%) and increased club fraction (5.59% → 13.5%), together with further smooth muscle enrichment (31.6% → 38.6%). B cells and macrophages also increased (1.88% → 2.38% and 3.57% → 4.47%, respectively), whereas myofibroblasts decreased (17.3% → 6.1%). These shifts were supported by spatial maps and epithelial subcluster decomposition (Fig. 1d, g, h), indicating luminal-to-club remodeling under 5-ARI pressure alongside selective stromal/immune changes.

At the transcriptional level, donor-level pseudobulk differential analysis and pathway enrichment suggested coordinated state changes in luminal and club epithelia after 5-ARI therapy. Luminal regions showed increased differentiation programs with reduced cell-cycle and growth-factor responses (Fig. 1i–m), whereas club regions exhibited decreased apoptotic execution and enhanced DNA-damage sensing/repair programs (Supplementary Fig. 5a–e). Together, these data suggest that 5-ARI pressure shifts epithelial states from proliferation toward differentiation and stress adaptation, with club cells expanding and acquiring stronger survival features.

**Fig. 2:**
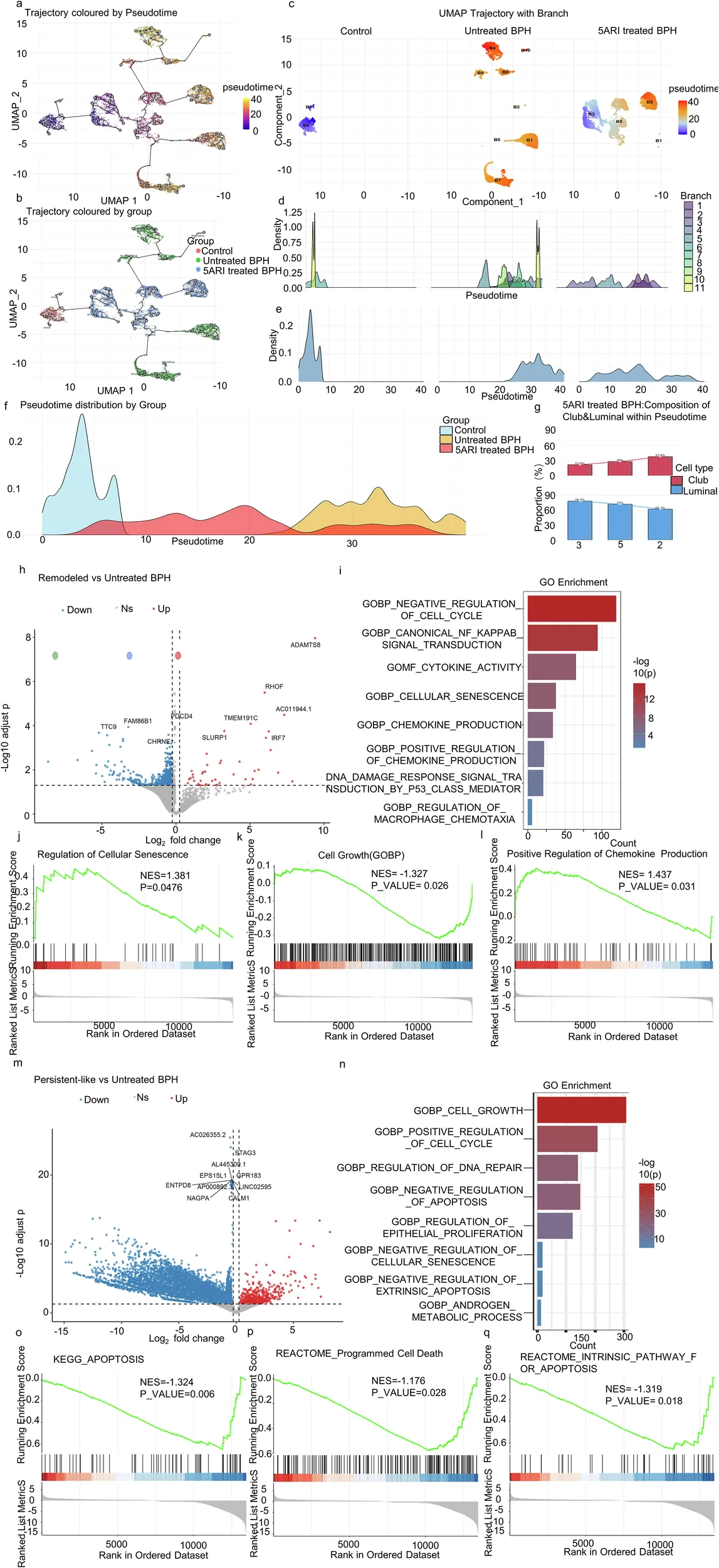
Spatial pseudotime reveals 5-ARI-associated epithelial remodeling states. **a**,**b** Spatial pseudotime rooted in the basal-enriched domain: UMAP colored by pseudotime (early → late) (**a**); the same embedding colored by group (control, BPH_NoDrug and BPH_Drug) (**b**). **c** Branch annotation showing post-treatment bifurcation into two 5-ARI-associated epithelial transcriptional states: a 5-ARI-remodeled branch distributed along the early/mid pseudotime trajectory, and a persistent/tolerant-like branch positioned near the untreated terminal state. **d**–**f** Kernel density of pseudotime: control peaks near the root; untreated BPH peaks at the terminus; the treated group is bimodal. **g** Along C3 → C5 → C2 in BPH_Drug, lineage composition shifts (open circles represent donors; bars indicate group mean ± s.e.m.): club increases while Luminal decreases. **h**–**l** 5-ARI-remodeled branch versus untreated BPH: volcano plots, ranked pathway bars and GSEA consistently show upregulated senescence and chemokine programs with downregulated cell-cycle/growth-factor responses. **m**–**q** persistent/tolerant-like branch versus untreated BPH: enrichment of anti-apoptotic/pro-growth and DNA damage sensing/repair programs. Donor-level summaries were used (control, *n* = 2; BPH_NoDrug, *n* = 6; BPH_Drug, *n* = 6; total, 46,176 spots). For **g**, donor-level proportions were tested using a two-sided Kruskal–Wallis test with Dunn’s post hoc test. For **h**–**q** donor-level pseudobulk differential expression was analyzed using DESeq2 with Benjamini–Hochberg FDR correction; pathway analyses were performed using GSVA/GSEA as described in the ‘Materials and methods’ section. Bars show group means ± s.e.m.; exact *P* and* q* values are reported in the corresponding panels and Source Data.

Spatial trajectory reconstruction rooted in basal-enriched domains revealed an ordered epithelial progression along pseudotime. Untreated BPH cells were concentrated near the trajectory terminus, whereas post-5-ARI samples bifurcated into two transcriptional arms: one distributed along the early/mid pseudotime trajectory, hereafter referred to as the 5-ARI-remodeled branch, and another overlapping the untreated terminal state, hereafter referred to as the persistent/tolerant-like branch (Fig. 2a–c). Consistently, pseudotime density profiles showed a control-at-root, untreated-at-terminus and treatment-bimodal pattern (Fig. 2d–f). Along the C3 → C5 → C2 axis within treated samples, club fractions increased while luminal fractions decreased, supporting luminal-to-club remodeling under drug pressure (Fig. 2g). Because this ST cohort is cross-sectional and lacks matched longitudinal clinical endpoints, these branch labels should be interpreted as inferred 5-ARI-associated epithelial transcriptional states rather than definitive clinical response categories. Functionally, the 5-ARI-remodeled branch was enriched for senescence-regulatory and chemokine programs with suppressed growth/cell-cycle pathways (Fig. 2h–l), whereas the persistent/tolerant-like branch showed enrichment of anti-apoptotic, pro-growth and DNA-damage/repair programs (Fig. 2m–q). Extended branch-to-other communication analyses further indicated strengthened epithelial–macrophage, epithelial–immune and epithelial–stromal crosstalk in the treated group. The 5-ARI-remodeled branch showed prominent outgoing signaling toward persistent/tolerant-like epithelial cells, macrophages, mast cells and stromal compartments, with MIF-, CXCL12-, laminin- and collagen-related pathways among the top signals (Supplementary Fig. 6a–f). The persistent/tolerant-like branch likewise exhibited strong outgoing signaling toward remodeled epithelial cells, mast cells, macrophages and stromal compartments, with enrichment of collagen-, APP-, laminin- and CXCL12-related communication programs (Supplementary Fig. 6g–l). Macrophage-side analyses further showed activation of IL-1β production, canonical NF-κB signaling and LPS-like inflammatory cascades (Supplementary Fig. 7a–e). Collectively, these data spatially link 5-ARI-associated epithelial state heterogeneity to inflammatory and survival-associated intercellular signaling, providing mechanistic context for lineage remodeling and tolerance-like epithelial adaptation. To further validate the spatial organization of the MIF–CD74/GSDMD and HMGB1/GSDMD–AGER epithelial–macrophage axes, we combined multiplex IF with ST-based nearest-neighbor analysis. At the protein level, 5-ARI-treated BPH showed increased MIF signal in KRT8+ luminal epithelial regions and a higher fraction of CD68^+^CD74^+^GSDMD-N^+^ macrophages compared with untreated BPH (Supplementary Fig. 8a–c). Spatial quantification showed a reduced nearest-neighbor distance between MIF^+^KRT8^+^ epithelial regions and CD68^+^CD74^+^GSDMD-N^+^ macrophages in 5-ARI-treated BPH tissues (Supplementary Fig. 8d). Similarly, 5-ARI-treated BPH tissues showed increased AGER signal in KRT8^+^ luminal epithelial regions and enrichment of CD68^+^GSDMD-N^+^HMGB1^+^ macrophages (Supplementary Fig. 8e–g), accompanied by a shorter nearest-neighbor distance between CD68^+^GSDMD-N^+^HMGB1^+^ macrophages and KRT8^+^AGER^+^ epithelial regions (Supplementary Fig. 8h).

To address whether the inferred ligand–receptor interactions could reflect mixed-spot co-expression, we calculated distances between distinct ligand-enriched sender spots and receptor/state-enriched receiver spots in ST sections with available spatial-coordinate files. MIF-high epithelial-enriched spots were located closer to CD74/GSDMD-high macrophage-enriched spots than the corresponding epithelial-to-macrophage background distance (Supplementary Fig. 8i). HMGB1/GSDMD-high macrophage-enriched spots were also closer to AGER-high epithelial-enriched spots than the macrophage-to-epithelial background distance (Supplementary Fig. 8j). Corresponding single-channel images were provided as an independent channel-separation control and confirmed marker-specific localization for both multiplex IF panels (Supplementary Fig. 9a,b). Together, these imaging and ST analyses support organized epithelial–macrophage neighborhoods involving the MIF–CD74/GSDMD and HMGB1/GSDMD–AGER axes, rather than ligand–receptor signals arising solely from same-spot co-expression.

### Enhanced epithelial–macrophage crosstalk under 5-ARI: MIF–CD74 drives activation/pyroptosis, CXCL12–CXCR4 mediates chemotaxis

**Fig. 3:**
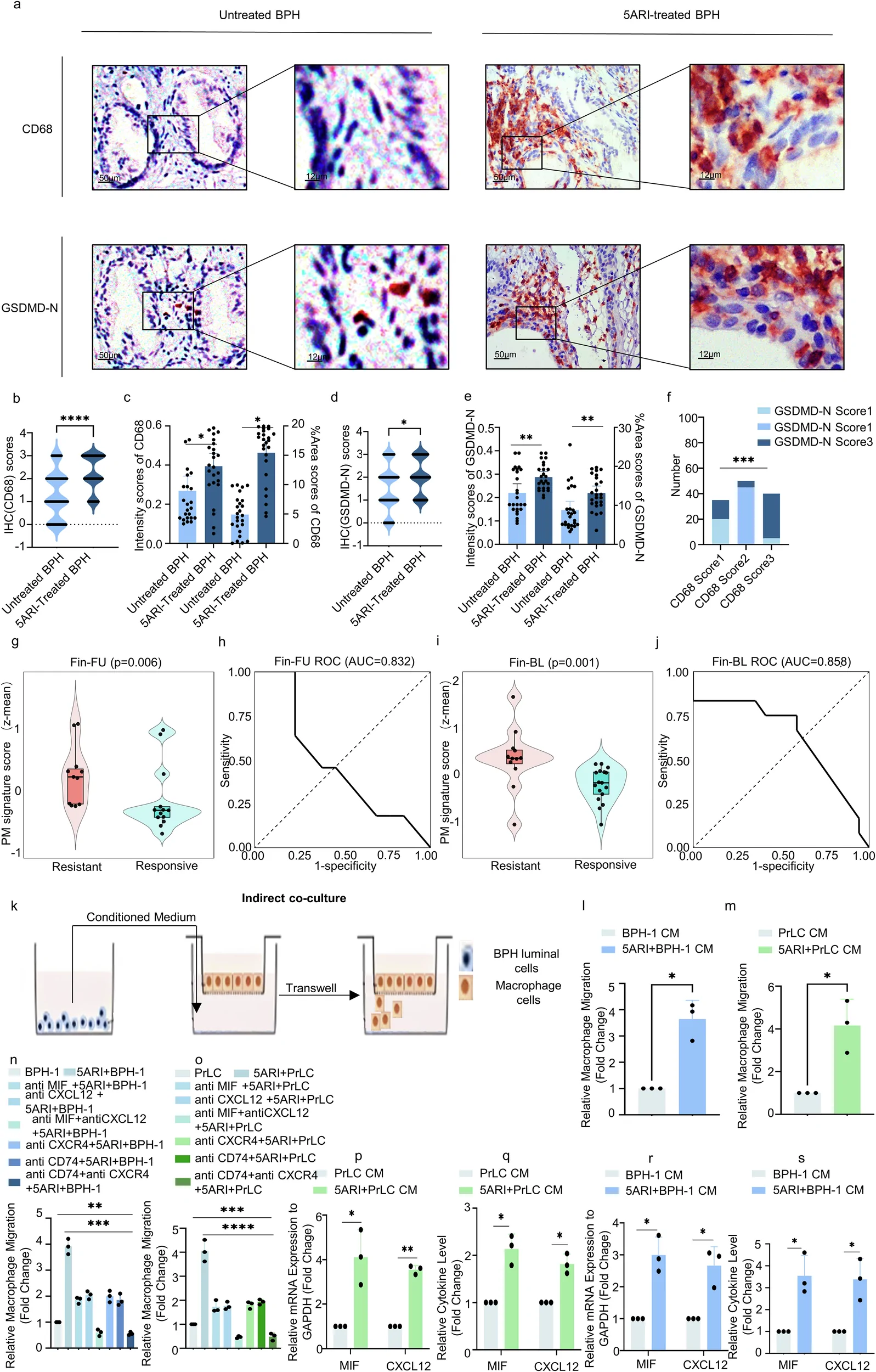
Strengthened ‘pyroptosis–macrophage axis’ under 5-ARI: histology, independent cohort and in vitro evidence. **a** Representative IHC of untreated BPH (left) and BPH after 5-ARI (right). Top: CD68; bottom: GSDMD-N; right panels show magnified insets. Scale bars, 50 μm and 12 μm.** b**–**e** Patient-level IHC quantification in 25 untreated patients with BPH and 25 independent 5-ARI-treated patients with BPH. Each dot represents one patient-level value obtained by averaging five non-overlapping fields; bars show mean ± s.e.m. CD68 semiquantitative IHC mean score on a 0–3 scale (**b**); cCD68 staining intensity and percentage positive area (**c**); GSDMD-N semiquantitative IHC mean score on a 0–3 scale (**d**); GSDMD-N staining intensity and percentage positive area (**e**). Patient-level semiquantitative scores were compared using two-sided Mann–Whitney *U* tests, whereas staining-intensity and percentage-positive-area measurements were compared using two-sided Welch’s unpaired *t*-tests. Exact *P* values are provided in the Source Data file. **f**, GSDMD-N grade distribution stratified by CD68 score (counts per score 1/2/3); higher CD68 strata coincide with higher GSDMD-N grades. **g**–**j** Validation in the independent cohort GSE262946 using the PM-13 gene signature (Pyro⊕Mac): signature scores at follow-up (Fin-FU; resistant versus responsive, *P *= 0.006) (**g**); ROC (AUC 0.832) (**h**); baseline (Fin-BL) signature scores (*P *= 0.001) (**i**); ROC (AUC 0.858) (**j**).** k** Schematic of indirect co-culture/CM: finasteride (10 μM, 48 h) treatment of BPH-1 or PrLCs to generate 5-ARI-CM, followed by Transwell assays on macrophages. **l**,**m** 5-ARI-CM enhances macrophage migration: relative to matched controls, migration increases with BPH-1-CM and PrLC-CM (normalized to Ctrl-CM = 1). **n**,**o** Blockade experiments: neutralizing MIF or CXCL12, or blocking CD74 or CXCR4, partially reduces migration; dual blockade (MIF + CXCL12 or CD74 + CXCR4) produces stronger suppression. **p**–**s** Epithelial readouts: 5-ARI elevates epithelial MIF and CXCL12 at both mRNA and secreted protein levels (qPCR versus GAPDH; ELISA/multiplex as fold change to control), consistent with the migration phenotype and the implicated ligand–receptor axes. IHC analyses included 25 untreated patients with BPH and 25 independent 5-ARI-treated patients with BPH. For each patient, measurements from five non-overlapping fields were averaged to generate one patient-level value. Patient-level semiquantitative IHC mean scores were compared using two-sided Mann–Whitney *U* tests. Patient-level staining-intensity and percentage-positive-area measurements were compared using two-sided Welch’s unpaired *t*-tests. Field-level measurements were not treated as independent statistical observations. Exact *P* values and individual patient-level values are provided in the Source Data file.

We first performed IHC on FFPE prostate sections from 25 untreated patients with BPH and 25 independent patients after 5-ARI therapy. Macrophage infiltration and pyroptosis were assessed using CD68 and GSDMD-N staining, respectively. Quantification included patient-level semiquantitative IHC scores on a 0–3 scale, staining intensity and percentage positive area. For semiquantitative scoring, five non-overlapping fields were evaluated for each patient and averaged to generate one patient-level mean score. The 5-ARI-treated group showed increased CD68 and GSDMD-N staining across the corresponding quantitative readouts (Fig. 3a–e). Stratification by CD68 score demonstrated a right-shifted ordinal distribution in the 5-ARI group, and higher CD68 strata aligned with higher GSDMD-N grades (Fig. 3f).

To validate this ‘pyroptosis–macrophage’ signal in an independent cohort, we analyzed bulk RNA-seq data from GSE262946 (Fin-BL and Fin-FU; resistant versus responsive; Fin-BL: *n* = 29 (12 resistant, 17 responsive); Fin-FU:* n* = 24 (11 resistant, 13 responsive)). Using nested cross-validation with sequential forward floating selection over a prespecified candidate pool, we derived a 13-gene panel (PM-13: NLRP3, CASP1, CSF1R, CD68, SOD2, IL27, IRF1, CCL2, IL12B, OAS2, CCL7, NLRP2 and TNF). PM-13 discriminated resistant from responsive cases at both time points (Fin-FU: *P* = 0.006469, AUC 0.832; Fig. 3g,h; Fin-BL: *P* = 0.001326, AUC 0.858; Fig. 3i,j).

We next tested whether 5-ARI-conditioned epithelia promote macrophage recruitment via soluble cues. BPH-1 cells or PrLCs were treated with finasteride (10 μM, 48 h), and CM (5-ARI-CM) were applied in Transwell chemotaxis assays with targeted blockade (Fig. 3k). Relative to matched controls, 5-ARI-CM increased macrophage migration by ~3.6-fold (BPH-1-CM) and ~4.1-fold (PrLC-CM) (Fig. 3l,m). Neutralization of MIF or blockade of its receptor CD74, and neutralization of CXCL12 or blockade of CXCR4, each partially reduced migration (~1.8–2.0-fold). Dual blockade (MIF + CXCL12 or CD74 + CXCR4) further suppressed migration toward baseline (to ~0.60 and ~0.56, respectively; Fig. 3n,o). Consistently, finasteride increased epithelial MIF and CXCL12 at both mRNA and secreted protein levels (Fig. 3p–s), matching the strengthened epithelial → macrophage interactions inferred from spatial analyses.

Using the same CM exposure paradigm, we quantified macrophage inflammatory/pyroptotic outputs by soluble readouts (LDH, IL-1β, IL-18 and HMGB1). Compared with Ctrl-CM, 5-ARI-CM increased LDH release (~3.3–3.7-fold) and elevated IL-1β, IL-18 and HMGB1 (Supplementary Fig. 10a–h). Neutralizing MIF or blocking CD74 significantly reduced these release markers, with combined blockade approaching baseline, whereas CXCL12 or CXCR4 blockade did not significantly affect these pyroptosis-associated outputs (Supplementary Fig. 10a–h). Immunoblotting further confirmed activation of the pyroptotic axis (increased NLRP3, cleaved caspase-1 p20 and GSDMD-N) and enhanced HMGB1/IL-1β/IL-18, all of which were attenuated by anti-MIF or anti-CD74 in both BPH-1-CM and PrLC-CM systems (Supplementary Fig. 10i–v).

Microscopy and flow cytometry yielded concordant results. PI/Hoechst staining showed a marked increase in PI^+^ cells after 5-ARI-CM exposure, which was reduced by anti-MIF or anti-CD74 (Supplementary Fig. 10w–z). In flow cytometry, active caspase-1^+^PI^+^ double positivity increased to ~25% with 5-ARI-CM and was reduced to near-baseline by MIF neutralization or CD74 blockade (Supplementary Fig. 10z1–z4).

To further assess whether 5-ARI-conditioned epithelial secretomes induced a broader macrophage functional-state shift, we measured selected M1-like inflammatory and M2-like remodeling/regulatory macrophage markers in hMDMs exposed to Ctrl-CM or 5-ARI-CM from BPH-1 cells or primary PrLCs. In BPH-1-CM–treated macrophages, 5-ARI-CM increased M1-like inflammatory transcripts, including TNF, CXCL10 and CD86, to 3.00 ± 0.07-fold, 1.99 ± 0.12-fold and 2.04 ± 0.05-fold, respectively, relative to Ctrl-CM (Supplementary Fig. 11a). By contrast, M2-like remodeling/regulatory markers, including MRC1, CD163, IL10 and CCL18, showed weaker and nonsignificant changes (1.09 ± 0.14-fold, 1.06 ± 0.18-fold, 1.08 ± 0.19-fold and 1.10 ± 0.22-fold, respectively; Supplementary Fig. 11b). A similar pattern was observed using PrLC-derived CM: 5-ARI-PrLC-CM increased TNF, CXCL10 and CD86 transcripts to 2.53 ± 0.19-fold, 3.19 ± 0.13-fold and 2.02 ± 0.08-fold, respectively (Supplementary Fig. 11c), whereas MRC1, CD163, IL10 and CCL18 showed no consistent induction (0.60 ± 0.09-fold, 0.78 ± 0.29-fold, 0.64 ± 0.12-fold and 0.93 ± 0.16-fold, respectively; Supplementary Fig. 11d).

Consistently, ELISA-based secretory profiling showed that 5-ARI-BPH-1-CM increased relative secretion of the inflammatory cytokines/chemokines TNF, IL-6, and CXCL10 to 3.38 ± 0.06-fold, 2.67 ± 0.06-fold and 2.68 ± 0.08-fold, respectively (Supplementary Fig. 11e), whereas the remodeling/regulatory factors IL10, TGF-β1 and CCL18 remained weakly changed or heterogeneous (0.81 ± 0.04-fold, 0.78 ± 0.30-fold and 0.78 ± 0.27-fold, respectively; Supplementary Fig. 11f). 5-ARI-PrLC-CM similarly increased TNF, IL-6 and CXCL10 secretion to 4.45 ± 0.25-fold, 3.08 ± 0.08-fold and 2.89 ± 0.14-fold, respectively (Supplementary Fig. 11g), whereas IL10, TGF-β1 and CCL18 showed no consistent induction (1.07 ± 0.16-fold, 0.86 ± 0.17-fold and 0.63 ± 0.14-fold, respectively; Supplementary Fig. 11h). Together with the pyroptosis-associated outputs described above, these data indicate that 5-ARI-conditioned epithelial secretomes induce M1-like inflammatory features in hMDMs, but do not define a fixed canonical M1 or M2 polarization program. We therefore interpret CM-exposed macrophages as acquiring an inflammatory/pyroptotic effector-like state.

Together, these data indicate that 5-ARI-treated epithelial cells recruit macrophages through combined MIF–CD74 and CXCL12–CXCR4 chemotactic signaling, but selectively activate macrophage pyroptosis predominantly through the MIF–CD74 axis. This druggable epithelial–macrophage crosstalk amplifies a pyroptosis–inflammation cascade and provides a mechanistic basis for microenvironmental remodeling and tolerance-like adaptation after 5-ARI therapy.

### Defining and testing the HMGB1–AGER axis as a major mediator of epithelial senescence, G1 arrest and proliferation restraint

**Fig. 4:**
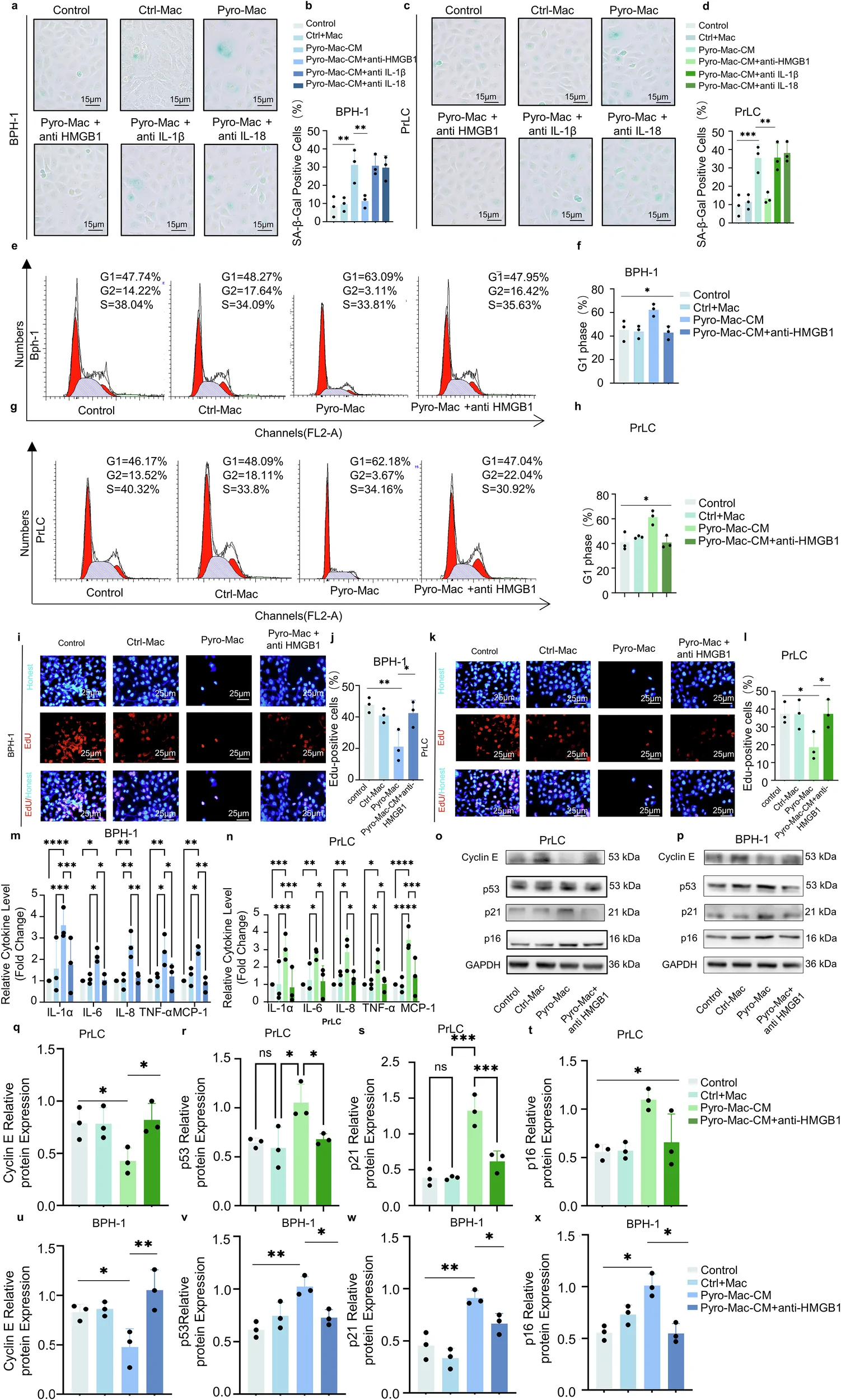
Pyroptotic macrophage CM (Pyro-CM) induce epithelial senescence, G1 arrest and proliferation restraint via HMGB1; neutralization of HMGB1 rescues. **a**,**b** SA-β-gal staining and quantification in BPH-1 cells under control, Ctrl-Mac, Pyro-CM, Pyro-CM+anti-HMGB1, Pyro-CM+anti-IL-1β and Pyro-CM+anti-IL-18 conditions. Scale bar, 15 μm. **c**,**d** SA-β-gal staining and quantification in primary PrLCs under the same conditions. Scale bar, 15 μm. **e**,**f** DNA-content flow cytometry and G1-phase fraction in BPH-1 cells under control, Ctrl-Mac, Pyro-CM and Pyro-CM+anti-HMGB1 conditions. **g**,**h** DNA-content flow cytometry and G1-phase fraction in primary PrLCs under the same conditions. **i**,**j** EdU/Hoechst imaging and EdU-positive rate in BPH-1 cells under control, Ctrl-Mac, Pyro-CM and Pyro-CM+anti-HMGB1 conditions. Scale bar, 25 μm. **k**,**l** EdU/Hoechst imaging and EdU-positive rate in primary PrLCs under the same conditions. Scale bar, 25 μm. **m**,**n** Multiplex cytokine readouts showing relative levels of IL-1α, IL-6, IL-8, TNF and MCP-1 in BPH-1 cells and PrLCs, respectively. **o**,**p** Representative immunoblots for Cyclin E, p53, p21 and p16 in PrLCs and BPH-1 cells, respectively.** q**–**t** Densitometric quantification of Cyclin E, p53, p21 and p16 in PrLCs, normalized to the corresponding loading control. **u**–**x** Densitometric quantification of Cyclin E, p53, p21 and p16 in BPH-1 cells, normalized to the corresponding loading control.Bars/dots show mean ± s.e.m.; *n* = 3 independent biological replicates unless otherwise indicated. Multigroup analyses were performed using the panel-specific omnibus and multiplicity-adjusted post hoc tests described below. For **m**, simple effects within each cytokine row were assessed using Dunnett’s multiple-comparisons test. For **n**, ordinary one-way ANOVA was followed by Dunnett’s multiple-comparisons test, with Pyro-CM specified as the reference group. Panel **s** was analyzed using ordinary one-way ANOVA followed by Šídák’s multiple-comparisons test. The remaining densitometric panels were analyzed using ordinary one-way ANOVA followed by Tukey’s multiple-comparisons test. Significance: ns, not significant; *P < 0.05, **P < 0.01, ***P < 0.001, ****P < 0.0001. Exact* P* values are provided in the Source Data file.

Under 5-ARI conditions, we used BPH-1 and primary PrLCs to compare epithelial responses to control CM (Control/Ctrl-Mac), pyroptotic macrophage CM (Pyro-CM), and Pyro-CM with HMGB1 neutralization (Fig. 4a–x). Pyro-CM increased SA-β-gal positivity by ~3–4-fold, whereas anti-HMGB1 largely restored staining toward control; neutralizing IL-1β or IL-18 had minimal effects (Fig. 4a–d). Consistently, Pyro-CM induced a pronounced G1 accumulation (+15–20 percentage points) that was substantially reversed by HMGB1 blockade (Fig. 4e–h), and reduced EdU incorporation to ~50% of control, which was significantly rescued by anti-HMGB1 (Fig. 4i–l). Pyro-CM also elevated SASP output (IL-1α, IL-6, IL-8, TNF and MCP-1; ~2–4-fold), broadly regressing toward baseline with HMGB1 neutralization (Fig. 4m,n). At the molecular level, Pyro-CM reduced Cyclin E and activated the p53–p21/p16 axis, changes that were attenuated by anti-HMGB1 (Fig. 4o–x). Together, pyroptotic macrophages impose a largely HMGB1-dependent senescence program in luminal epithelia—encompassing SA-β-gal positivity, G1 arrest, proliferation restraint, SASP induction and canonical checkpoint activation—supporting HMGB1 as a key effector of epithelial remodeling under 5-ARI.

**Fig. 5:**
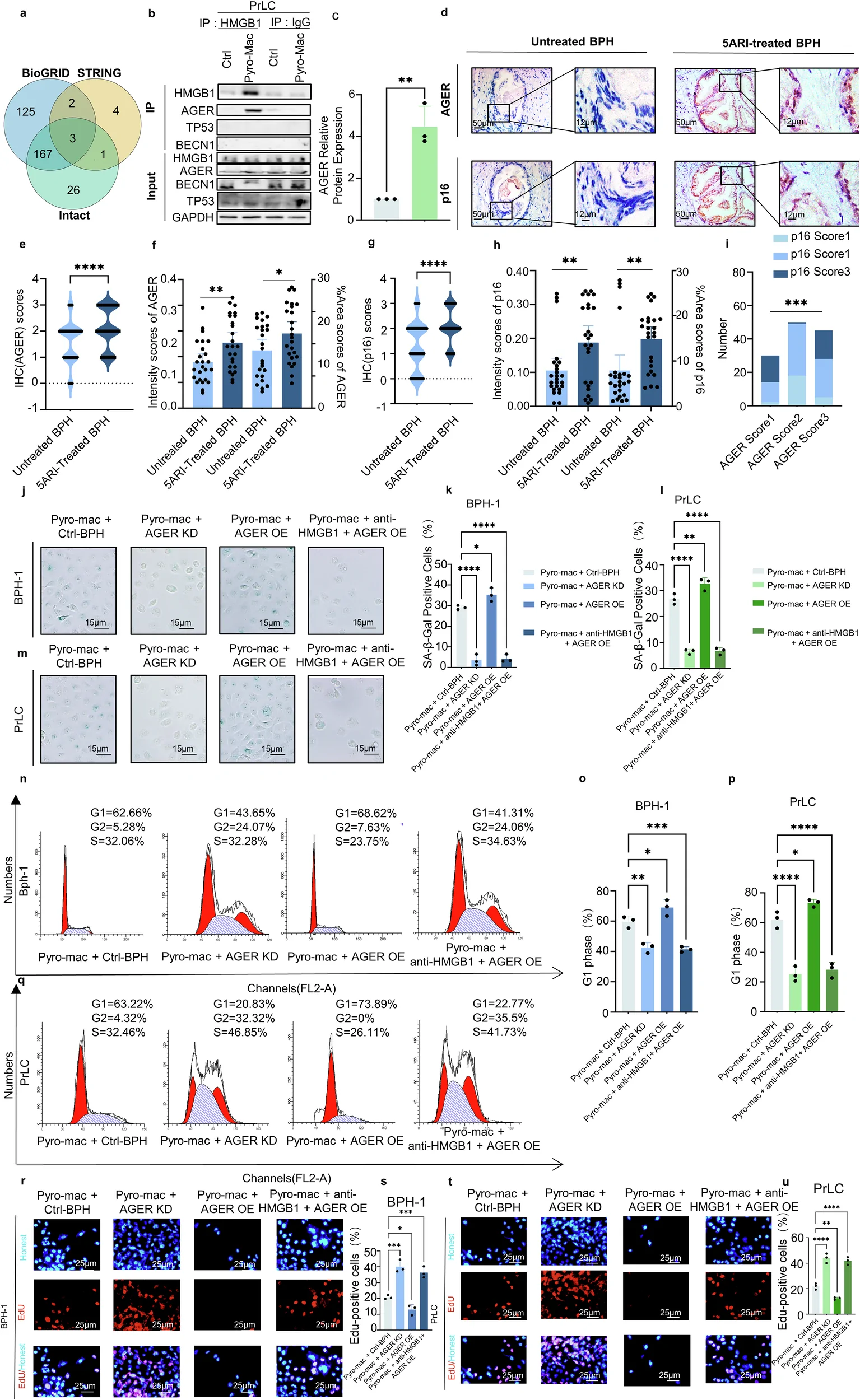
The HMGB1–AGER axis causally links pyroptotic macrophages to epithelial senescence and G1 arrest. **a** Venn diagram of HMGB1 downstream interactors from BioGRID, IntAct and STRING (intersection). **b** Co-immunoprecipitation in primary luminal epithelia (PrLC; IP: HMGB1; IgG negative control) with inputs: AGER co-precipitates with HMGB1, whereas TP53 and BECN1 are not detected; Pyro-Mac/Pyro-CM stimulation increases AGER and TP53 in input lanes. **c** Quantification of AGER protein (mean ± s.e.m.). **d** Representative IHC on consecutive sections: AGER and p16 in untreated BPH versus BPH after 5-ARI; right panels show magnified insets. Scale bars, 50 μm and 12 μm. **e**–**h** Patient-level IHC quantification of AGER and p16 in 25 untreated BPH patients and 25 independent 5-ARI-treated BPH patients. Each dot represents one patient-level value obtained by averaging five non-overlapping fields. AGER semiquantitative IHC mean score on a 0–3 scale (**e**); AGER staining intensity and percentage positive area (**f**); p16 semiquantitative IHC mean score on a 0–3 scale (**g**); p16 staining intensity and percentage positive area (**h**). Bars show mean ± s.e.m. **i** Ordinal distribution of p16 staining scores. **j**,**m** SA-β-gal micrographs (BPH-1; PrLC) under control, AGER knockdown (KD), AGER overexpression (OE) and anti-HMGB1 + AGER OE. Scale bar, 15 μm. **k**,**l** SA-β-gal positivity (%; BPH-1; PrLC). **n**,**q** DNA-content histograms with overlaid G1/S/G2 fractions (same groupings). **o**,**p** G1 fraction statistics (BPH-1; PrLC). **r**,**t** EdU/Hoechst imaging (BPH-1; PrLC). Scale bar, 25 μm. **s**,**u** EdU positivity (%; BPH-1; PrLC). For the human-tissue IHC analyses, patient-level semiquantitative scores were compared using two-sided Mann–Whitney *U* tests. Patient-level staining-intensity and percentage-positive-area measurements were compared using two-sided Welch’s unpaired *t*-tests. In vitro two-group and multigroup comparisons were performed using the panel-specific statistical procedures described in the ‘Materials and methods’ section. Exact *P* values and individual values are provided in the Source Data file.

To identify the functional epithelial receptor for pyroptosis-released HMGB1, we cross-referenced experimentally supported interactions from BioGRID, IntAct, and STRING, yielding overlapping candidates AGER, TP53 and BECN1 (Fig. 5a). In PrLCs, co-immunoprecipitation (IP: HMGB1) detected co-precipitation of AGER with HMGB1, whereas TP53 and BECN1 did not show stable complex formation; input controls indicated increased AGER (and TP53) protein after pyro-macrophage stimulation (Fig. 5b). AGER upregulation was confirmed by immunoblot quantification (Fig. 5c). In patient tissues, epithelial AGER and p16 were higher in 5-ARI-treated BPH than in untreated BPH (Fig. 5d–h), with a right-shifted distribution of p16 grades in the treated group (Fig. 5i), consistent with reinforcement of the HMGB1–AGER axis in vivo.

We next tested causality by modulating AGER in BPH-1 and PrLCs exposed to pyro-macrophage CM. Pyro-mac CM robustly increased SA-β-gal positivity, which was reduced by AGER knockdown and further increased by AGER overexpression; importantly, adding anti-HMGB1 in the overexpression setting largely reverted senescence toward control/knockdown levels (Fig. 5j–m). Cell-cycle profiling showed concordant effects: pyro-mac CM induced G1 arrest, relieved by AGER knockdown and exacerbated by AGER overexpression, with HMGB1 neutralization under overexpression markedly de-repressing G1 accumulation (Fig. 5n–q). EdU incorporation mirrored these phenotypes, with knockdown rescuing DNA synthesis, overexpression suppressing proliferation, and HMGB1 neutralization partially restoring EdU positivity in the overexpression background (Fig. 5r–u). Collectively, these results establish a ligand–receptor mechanism in which macrophage pyroptosis-derived HMGB1 signals through epithelial AGER to drive senescence, G1 arrest and proliferation restraint; blocking either HMGB1 or AGER mitigates the phenotype, whereas receptor overexpression amplifies it in an HMGB1-dependent manner.

Recombinant HMGB1 increased SA-β-gal positivity in both BPH-1 cells and PrLCs, and this induction was attenuated by AGER knockdown (Supplementary Fig. 12a–d). Consistently, recombinant HMGB1 induced G1-phase accumulation, which was reduced by AGER knockdown (Supplementary Fig. 12e–h). EdU incorporation showed the reciprocal pattern: recombinant HMGB1 suppressed DNA synthesis, while AGER knockdown restored EdU positivity toward baseline levels (Supplementary Fig. 12i–l). In parallel, recombinant HMGB1 increased SASP-associated cytokine outputs, including IL-1α, IL-6, IL-8, TNF and MCP-1, and these responses were reduced by AGER knockdown in both cell models (Supplementary Fig. 12m,n).

These gain-of-ligand/loss-of-receptor experiments indicate that HMGB1 is sufficient to induce key features of epithelial senescence and proliferation restraint, and that epithelial AGER mediates a major component of the HMGB1-induced response. Together with HMGB1 neutralization in Pyro-CM and AGER perturbation under pyro-macrophage stimulation, these data support HMGB1–AGER as a major functional axis among the tested pyroptosis-associated mediators rather than merely a correlative ligand–receptor pair.

### Paracrine reprogramming by senescent epithelia: promoting luminal→club conversion and suppressing apoptosis

**Fig. 6:**
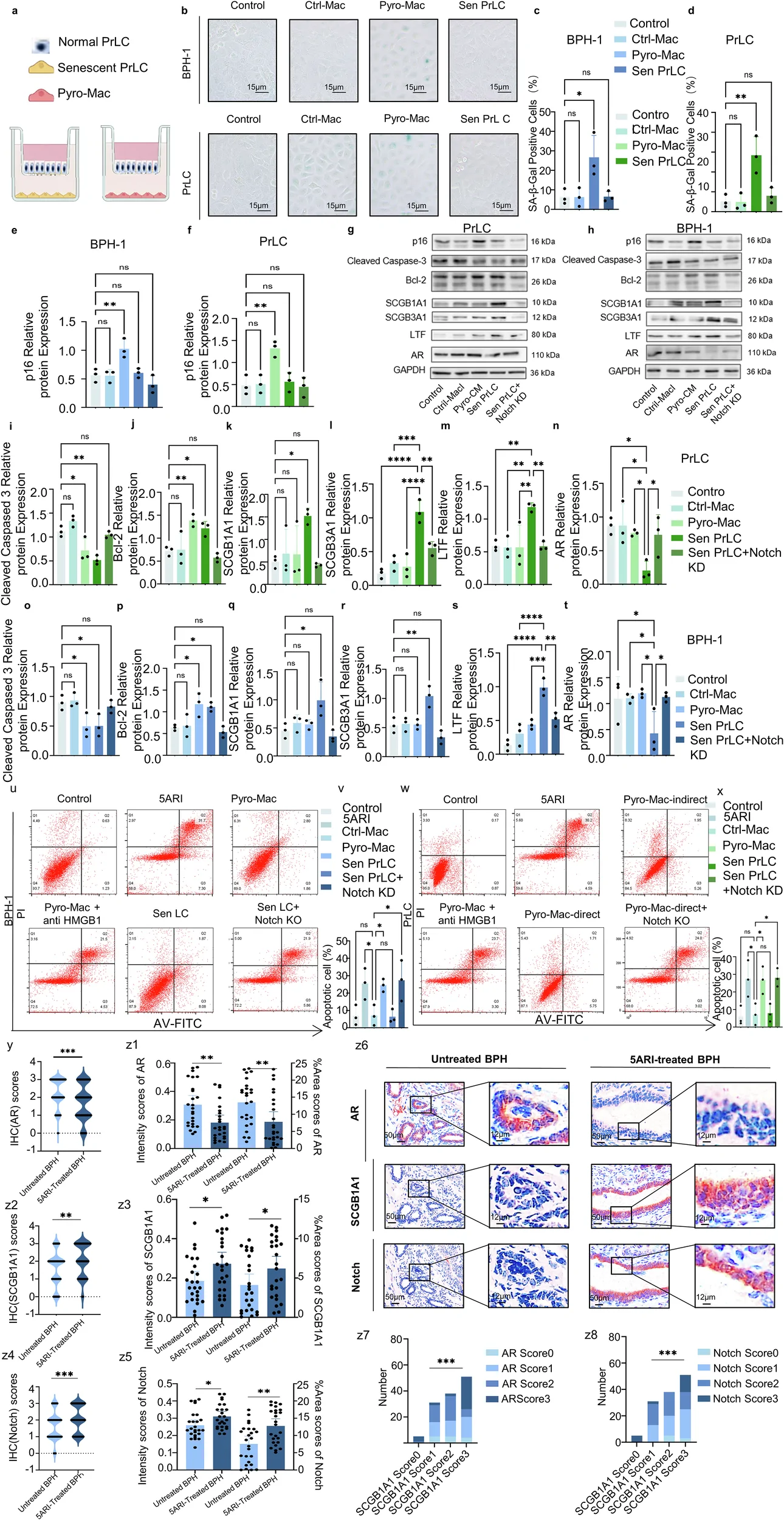
Paracrine reprogramming by senescent epithelia promotes luminal-to-club conversion and suppresses apoptosis through upstream HMGB1 signaling and a partially Notch-associated recipient-cell program. **a** Schematic of the experimental design. In system 1, recipient luminal epithelial cells were exposed to pyroptotic macrophage CM (Pyro-CM) to assess macrophage-derived effects. In system 2, recipient luminal epithelial cells were co-cultured with Pyro-CM–induced senescent PrLCs (Sen-PrLC) to assess secondary paracrine effects from senescent luminal cells. Where indicated, HMGB1 was neutralized in the Pyro-CM condition, and NOTCH1 was knocked down in recipient luminal epithelial cells. **b**–**d** SA-β-gal staining and quantification in BPH-1 cells and primary PrLCs. Pyro-CM increased SA-β-gal-positive cells, whereas HMGB1 neutralization attenuated this effect. Sen-PrLC did not further propagate senescence in recipient luminal cells. **e**–**t** Representative immunoblots and densitometric quantification of senescence, apoptosis, club-cell and luminal markers in BPH-1 cells and PrLCs under the indicated conditions. Quantified proteins include p16, cleaved caspase-3, Bcl-2, SCGB1A1, SCGB3A1, LTF and AR. Pyro-CM increased p16 and Bcl-2 and reduced cleaved caspase-3, consistent with a senescence-associated anti-apoptotic state. Sen-PrLC induced a club-like program, including increased SCGB1A1, SCGB3A1, and LTF and reduced AR. Recipient-cell NOTCH1 knockdown partially reversed Sen-PrLC-induced club-marker expression and AR suppression. **u**–**x** Annexin V/PI flow-cytometric analysis of apoptosis in BPH-1 cells and PrLCs. Direct 5-ARI exposure increased apoptosis, whereas Pyro-CM and Sen-PrLC reduced apoptosis. HMGB1 neutralization restored apoptosis in the Pyro-CM condition, and recipient-cell NOTCH1 knockdown restored apoptosis in the Sen-PrLC condition. **y**,**z1** Patient-level IHC quantification of AR in 25 untreated BPH patients and 25 independent 5-ARI-treated patients with BPH: the AR semiquantitative IHC mean score on a 0–3 scale (**y**), and AR staining intensity and percentage positive area (**z1**). **z2**,**z3** Patient-level IHC quantification of SCGB1A1 in the same patients: the SCGB1A1 semiquantitative IHC mean score on a 0–3 scale (**z2**), and SCGB1A1 staining intensity and percentage positive area (**z3**). **z4**,**z5** Patient-level IHC quantification of Notch in the same patients: the Notch semiquantitative IHC mean score on a 0–3 scale (**z4**), and Notch staining intensity and percentage positive area (**z5**). **z6** Representative IHC images of AR, SCGB1A1 and Notch in untreated BPH and 5-ARI-treated BPH tissues. **z7**,**z8** Ordinal association analyses in human BPH tissues: the relationship between SCGB1A1 score and AR score (**z7**), and the relationship between SCGB1A1 score and Notch score (**z8**). For semiquantitative IHC analyses, five fields were averaged to generate one patient-level mean score. Patient-level semiquantitative scores were compared using two-sided Mann–Whitney *U* tests; staining-intensity and percentage-positive-area measurements were compared using two-sided Welch’s unpaired *t*-tests. Each dot represents one independent patient.

To separate the direct effects of pyroptotic macrophages from the secondary paracrine effects of the senescent epithelia they induce, we established two Transwell systems (Fig. 6a). In system 1, recipient luminal cells (BPH-1 or primary PrLCs) were cultured above pyroptotic macrophages (pyro-mac) to generate Pyro-CM. In system 2, recipient luminal cells were cultured above Pyro-CM-induced senescent PrLCs (Sen-PrLC) placed in the lower chamber. Where indicated, NOTCH1 knockdown was performed only in recipient luminal cells (not in Sen-PrLC).

Pyro-CM robustly increased SA-β-gal positivity (~3–4-fold), whereas Sen-PrLC did not further propagate senescence; HMGB1 neutralization in the Pyro-CM setting largely restored SA-β-gal staining toward control (Fig. 6b–d). At the protein level, Pyro-CM increased p16 and Bcl-2 and reduced cleaved caspase-3, consistent with a ‘senescence + anti-apoptosis’ state, but did not consistently induce club markers (SCGB1A1/SCGB3A1/LTF). By contrast, Sen-PrLC drove a pronounced club-like reprogramming program in recipient luminal cells, with increased SCGB1A1/SCGB3A1/LTF and decreased AR, while maintaining anti-apoptotic features (Bcl-2 up; cleaved caspase-3 down). Recipient-side NOTCH1 knockdown partially reversed these club-like and AR-suppressive changes (Fig. 6e–t).

Functionally, both Pyro-CM and Sen-PrLC reduced the Annexin V/PI double-positive fraction, indicating suppression of apoptosis. Apoptosis was restored/enhanced by HMGB1 neutralization in the Pyro-CM condition and by recipient-side NOTCH1 knockdown in the Sen-PrLC condition; direct 5-ARI exposure served as a positive control and increased apoptosis (Fig. 6u–x). In patient tissues, quantitative IHC showed increased SCGB1A1, reduced AR and enhanced Notch signaling in the 5-ARI group, consistent with a therapy-associated luminal-to-club shift in vivo (Figs. 6y–z8).

Together, these data support a two-step model in which pyroptotic macrophages impose an HMGB1-dependent ‘senescence + anti-apoptosis’ state in luminal epithelia, and the resulting Sen-PrLC then paracrinely drives luminal-to-club reprogramming while sustaining apoptosis resistance. HMGB1 acts upstream, whereas Notch functions as a recipient-cell effector node, highlighting two actionable intervention points.

### 5-ARI treatment enriches pyroptotic macrophages in a castration/testosterone-induced BPH model

**Fig. 7:**
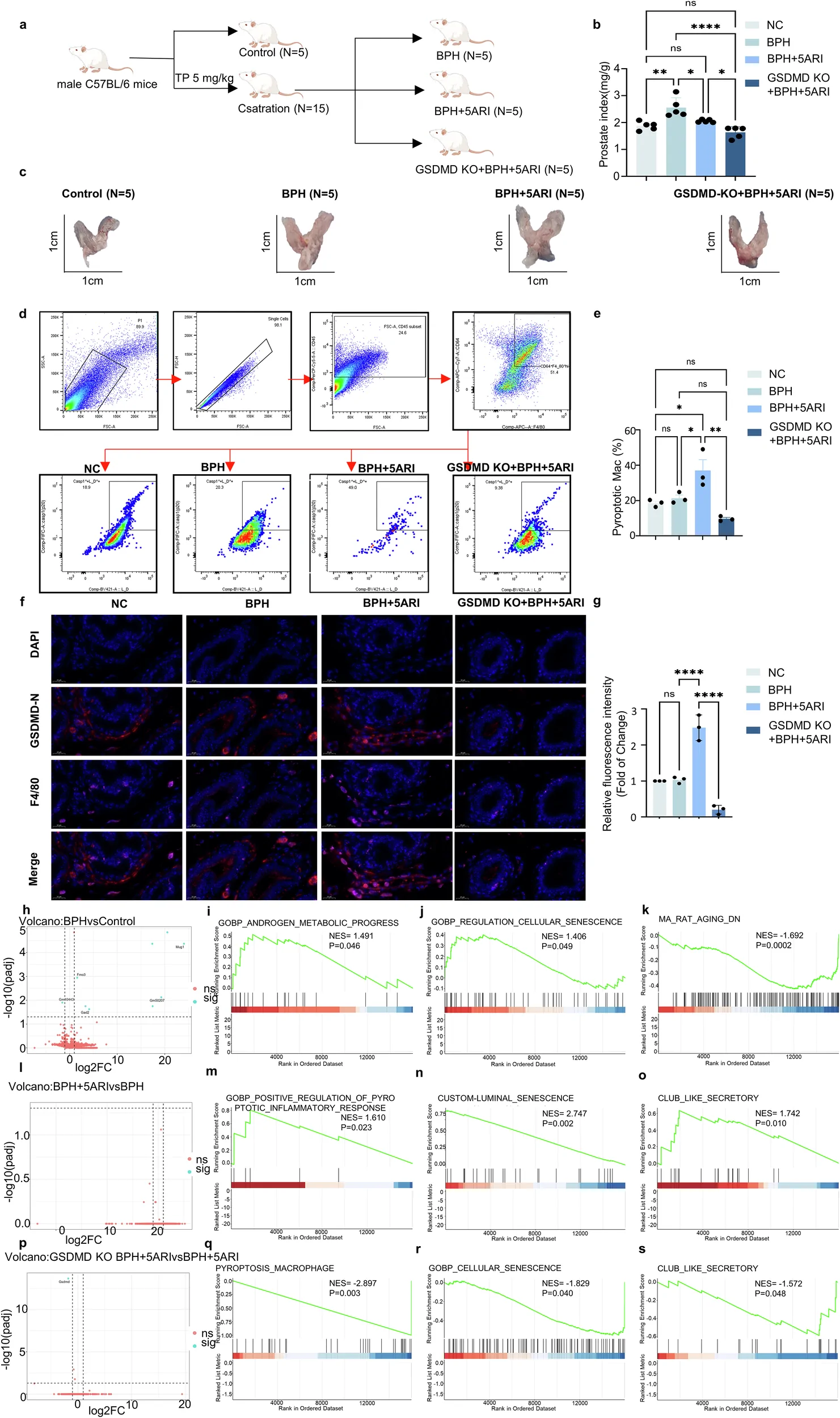
In vivo inhibition of pyroptosis mitigates prostate enlargement and depletes pyroptotic macrophages. **a** Experimental scheme. Male C57BL/6 mice were randomized into NC, BPH, BPH + 5-ARI, and Gsdmd-KO + BPH + 5-ARI groups. A castration/testosterone-induced BPH model was used to standardize the endogenous androgenic background before exogenous testosterone-driven BPH induction. Mice in the treatment arm received 5-ARI in this standardized BPH model background, and prostate and body weights were recorded at endpoint.** b**,**c** Prostate index, calculated as prostate weight/body weight. Individual values and group summaries are shown. The prostate index was elevated in BPH versus NC, reduced after 5-ARI treatment, and further decreased in the Gsdmd-deficient background. Group means ± s.e.m.; *n* = 5 per group: NC, 1.88 ± 0.07; BPH, 2.56 ± 0.16; BPH + 5-ARI, 2.06 ± 0.02; Gsdmd-KO + BPH + 5-ARI, 1.64 ± 0.09. **d** Flow cytometry gating. After exclusion of Zombie Violet-positive cells, tissue macrophages were defined as live CD45^+^F4/80^+^CD64^+^ cells, with active caspase-1 p20 marking pyroptosis-associated activation. **e** Quantification of pyroptotic macrophages. The pyroptotic macrophage fraction increased in BPH + 5-ARI compared with NC and BPH, and was markedly reduced by Gsdmd loss. Means ± s.e.m.; *n* = 3 per group: NC, 18.53 ± 1.12; BPH, 21.33 ± 1.85; BPH + 5-ARI, 37.00 ± 5.84; Gsdmd-KO + BPH + 5-ARI, 9.67 ± 0.82. **f** Representative multiplex IF images showing F4/80, GSDMD-N and DAPI, with periglandular enrichment of GSDMD-N^+^ macrophages in BPH + 5-ARI and attenuation in Gsdmd-KO + BPH + 5-ARI. Scale bar, 50 μm. **g** IF intensity quantification normalized to NC. BPH + 5-ARI showed increased F4/80/GSDMD-N signal, whereas Gsdmd-KO + BPH + 5-ARI showed reduced signal. **h** Volcano plot showing differential gene expression in BPH versus NC prostates. **i**–**k** GSEA of BPH versus NC prostates, showing positive enrichment of an androgen-metabolic program (**i**) and a cellular-senescence-regulatory program (**j**), together with negative enrichment of the MA_RAT_AGING_DN signature (**k**).** l** Volcano plot showing differential gene expression in BPH + 5-ARI versus BPH prostates. **m**–**o** GSEA of BPH + 5-ARI versus BPH prostates, showing positive enrichment of a pyroptotic inflammatory-response program (**m**), a custom luminal-senescence signature (**n**) and a club-like secretory signature (**o**). **p** Volcano plot showing differential gene expression in Gsdmd-KO + BPH + 5-ARI versus BPH + 5-ARI prostates. **q**–**s** GSEA of Gsdmd-KO + BPH + 5-ARI versus BPH + 5-ARI prostates, showing negative enrichment of the pyroptosis–macrophage signature (**q**), the cellular-senescence program (**r**) and the club-like secretory signature (**s**), consistent with attenuation of these programs after Gsdmd loss. For **h**, **l** and **p**, volcano plots display log_2_ fold change and −log_10_(adjusted *P*). For **i**–**k**, **m**–**o** and **q**–**s**, normalized enrichment scores (NES) and* P* values are shown in the corresponding panels. Bars show group means ± s.e.m.; points indicate individual animals. Multigroup comparisons were performed using the panel-specific procedures described in the ‘Materials and methods’ section. Exact* P* values are shown in the panels or provided in the Source Data file.

To test whether macrophage pyroptosis contributes causally to 5-ARI-associated tissue remodeling in vivo, we used a castration/testosterone-induced mouse BPH model (Fig. 7a). In this model, surgical castration was used to minimize endogenous androgen variability, followed by exogenous testosterone administration to induce reproducible prostatic hyperplasia, consistent with established testosterone-driven experimental BPH models^27–30^. This design is a standardized experimental BPH induction strategy and should not be interpreted as a clinical androgen-deprivation therapy model. After BPH induction, mice received vehicle or 5-ARI treatment, and Gsdmd-deficient mice were used to test pyroptosis dependency.

Across four mouse cohorts (NC, BPH, BPH + 5-ARI and Gsdmd-knockout (KO) + BPH + 5-ARI), the prostate index (prostate weight/body weight) changed stepwise with intervention: it increased in BPH relative to control, decreased after 5-ARI treatment, and declined further on a Gsdmd-deficient background (Fig. 7b,c). Group means (±s.e.m.; *n* = 5 per group) were NC 1.88 ± 0.07, BPH 2.56 ± 0.16, BPH + 5-ARI 2.06 ± 0.02 and Gsdmd-KO + BPH + 5-ARI 1.64 ± 0.09, indicating partial correction by 5-ARI treatment and an additional reduction when pyroptosis was suppressed.

Consistent with these volumetric changes, pyroptotic macrophages were enriched after 5-ARI exposure and markedly reduced by Gsdmd loss. By flow cytometry (CD45^+^F4/80^+^CD64^+^ macrophages; active caspase-1^+^), the BPH + 5-ARI group increased relative to both NC and BPH, whereas Gsdmd deficiency reduced this fraction to well below all other groups (Fig. 7d,e; mean % ± s.e.m.; *n* = 3 per group: NC 18.53 ± 1.12, BPH 21.33 ± 1.85, BPH + 5-ARI 37.00 ± 5.84 and Gsdmd-KO + BPH + 5-ARI 9.67 ± 0.82).

Multiplex IF (F4/80 × GSDMD-N) corroborated these findings: relative to NC, the normalized signal increased in BPH + 5-ARI and dropped with Gsdmd deletion (Fig. 7f,g; *n* = 3 per group), effectively reducing the pyroptosis-associated macrophage readout. In parallel, p16 IF and the club-marker Krt4 showed the same directionality—elevated in BPH + 5-ARI and reduced with Gsdmd loss (Supplementary Fig. 13a–d). Bulk RNA-seq provided complementary transcriptional support. Relative to NC, BPH prostates showed enrichment of androgen-metabolic and cellular-senescence-regulatory programs, together with negative enrichment of the MA_RAT_AGING_DN signature (Fig. 7h–k). Compared with BPH, BPH + 5-ARI prostates showed enrichment of pyroptotic inflammatory-response, luminal-senescence and club-like secretory programs (Fig. 7l–o). Conversely, Gsdmd loss in the BPH + 5-ARI background attenuated the pyroptosis–macrophage, cellular-senescence and club-like secretory signatures (Fig. 7p–s). Together, these morphological, cytometric and transcriptomic data indicate that 5-ARI exposure in a standardized castration/testosterone-induced BPH model promotes macrophage pyroptosis and is accompanied by senescence/club-like remodeling, whereas genetic suppression of pyroptosis through Gsdmd loss depletes this macrophage state and further alleviates prostate enlargement.

### Srd5a2-deficient mouse prostate scRNA-seq supports epithelial remodeling and immune–epithelial communication after perturbation of the 5α-reductase axis

To place the 5-ARI-associated epithelial remodeling program in an independent mouse single-cell context, we reanalyzed three public mouse prostate scRNA-seq datasets from GSE235514. According to the GEO sample annotation, this dataset contains one Srd5a2-KO sample (M2) and two Srd5a2-heterozygous samples (M4 and M6). Therefore, this public dataset was interpreted as an Srd5a2-deficiency-associated scRNA-seq model of perturbation of the 5α-reductase axis, rather than as a Gsdmd-deficient dataset.

**Fig. 8:**
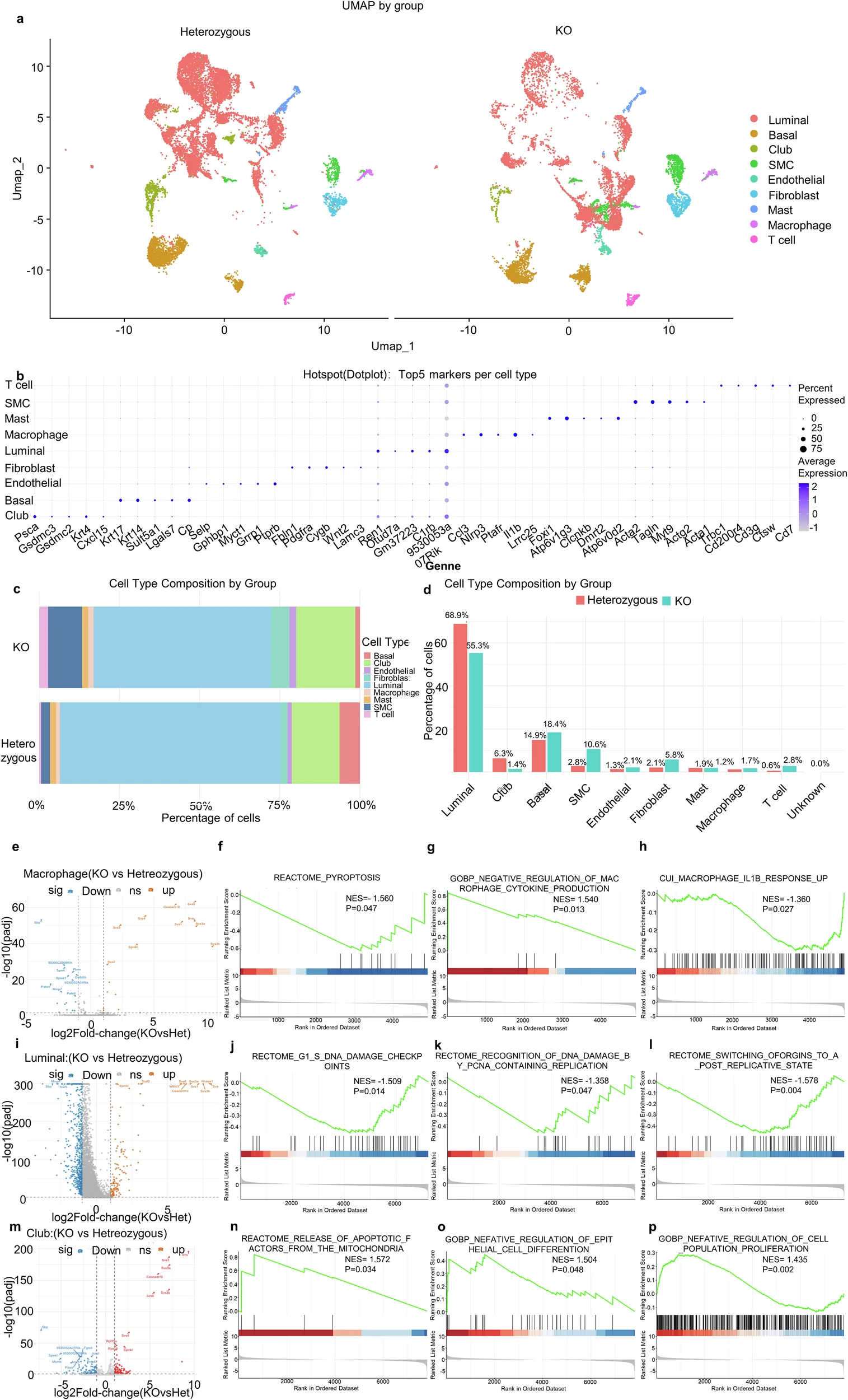
Srd5a2-deficient mouse prostate scRNA-seq reveals epithelial remodeling linked to perturbation of the 5α-reductase axis. **a** UMAP plots of integrated mouse prostate scRNA-seq data from GSE235514, split by genotype and colored by annotated cell type. The heterozygous group comprises Srd5a2+/− samples M4 and M6, and the knockout (KO) group comprises the Srd5a2−/− sample M2. **b** Dot plot showing the top five marker genes for each annotated cell type. Dot size indicates the percentage of cells expressing each gene, and color indicates average expression. **c** Stacked bar plots showing cell-type composition within each genotype group. **d** Grouped bar plots comparing cell-type percentages between the heterozygous and KO groups. **e** Volcano plot of differential gene expression in macrophages for the KO versus heterozygous comparison. **f**–**h** Gene set enrichment analysis (GSEA) plots in macrophages for pyroptosis (**f**), negative regulation of macrophage cytokine production (**g**), and the CUI_MACROPHAGE_IL1B_RESPONSE_UP gene set (**h**). **i** Volcano plot of differential gene expression in luminal epithelial cells for the KO versus heterozygous comparison. j -l GSEA plots in luminal epithelial cells for G1/S DNA-damage checkpoints (**j**), PCNA-associated recognition of DNA damage (**k**), and switching of replication origins to a post-replicative state (**l**). **m** Volcano plot of differential gene expression in club cells for the KO versus heterozygous comparison. **n**–**p** GSEA plots in club cells for release of apoptotic factors from mitochondria (**n**), negative regulation of epithelial cell differentiation (**o**), and negative regulation of cell population proliferation (**p**). These analyses are based on public GSE235514 scRNA-seq data and should be interpreted as exploratory, because the dataset contains Srd5a2-deficient rather than Gsdmd-deficient mouse prostates and includes limited biological replication. Gsdmd dependency was tested directly in the in vivo BPH + 5-ARI Gsdmd-deficient model shown in Fig. 7.

After unified quality control, normalization, integration, and clustering, Srd5a2^+/−^ and Srd5a2^−/−^ cells aligned in a shared UMAP space with clear lineage annotations (Fig. 8a,b). Genotype-stratified analysis suggested altered epithelial and stromal/immune composition in the Srd5a2-deficient prostate, including changes in luminal and club epithelial compartments and relative shifts in basal, stromal, endothelial, macrophage and T cell populations (Fig. 8c,d). To minimize parameter-driven bias, we assessed clustering stability across resolutions using clustree and selected an optimal solution of 42 clusters (Supplementary Fig. 13e). Clusters were annotated and consolidated into nine major cell classes with canonical-marker support (Supplementary Fig. 13f–h and Supplementary Figs. 14 and 15).

We next examined whether macrophage inflammatory/pyroptosis-related programs and luminal senescence-linked programs were detectable in this Srd5a2-deficiency-associated context. Macrophage pyroptosis/IL-1β-response signatures, luminal senescence/replication-stress and DNA-damage checkpoint programs, and club apoptosis-related gene sets were assessed across annotated cell types (Fig. 8e–p). These analyses were interpreted as exploratory and supportive because GSE235514 contains Srd5a2-deficient rather than Gsdmd-deficient mouse prostates and includes limited biological replication.

To explore immune–epithelial communication, we stratified macrophages by a pyroptosis module score (Pyro-High versus Pyro-Low) and luminal epithelial cells by a senescence module score (Senes-High versus Senes-Low). Score distributions, UMAP localization and associated differential-expression/pathway features for these strata are shown for both Srd5a2^+/−^ and Srd5a2^−/−^ cohorts (Supplementary Figs. 16a–p and 17a–p). In the Srd5a2^+/−^ cohort, Pyro-High macrophages formed denser and stronger ligand–receptor networks than Pyro-Low macrophages, and Senes-High luminal cells exhibited higher interaction counts/weights than Senes-Low cells (Supplementary Fig. 16q–z1). Pairwise comparisons showed stronger Mac_Pyro-High ↔ Luminal_Senes-High coupling than Mac_Pyro-Low ↔ Luminal_Senes-High, and stronger Luminal_Senes-High ↔ Club coupling than Luminal_Senes-Low ↔ Club (Supplementary Fig. 16z2–z5). Ranked pathways highlighted MIF–CD74 among the top signals from Pyro-High to Senes-High, accompanied by ECM/injury-linked programs, whereas the Senes-High ↔ Club interface was enriched for LAMININ/WNT/EGF signaling with a detectable NOTCH receptor axis (Supplementary Fig. 16z6–z7). The Srd5a2^−/−^ cohort showed a broadly similar communication topology, including the corresponding network views, pairwise comparisons and pathway rankings (Supplementary Fig. 17q–z7). Together, this public Srd5a2-deficiency-associated scRNA-seq reanalysis provides independent contextual support that perturbation of the 5α-reductase axis is associated with epithelial lineage plasticity and immune–epithelial communication. Gsdmd dependency, however, was tested directly in our castration/testosterone-induced BPH model with Gsdmd deletion under 5-ARI exposure (Fig. 7), rather than inferred from GSE235514.

## Discussion

This study examines response heterogeneity to 5-ARIs in BPH and proposes—then validates—a directional immune–epithelial cascade. Under 5-ARI pressure, epithelial MIF binds macrophage CD74 as an ‘ignition signal’, activating the NLRP3 inflammasome–caspase-1–GSDMD axis to trigger pyroptosis with release of IL-1β, IL-18 and HMGB1-type DAMPs. HMGB1 and its receptor AGER (RAGE) constitute the key immune–epithelial interface. In the 5-ARI-conditioned microenvironment, the HMGB1–AGER axis induces epithelial p16⁺ senescence and G1 arrest and, via SASP-mediated paracrine signaling, reprograms luminal cells toward an SCGB1A1⁺ club-like lineage, establishing a ‘low-proliferation, high-survival’ tolerance-like epithelial adaptation state. Paracrine plasticity is supported by mammary/breast cancer models in which IL-6/IL-8 reinforce inflammatory and tumorigenic cell states^31^, and by foundational SASP studies showing that senescent cells exert cell-nonautonomous effects through secretory programs^32^, indicating a transferable context for ‘senescence–paracrine–lineage reprogramming’.

First, we shift the pyroptosis–senescence link from correlation to directionality: genetic, blockade–rescue and multi-omic data place GSDMD-dependent macrophage pyroptosis upstream, with HMGB1 as the messenger, and epithelial senescence/lineage drift downstream. Cross-tissue evidence supports the generality of ‘pyroptosis → remodeling/senescence’: in trauma–heterotopic ossification, pyroptotic macrophages induce stem-cell senescence via IL-1β and HMGB1-bearing vesicles; blocking pyroptosis or removing HMGB1 reverses the phenotype^33^. In diabetic periodontitis, CM from pyroptotic macrophages induces senescence in human gingival fibroblasts, while clearing pyroptotic macrophages restores rejuvenation indices^34^.

Second, we decouple ‘infiltration’ from ‘activation’. Immune-cell infiltration—especially macrophages—recurs in BPH^35–38^. Chemotactic axes such as CXCL12–CXCR4 recruit and enrich monocytes/macrophages across inflammatory and tumor contexts^39–41^, and HMGB1 complexes with CXCL12 to enhance migration via CXCR4^42^. Yet, infiltration alone is insufficient; specific binding of epithelial MIF to macrophage CD74 is the upstream trigger converting residents to effectors. This pro-inflammatory/immunoregulatory axis correlates with adverse biology across diseases: high MIF–CD74 activation links to invasive phenotype and poor prognosis in ovarian cancer; in melanoma/brain metastasis and lung-cancer models, MIF–CD74 inhibition improves antitumor responses or radiosensitivity, demonstrating druggability^43–45^. Together with our causal BPH data, these findings nominate MIF–CD74 as the 5-ARI ‘ignition switch’.

Third, we redefine HMGB1–AGER in the 5-ARI context. Beyond associations with treatment tolerance and immune evasion^46,47^, our data suggest that this axis contributes to a tolerance-like epithelial adaptation state characterized by ‘cell-cycle restraint plus high survival’—by inducing epithelial p16⁺ senescence and G1 arrest. Senescence is not terminal; SASP-driven signaling pushes adjacent luminal epithelium toward an SCGB1A1⁺ club-like lineage, raising the apoptotic threshold. In parallel, Notch signaling governs basal-cell differentiation into club/goblet/ciliated lineages during airway development and disease, and Notch inhibition limits goblet-cell mucous metaplasia^48^. ECM mechanics and EGFR/WNT pathways also shape epithelial plasticity and lineage stability^49–51^. Single-cell studies of human prostate and urethra identify SCGB1A1⁺ club-like epithelium and its enrichment or association with inflammation/castration resistance in PIA/BPH/tumor settings; spatial-transcriptomic data in BPH likewise indicate 5-ARI-associated luminal-to-club-like transitions^52–55^, collectively supporting stabilization and amplification of the proposed ‘senescence → club-like reprogramming’ trajectory.

This framework extends explanations of 5-ARI response heterogeneity from a singular endocrine–apoptosis view to a programmatic immune–epithelial interaction. Once MIF–CD74 ignition triggers pyroptosis and HMGB1 release, AGER-driven epithelial cells enter a ‘senescence–reprogramming’ path; tissues shift from a ‘high-proliferation, apoptosis-prone’ state to ‘low-proliferation, high-survival’, attenuating 5-ARI gland-shrinking effects. Clinical evidence and guidelines show that 5-ARIs improve symptoms and reduce risks of acute urinary retention and surgery^3,8–10^, yet many patients still progress on long-term therapy, consistent with ‘immune–epithelial programmatic adaptation’.

Translationally, we operationalize a 5-ARI-associated tolerance-like immune–epithelial state as a measurable tissue program and propose a predictive PM-13 score for early stratification and dynamic monitoring (blood/urine HMGB1 is detectable)^56,57^. Guided by ‘macrophage pyroptosis → HMGB1–AGER → epithelial senescence/club’, we advocate sequential intervention—‘upstream first, downstream next’: de-ignite (target MIF/CD74), dampen DAMPs (block HMGB1/AGER) or inhibit GSDMD, then reshape (for example, moderated Notch tuning). Window-of-opportunity/neoadjuvant short-course designs using tissue pharmacodynamics as the primary endpoint are suitable; breast-tumor studies show short courses can yield interpretable tissue signals within ~2 weeks for stratification and prognosis^58^.

Limitations include spot mixing in ST; DAMP redundancy, including ATP and S100-family mediators; the need for lineage tracing to define pyroptosis specificity; and the partial reversal of the club phenotype after Notch inhibition, which implies multipathway cooperation and/or matrix locking. A further limitation is that the mouse model used surgical castration followed by exogenous testosterone administration to standardize the androgenic background before BPH induction, rather than modeling clinical 5-ARI monotherapy directly. Mice were then exposed to 5-ARI treatment in this standardized BPH background. This design may amplify reduced-androgen remodeling relative to routine human 5-ARI monotherapy; therefore, the mouse data should be interpreted as causal support for the pyroptosis–senescence–club axis under a controlled androgen-standardized BPH condition, rather than as a direct phenocopy of human 5-ARI monotherapy. We acknowledge that the pseudotime-defined epithelial branches in the ST cohort were inferred from cross-sectional tissue states and were not directly linked to matched longitudinal clinical endpoints, such as prostate volume reduction, symptom improvement, urinary-flow improvement or progression to surgery. Therefore, we now interpret these branches as 5-ARI-associated adaptive transcriptional states rather than definitive clinical response categories. Clinical response stratification is addressed separately through the independent GSE262946 cohort used for PM-13 validation, rather than being directly assigned to the ST-derived branches.

In sum, our data support a systems-level immune–epithelial model in which MIF–CD74-associated macrophage activation/pyroptosis functions upstream of HMGB1–AGER-mediated epithelial senescence, while epithelial p16⁺ senescence and partially Notch-associated remodeling favor SCGB1A1⁺ club-like drift. This framework provides a mechanistic basis for a tolerance-like ‘low-proliferation, high-survival’ epithelial state, offers actionable paths for precision stratification and sequential combination strategies, and points to pharmacodynamic endpoints testable in window-of-opportunity designs.

## Supplementary information

Supplementary Information

Supplementary Table 1

Supplementary Table 2

Source Data

## Data Availability

For spatial/histologic sections, we integrated GSE179615 and GSE278936, yielding 14 sections in total (normal, *n* = 2; BPH without medication, *n *= 6; BPH after 5-ARI, *n *= 6). These ST datasets provide tissue and treatment-status annotations but do not include matched longitudinal clinical response endpoints. We further analyzed the longitudinal cohort GSE262946 with paired time points—baseline (Fin-BL) and follow-up (Fin-FU)—and labeled each case as responsive or resistant according to the clinical outcomes reported in the original study. For mouse scRNA-seq reanalysis, we reprocessed three public prostate scRNA-seq samples from GSE235514: M2 (Srd5a2^−/−^) and M4/M6 (Srd5a2^+/−^), according to the GEO sample annotations. This dataset was used as an independent Srd5a2-deficiency-associated scRNA-seq context for assessing epithelial remodeling and immune–epithelial communication after perturbation of the 5α-reductase axis.
