# Supplementary material for: Macrophage pyroptosis drives luminal senescence and club-cell reprogramming in 5α-reductase inhibitor-treated prostatic hyperplasia
Source: Exp Mol Med. 2026 Oct 7;58(10):3514–33. doi: 10.1038/s12276-026-01864-3 (PMC13737177; doi:10.1038/s12276-026-01864-3)
Supplement: Supplementary file 1 [file 12276_2026_1864_MOESM1_ESM.pdf]

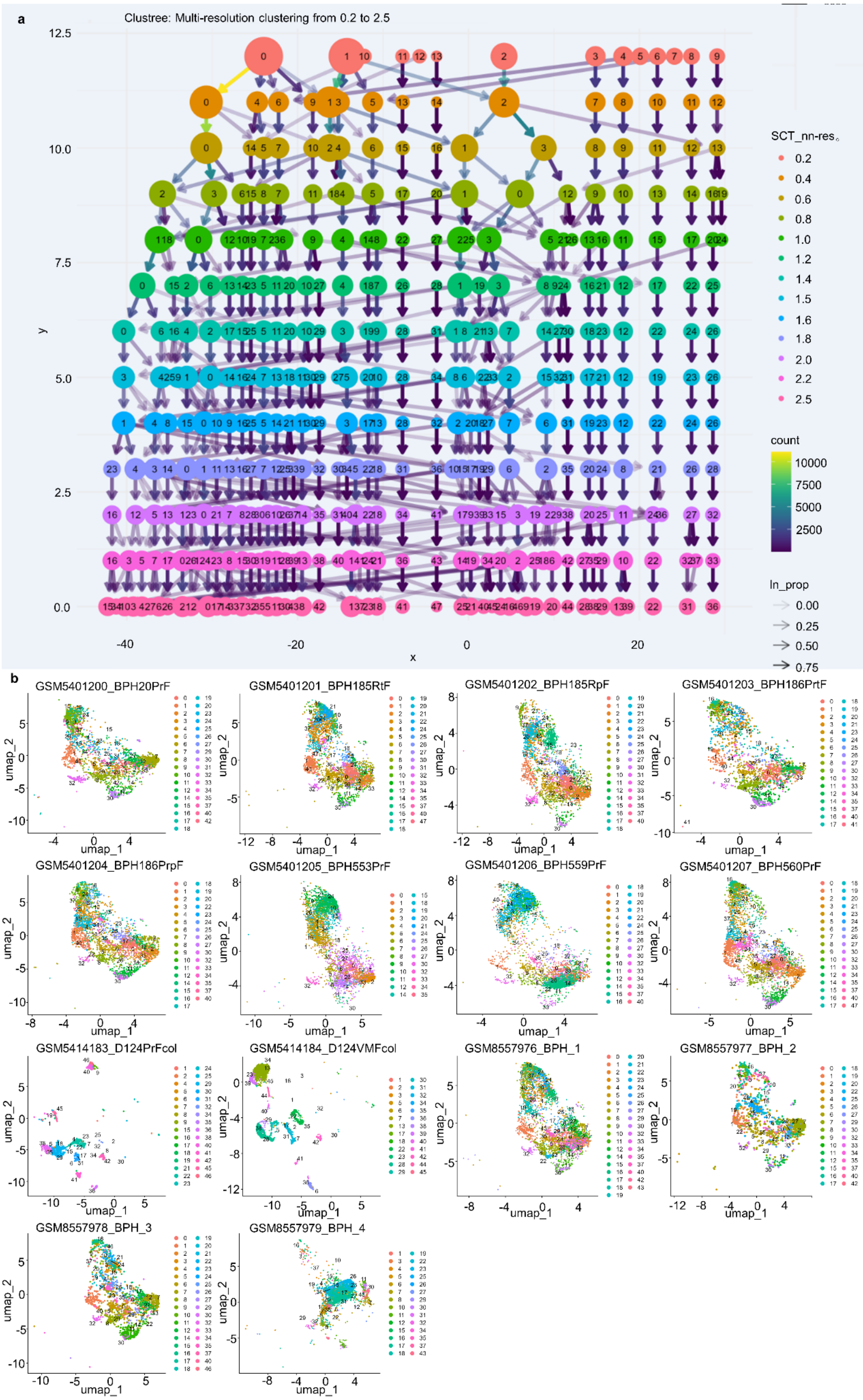
a
Clustree: Multi-resolution clustering from 0.2 to 2.5
SCT_nn-res.
0.2
0.4
0.6
0.8
1.0
1.2
1.4
1.5
1.6
1.8
2.0
2.2
2.5
count
10000
7500
5000
2500
in_prop
0.00
0.25
0.50
0.75
x
y
b
GSM5401200_BPH20PrF
GSM5401201_BPH185RtF
GSM5401202_BPH185RpF
GSM5401203_BPH186PrtF
GSM5401204_BPH186PrpF
GSM5401205_BPH553PrF
GSM5401206_BPH559PrF
GSM5401207_BPH560PrF
GSM5414183_D124PrFcol
GSM5414184_D124VMFcol
GSM8557976_BPH_1
GSM8557977_BPH_2
GSM8557978_BPH_3
GSM8557979_BPH_4
umap_1
umap_2

Supplementary Fig. 1 | Multi-resolution clustering stability and per-sample embeddings

a, clustree visualization of cluster splitting and inheritance across resolutions (Seurat/Leiden, resolution 0.2–2.5). Each node denotes a cluster at a given resolution, with node size scaled by the number of constituent spots and node color encoding resolution. Edges trace inheritance from lower- to higher-resolution "parent→child" clusters; edge thickness/opacity reflects the proportion of inherited membership. This panel assesses topological stability across resolutions and informs the choice of an optimal resolution.

b, UMAP projections of 14 ST sections (normal, n = 2; untreated BPH, n = 6; BPH after 5-ARI, n = 6; total 46,176 spots), colored by cluster identity at the selected optimal resolution. Per-sample embeddings recapitulate the cluster architecture and relative abundances observed in the merged analysis, supporting sample-level consistency and effective batch correction.

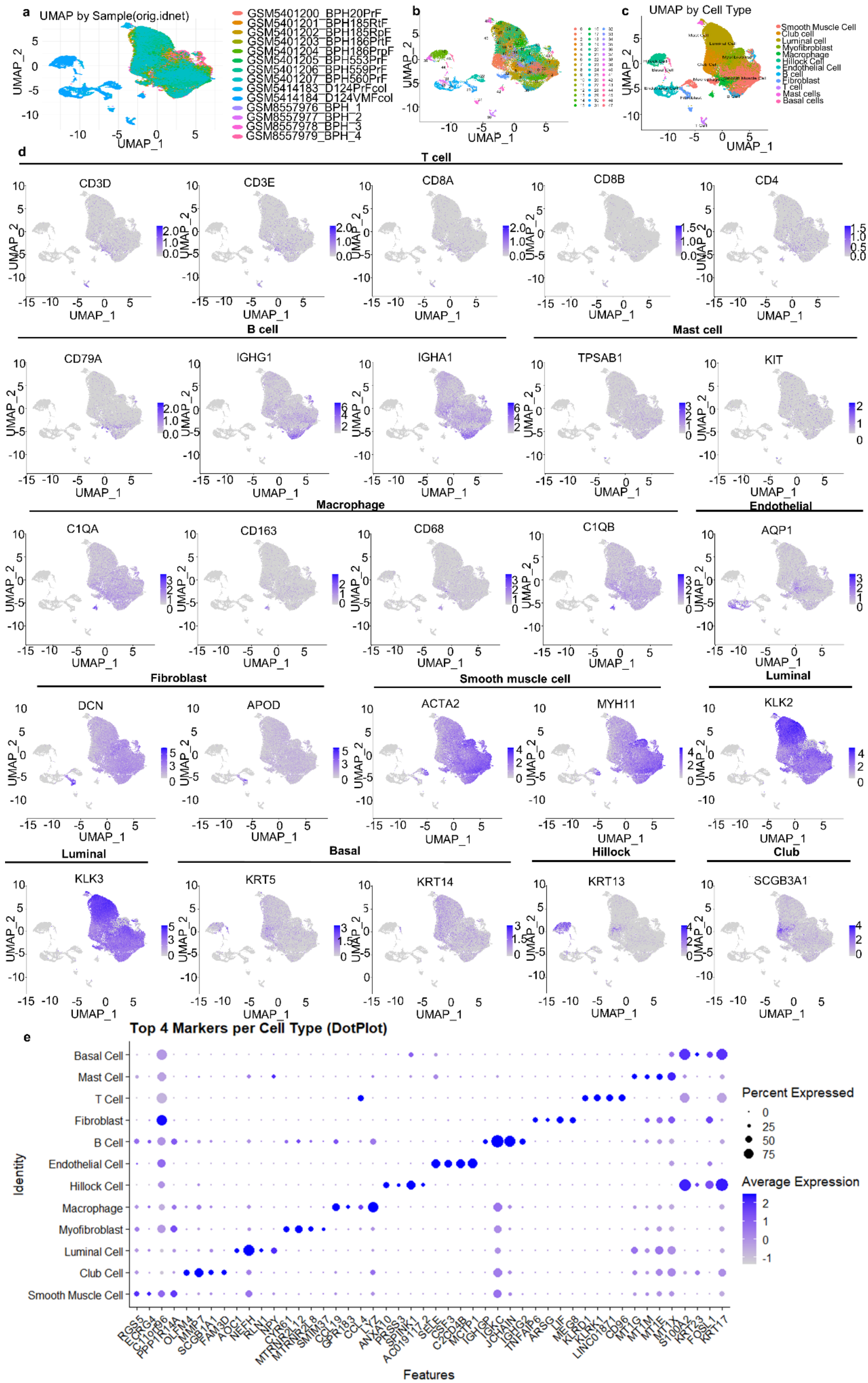

Supplementary Fig. 2 | Cell-type annotation and marker validation

a, UMAP of the merged 14 ST sections (46,176 spots in total) in a shared coordinate space, colored by sample identity (orig.ident) to illustrate post-integration mixing and global topology.

b, The same UMAP colored by cluster identity, showing the distribution of the 48 clusters (0–47) obtained at the selected resolution.

c, The same UMAP colored by annotated cell type, showing the distribution and separability of 12 major cell classes in low-dimensional space.

d, FeaturePlots of representative marker genes; color denotes normalized expression from low to high. Examples include T cells (CD3D, CD3E, CD8B, CD4), B cells (CD79A, MS4A1, IGHA1), mast cells (TPSAB1, KIT), macrophages (C1QA, CD163, CD68, C1QB), endothelial cells (AQP1), fibroblasts (DCN, APOD), smooth muscle cells (ACTA2, MYH11), luminal cells (KLK2, KLK3), basal cells (KRT5), hillock cells (KRT13), and club cells (SCGB3A1).

e, DotPlot of the top four marker genes per cell type. Dot size indicates the fraction of spots expressing each gene, and color intensity indicates average expression within the corresponding cell type.

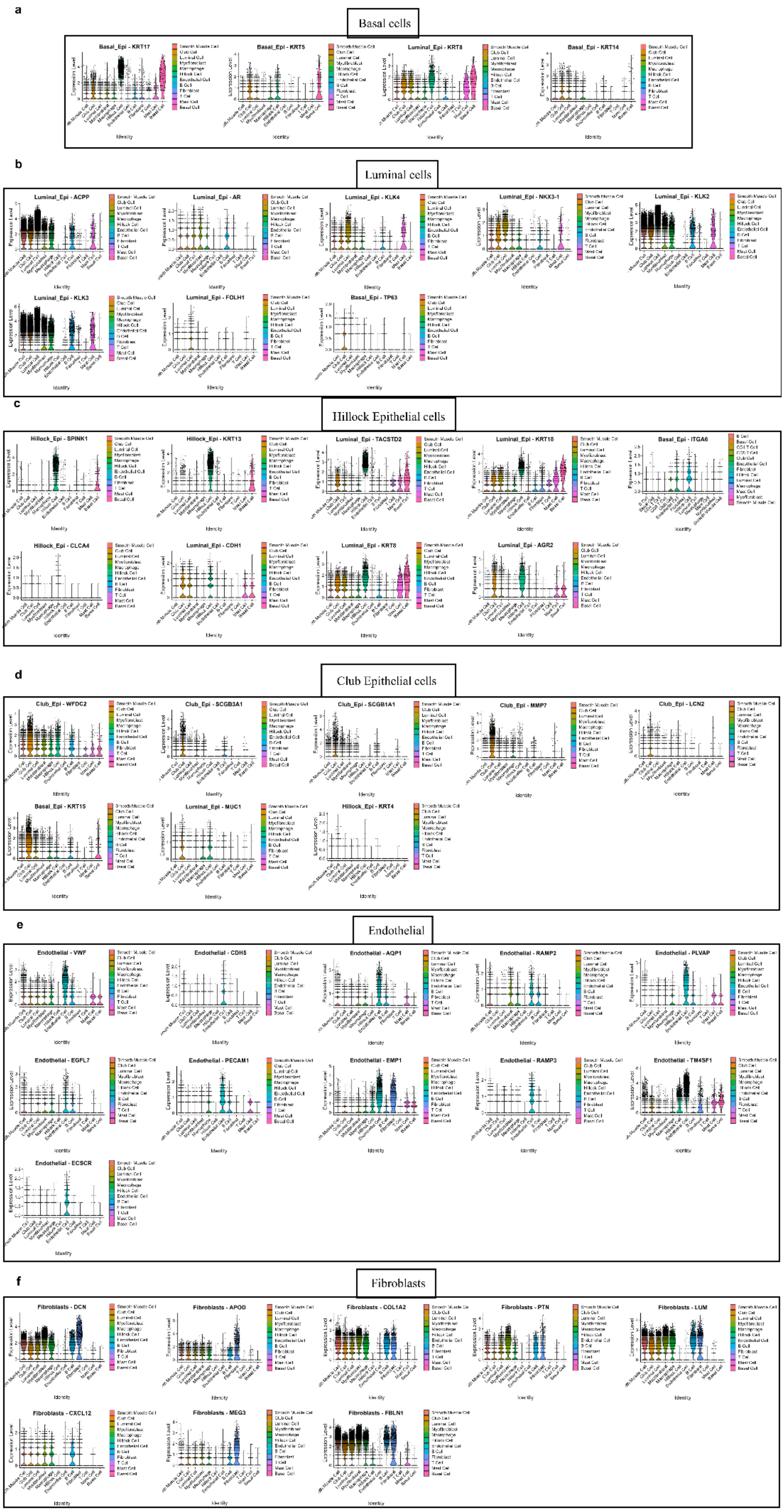
a
Basal cells
Basal_Epi - KRT17
Basal_Epi - KRT5
Luminal_Epi - KRT8
Basal_Epi - KRT14
b
Luminal cells
Luminal_Epi - ACPP
Luminal_Epi - AR
Luminal_Epi - KLK4
Luminal_Epi - NKX3-1
Luminal_Epi - KLK2
Luminal_Epi - KLK3
Luminal_Epi - FOLH1
Basal_Epi - TP63
c
Hillock Epithelial cells
Hillock_Epi - SPINK1
Hillock_Epi - KRT13
Luminal_Epi - TACSTD2
Luminal_Epi - KRT18
Basal_Epi - ITGA6
Hillock_Epi - CLCA4
Luminal_Epi - CDH1
Luminal_Epi - KRT8
Luminal_Epi - AGR2
d
Club Epithelial cells
Club_Epi - WFDC2
Club_Epi - SCGB3A1
Club_Epi - SCGB1A1
Club_Epi - MMP7
Club_Epi - LCN2
Basal_Epi - KRT15
Luminal_Epi - MUC1
Hillock_Epi - KRT4
e
Endothelial
Endothelial - VWF
Endothelial - CDH5
Endothelial - AQP1
Endothelial - RAMP2
Endothelial - PLVAP
Endothelial - EGFL7
Endothelial - PECAM1
Endothelial - EMP1
Endothelial - RAMP3
Endothelial - TM4SF1
Endothelial - ECSCR
f
Fibroblasts
Fibroblasts - DCN
Fibroblasts - APOD
Fibroblasts - COL1A2
Fibroblasts - PTN
Fibroblasts - LUM
Fibroblasts - CXCL12
Fibroblasts - MEG3
Fibroblasts - FBLN1

Supplementary Fig. 3 | Marker expression across cell subtypes (violin plots)

a Basal cells: canonical basal markers are enriched in the Basal cluster (KRT17, KRT5, KRT14), contrasting with epithelial keratins such as KRT8 that predominate in non-basal populations.

b Luminal cells: luminal markers are highly expressed in the Luminal cluster (ACP3, AR, KLK4, NKX3-1, KLK3, FOLH1), with TP63 serving as a negative/low marker, indicating separation from the basal lineage.

c Hillock epithelial cells: hillock-associated genes are enriched in the Hillock cluster (SPINK1, KRT13, TACSTD2, AGR2, CLCA4), distinguishing it from other epithelial subtypes.

d Club epithelial cells: club-related secretory markers show specific enrichment (WFDC2, SCGB3A1, SCGB1A1, MMP7, LCN2), supporting the club annotation.

e Endothelial: endothelial genes aggregate within the Endothelial cluster (VWF, PECAM1, CDH5, AQP1, RAMP2, PLVAP, EMP1) and are low in non-endothelial clusters.

f Fibroblasts: fibroblast markers are enriched in the Fibroblast cluster (DCN, APOD, COL1A2, PTN, LUM, CXCL12, MEG3, FBLN1), clearly separating it from other stromal/epithelial populations.

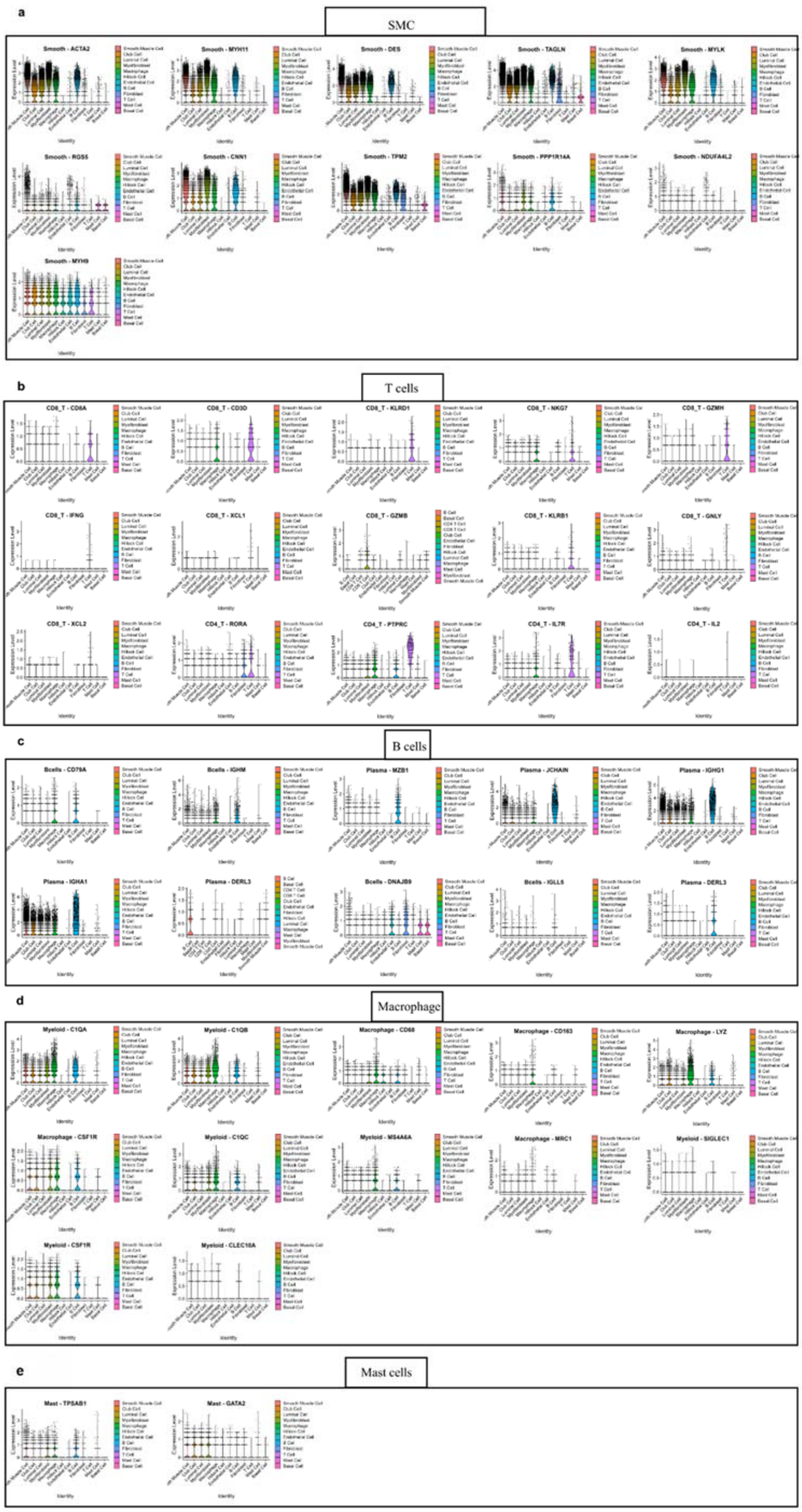
a
SMC
Smooth - ACTA2
Smooth - MYH11
Smooth - DES
Smooth - TAGLN
Smooth - MYLK
Smooth - RGS5
Smooth - CNN1
Smooth - TPM2
Smooth - PPP1R14A
Smooth - NDUFA4L2
Smooth - MYH9
b
T cells
CD8_T - CD8A
CD8_T - CD3D
CD8_T - KLRD1
CD8_T - NKG7
CD8_T - GZMH
CD8_T - IFNG
CD8_T - XCL1
CD8_T - GZMB
CD8_T - KLRB1
CD8_T - GNLY
CD8_T - XCL2
CD4_T - RORA
CD4_T - PTPRC
CD4_T - IL7R
CD4_T - IL2
c
B cells
Bcells - CD79A
Bcells - IGHM
Plasma - MZB1
Plasma - JCHAIN
Plasma - IGHG1
Plasma - IGHA1
Plasma - DERL3
Bcells - DNAJB9
Bcells - IGLL5
Plasma - DERL3
d
Macrophage
Myeloid - C1QA
Myeloid - C1QB
Macrophage - CD68
Macrophage - CD163
Macrophage - LYZ
Macrophage - CSF1R
Myeloid - C1QC
Myeloid - MS4A6A
Macrophage - MRC1
Myeloid - SIGLEC1
Myeloid - CSF1R
Myeloid - CLEC10A
e
Mast cells
Mast - TPSAB1
Mast - GATA2

Supplementary Fig. 4 | Marker expression in stromal smooth muscle and immune subpopulations (violin plots)

a SMC (smooth muscle cells): contractile markers are specifically enriched in SMCs (ACTA2, MYH11, DES, TAGLN, MYLK), together with contractile/actin apparatus genes RGS5, CNN1, and TPM2. The regulatory subunit PPP1R14A and metabolic/hypoxia-related NDUFA4L2, as well as non-muscle myosin MYH9, are selectively elevated in SMCs and low elsewhere.

b T cells: T-lineage and cytotoxicity-associated genes are enriched in the T-cell cluster (CD3D, CD8A, KLRD1, NKG7, GZMB, GNLY); effector cytokines/chemokines (IFNG, IL2, XCL1/XCL2) and memory marker IL7R are increased. PTPRC (CD45) and RORA serve as auxiliary markers, clearly separating T cells from other populations.

c B cells / Plasma: B-cell and plasma-cell markers cluster together (CD79A, IGHM), with upregulation of plasma-associated MZB1 and JCHAIN and immunoglobulin heavy chains IGHG1 and IGHA1; ER folding/stress chaperones DERL3 and DNAJB9 and the early B-cell marker IGLL5 are also elevated.

d Macrophage / Myeloid: myeloid/macrophage genes are enriched (complement C1QA/C1QB/C1QC; phagolysosomal markers CD68, LYZ; receptor CSF1R), together with tissue-macrophage markers MS4A4A and MRC1 (CD206).

e Mast cells: mast-cell–specific markers are upregulated (granule protease TPSAB1 and transcription factor GATA2), commonly accompanied by KIT expression.

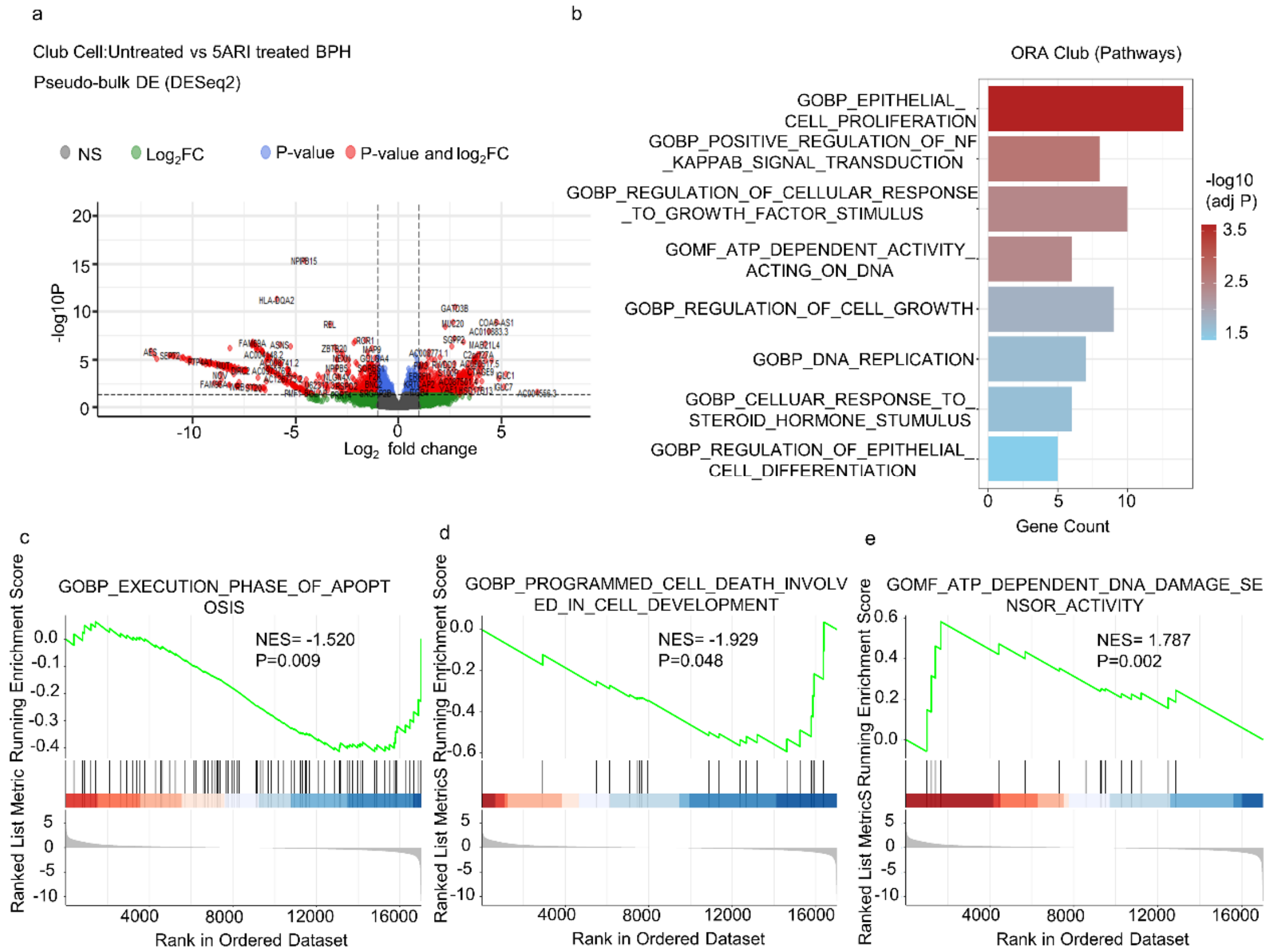

Supplementary Fig. 5 | Club epithelial pseudobulk and pathway analyses after 5-ARI treatment.

a, Donor-level pseudobulk differential-expression analysis of club regions comparing 5-ARI-treated BPH with untreated BPH. Differentially expressed genes are shown by log2 fold change and adjusted P value.

b, Over-representation analysis (ORA) of differentially expressed genes in club regions after 5-ARI treatment.

c–e, Representative preranked GSEA plots showing reduced apoptotic execution and enhanced DNA-damage sensing/repair programs in club regions after 5-ARI treatment.

Statistics: Pseudobulk differential-expression analysis was performed using donor-level aggregation and DESeq2 with Benjamini–Hochberg FDR correction. Pathway enrichment was assessed by ORA and preranked GSEA. FDR-adjusted q values,

normalized enrichment scores, and nominal P values are shown in the corresponding panels where applicable.

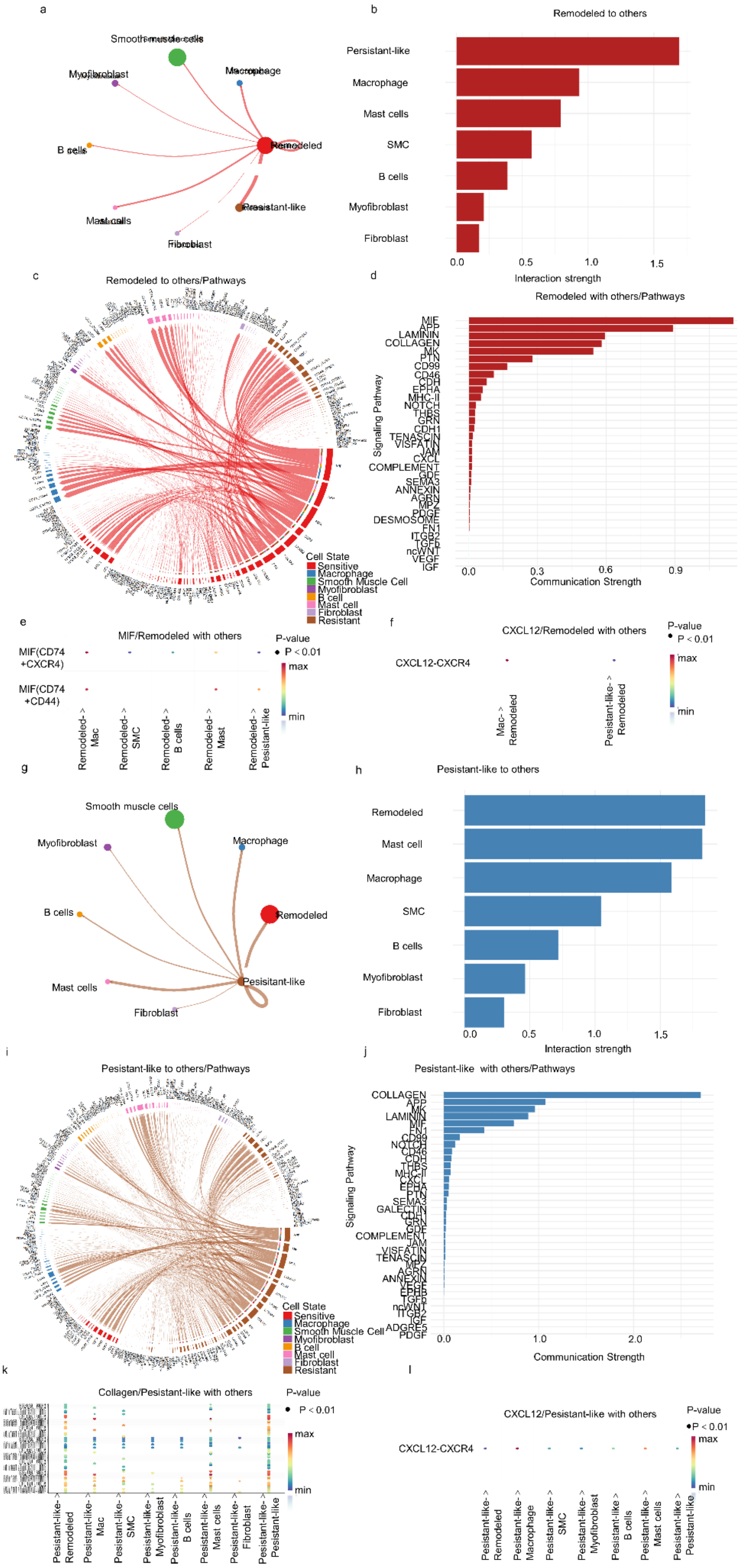

a
Smooth muscle cells
Myofibroblast
Macrophage
B cells
Remodeled
Mast cells
Resistant-like
Fibroblast
b
Remodeled to others
Persistant-like
Macrophage
Mast cells
SMC
B cells
Myofibroblast
Fibroblast
0.0 0.5 1.0 1.5
Interaction strength
c
Remodeled to others/Pathways
Cell State
Sensitive
Macrophage
Smooth Muscle Cell
Myofibroblast
B cell
Mast cell
Fibroblast
Resistant
d
Remodeled with others/Pathways
Signaling Pathway
MIF
APP
LAMININ
COLLAGEN
MK
PTN
CD99
CD46
CDH
EPHA
MHC-II
NOTCH
THBS
GRN
CDH1
TENASCIN
VISFATIN
JAM
CXCL
COMPLEMENT
GDF
SEMA3
ANNEXIN
AGRN
MPZ
PDGF
DESMOSOME
FN1
ITGB2
TGFb
ncWNT
VEGF
IGF
0.0 0.3 0.6 0.9
Communication Strength
e
MIF/Remodeled with others
MIF(CD74 +CXCR4)
MIF(CD74 +CD44)
Remodeled-> Mac
Remodeled-> SMC
Remodeled-> B cells
Remodeled-> Mast
Remodeled-> Pesistant-like
P-value
P < 0.01
max
min
f
CXCL12/Remodeled with others
CXCL12-CXCR4
Mac-> Remodeled
Pesistant-like-> Remodeled
P-value
P < 0.01
max
min
g
Smooth muscle cells
Myofibroblast
Macrophage
B cells
Remodeled
Mast cells
Pesisitant-like
Fibroblast
h
Pesistant-like to others
Remodeled
Mast cell
Macrophage
SMC
B cells
Myofibroblast
Fibroblast
0.0 0.5 1.0 1.5
Interaction strength
i
Pesistant-like to others/Pathways
Cell State
Sensitive
Macrophage
Smooth Muscle Cell
Myofibroblast
B cell
Mast cell
Fibroblast
Resistant
j
Pesistant-like with others/Pathways
Signaling Pathway
COLLAGEN
APP
MK
LAMININ
MIF
FN1
CD99
NOTCH
CD46
CDH
THBS
MHC-II
CXCL
EPHA
PTN
SEMA3
GALECTIN
CDH1
GRN
GDF
COMPLEMENT
JAM
VISFATIN
TENASCIN
MPZ
AGRN
ANNEXIN
VEGF
EPHB
TGFb
ncWNT
ITGB2
IGF
ADGRE5
PDGF
0.0 1.0 2.0
Communication Strength
k
Collagen/Pesistant-like with others
P-value
P < 0.01
max
min
Pesistant-like-> Remodeled
Pesistant-like-> Mac
Pesistant-like-> SMC
Pesistant-like-> Myofibroblast
Pesistant-like-> B cells
Pesistant-like-> Mast cells
Pesistant-like-> Fibroblast
Pesistant-like-> Pesistant-like
l
CXCL12/Pesistant-like with others
CXCL12-CXCR4
P-value
P < 0.01
max
min
Pesistant-like-> Remodeled
Pesistant-like-> Macrophage
Pesistant-like-> SMC
Pesistant-like-> Myofibroblast
Pesistant-like-> B cells
Pesistant-like-> Mast cells
Pesistant-like-> Pesistant-like

Supplementary Fig. 6 | Extended branch-to-other communication analyses of the 5-ARI-remodeled and persistent/tolerant-like epithelial states.

a, Network view showing outgoing communication from the 5-ARI-remodeled branch to other cell states and stromal/immune compartments.

b, Ranked interaction strength of outgoing signals from the 5-ARI-remodeled branch to other cell populations.

c, Chord diagram summarizing the pathway-level outgoing communication from the 5-ARI-remodeled branch to other cell states.

d, Ranked signaling pathways associated with outgoing communication from the 5-ARI-remodeled branch. Prominent pathways include MIF, LAMININ, COLLAGEN, and related signaling programs.

e, Bubble plot highlighting MIF-associated ligand–receptor interactions involving the 5-ARI-remodeled branch and other cell populations.

f, Bubble plot highlighting CXCL12-associated ligand–receptor interactions involving the 5-ARI-remodeled branch and other cell populations.

g, Network view showing outgoing communication from the persistent/tolerant-like branch to other cell states and stromal/immune compartments.

h, Ranked interaction strength of outgoing signals from the persistent/tolerant-like branch to other cell populations.

i, Chord diagram summarizing the pathway-level outgoing communication from the persistent/tolerant-like branch to other cell states.

j, Ranked signaling pathways associated with outgoing communication from the persistent/tolerant-like branch. Prominent pathways include COLLAGEN, APP, LAMININ, FN1, and related signaling programs.

k, Bubble plot highlighting collagen-associated ligand–receptor interactions involving the persistent/tolerant-like branch and other cell populations.

l, Bubble plot highlighting CXCL12-associated ligand–receptor interactions involving the persistent/tolerant-like branch and other cell populations.

Statistics: Communication analyses were performed using the spatially informed ligand–receptor inference framework described in Methods. Interaction strength and pathway ranking are shown in the corresponding panels. Bubble plots display selected significant ligand–receptor interactions, with color indicating relative communication strength and dot size or annotation indicating statistical significance ($P < 0.01$ where shown).

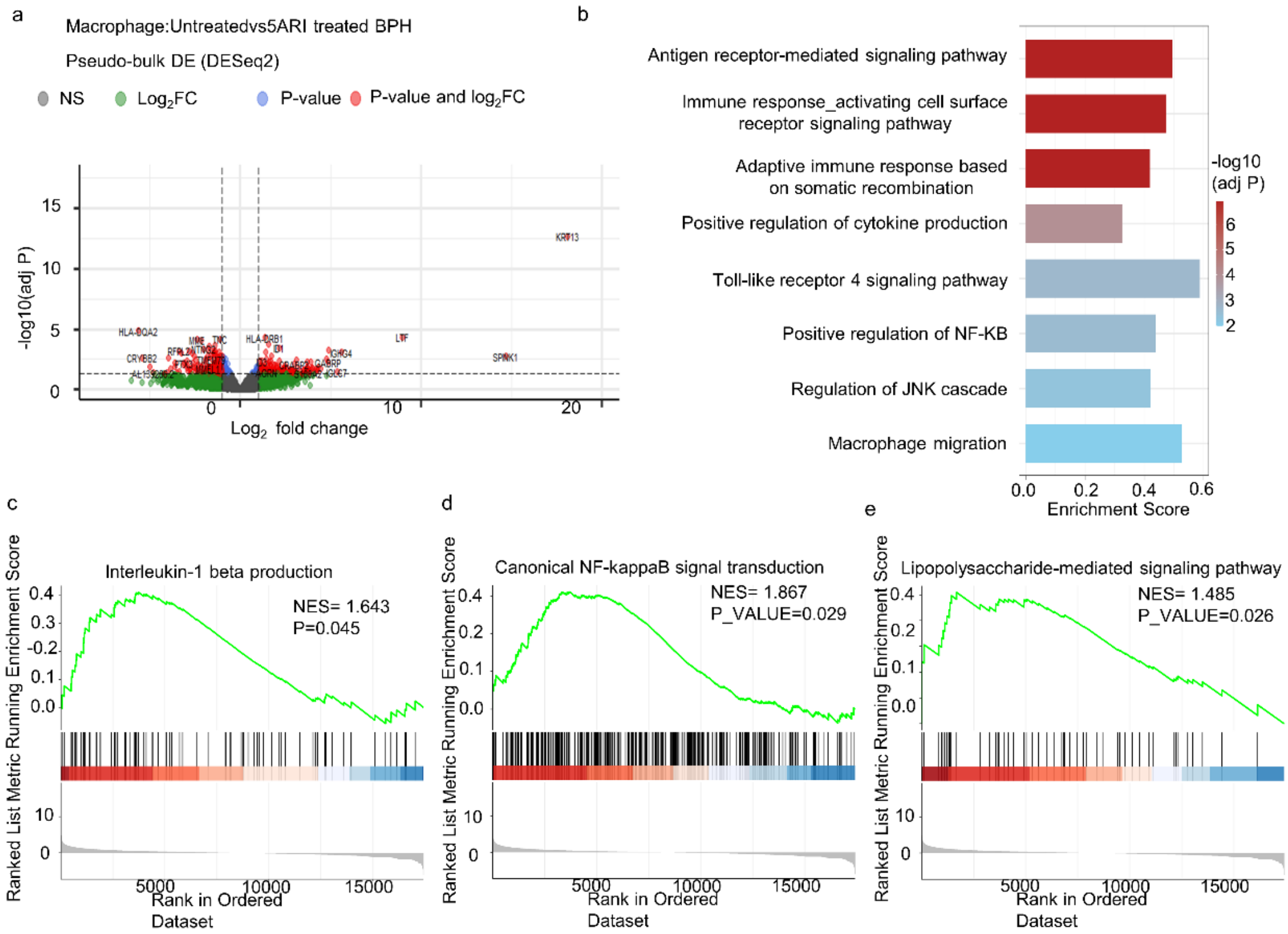

Supplementary Fig. 7 | Macrophage-side inflammatory pathway analyses after 5-ARI treatment.

a, Differential-expression analysis of macrophage-side programs associated with post–5-ARI epithelial–macrophage remodeling.

b, Pathway enrichment summary showing activation of inflammatory and innate immune response programs.

c–e, Representative preranked GSEA plots showing activation of IL-1β production, canonical NF-κB signaling, and LPS-like inflammatory cascades.

Statistics: Differential-expression analysis was performed using donor-level or cluster-level summaries as described in Methods. Pathway enrichment was assessed by ORA and preranked GSEA with multiple-testing correction. FDR-adjusted q values, normalized enrichment scores, and nominal P values are shown in the corresponding panels where applicable.

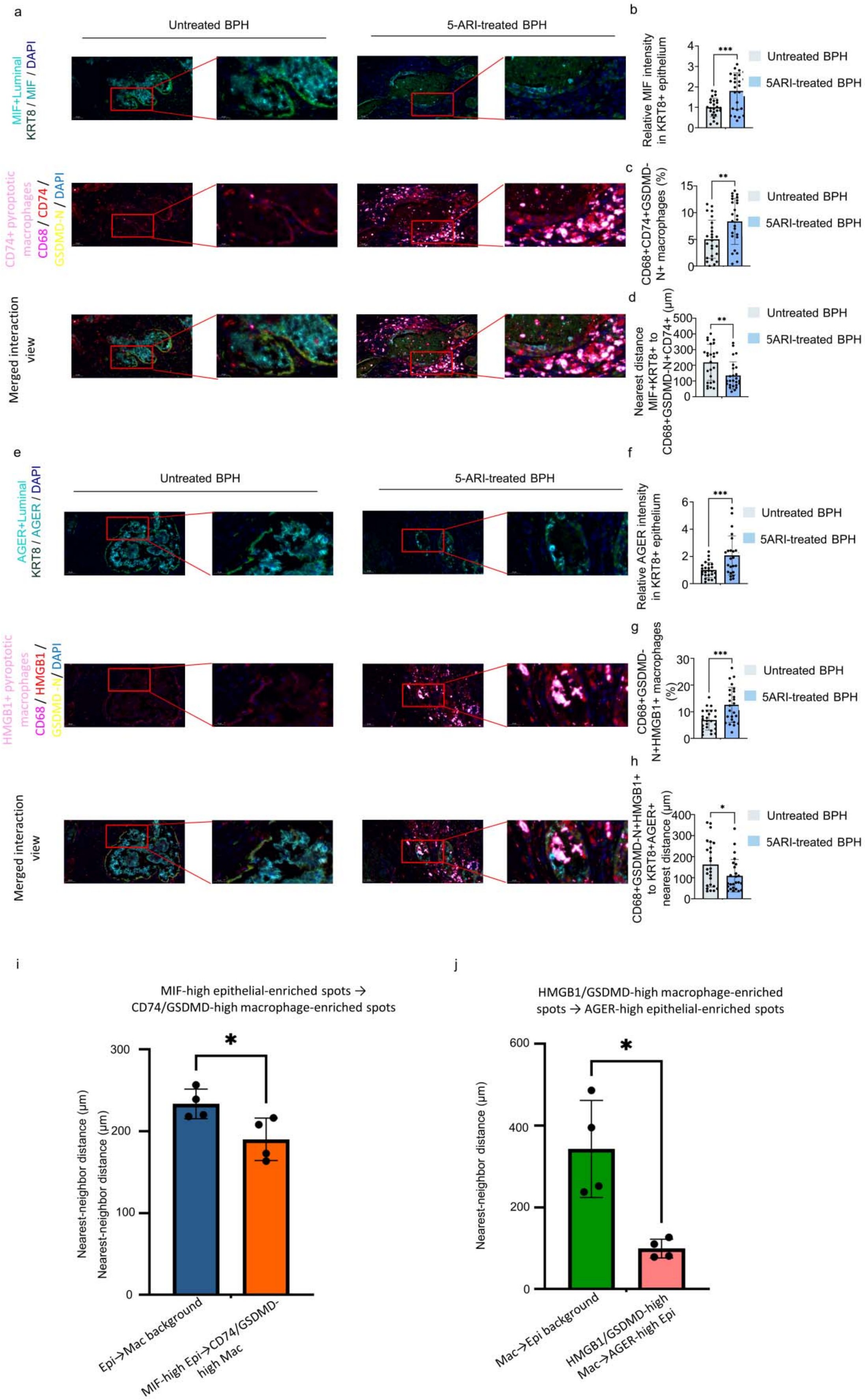
a
Untreated BPH
5-ARI-treated BPH
MIF+Luminal
KRT8 / MIF / DAPI
CD74+ pyroptotic macrophages
CD68 / CD74 / GSDMD-N / DAPI
Merged interaction view
b
Relative MIF intensity in KRT8+ epithelium
***
Untreated BPH
5ARI-treated BPH
c
CD68+CD74+GSDMD-N+ macrophages (%)
**
Untreated BPH
5ARI-treated BPH
d
Nearest distance MIF+KRT8+ to CD68+GSDMD-N+CD74+ (μm)
**
Untreated BPH
5ARI-treated BPH
e
Untreated BPH
5-ARI-treated BPH
AGER+Luminal
KRT8 / AGER / DAPI
HMGB1+ pyroptotic macrophages
CD68 / HMGB1 / GSDMD-N/ DAPI
Merged interaction view
f
Relative AGER intensity in KRT8+ epithelium
***
Untreated BPH
5ARI-treated BPH
g
CD68+GSDMD-N+HMGB1+ macrophages (%)
***
Untreated BPH
5ARI-treated BPH
h
CD68+GSDMD-N+HMGB1+ to KRT8+AGER+ nearest distance (μm)
*
Untreated BPH
5ARI-treated BPH
i
MIF-high epithelial-enriched spots →
CD74/GSDMD-high macrophage-enriched spots
Nearest-neighbor distance (μm)
Nearest-neighbor distance (μm)
*
Epi→Mac background
MIF-high Epi→CD74/GSDMD-high Mac
j
HMGB1/GSDMD-high macrophage-enriched
spots → AGER-high epithelial-enriched spots
Nearest-neighbor distance (μm)
*
Mac→Epi background
HMGB1/GSDMD-high Mac→AGER-high Epi

Supplementary Fig. 8 | Multiplex immunofluorescence and ST-based nearest-neighbor analyses support spatial organization of the MIF–CD74/GSDMD and HMGB1/GSDMD–AGER epithelial–macrophage axes.

a, Representative multiplex immunofluorescence images showing the MIF–CD74 axis in untreated BPH and 5-ARI-treated BPH tissues. Images show KRT8/MIF/DAPI, CD68/CD74/GSDMD-N/DAPI, and merged interaction views. Overview and magnified images are shown for each group.

b, Quantification of relative MIF fluorescence intensity in KRT8+ luminal epithelial regions.

c, Quantification of CD68+CD74+GSDMD-N+ macrophages, shown as the percentage of total CD68+ macrophages.

d, Nearest-neighbor distance from MIF+KRT8+ epithelial regions to CD68+CD74+GSDMD-N+ macrophages.

e, Representative multiplex immunofluorescence images showing the HMGB1–AGER axis in untreated BPH and 5-ARI-treated BPH tissues. Images show KRT8/AGER/DAPI, CD68/GSDMD-N/HMGB1/DAPI, and merged interaction views. Overview and magnified images are shown for each group.

f, Quantification of relative AGER fluorescence intensity in KRT8+ luminal epithelial regions.

g, Quantification of CD68+GSDMD-N+HMGB1+ macrophages, shown as the percentage of total CD68+ macrophages.

h, Nearest-neighbor distance from CD68+GSDMD-N+HMGB1+ macrophages to KRT8+AGER+ epithelial regions.

i, ST-based nearest-neighbor analysis of the MIF–CD74/GSDMD axis. Targeted distances were calculated from MIF-high epithelial-enriched spots to the nearest CD74/GSDMD-high macrophage-enriched spots and compared with the epithelial-to-macrophage background distance.

j, ST-based nearest-neighbor analysis of the HMGB1/GSDMD–AGER axis. Targeted distances were calculated from HMGB1/GSDMD-high macrophage-enriched spots to the nearest AGER-high epithelial-enriched spots and compared with the macrophage-to-epithelial background distance.

Dots represent sample-level or section-level values as appropriate. Bars show mean ± s.e.m. For multiplex immunofluorescence quantification, values were calculated from five non-overlapping gland-containing fields of view per patient sample and averaged to generate one patient-level value before statistical analysis. For ST nearest-neighbor analysis, distances were summarized at the section level to avoid spot-level pseudoreplication. P values were calculated using two-sided tests as described in Methods. Exact P values are provided in the Source Data file. Scale bars, 50 μm.

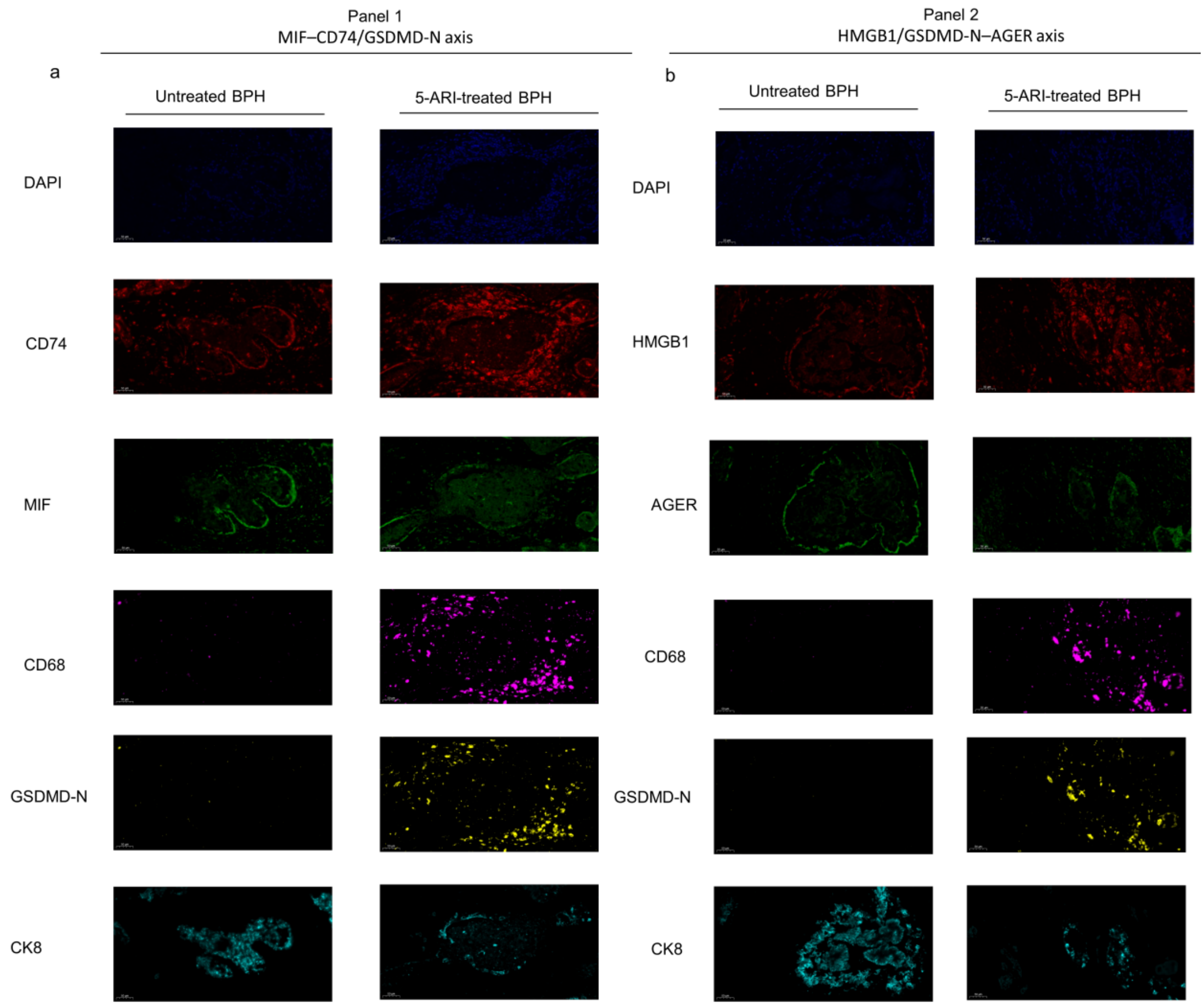

Supplementary Fig. 9 | Single-channel validation of multiplex immunofluorescence signals for the MIF–CD74/GSDMD-N and HMGB1/GSDMD-N–AGER axes.

a, Representative single-channel images corresponding to the MIF–CD74/GSDMD-N multiplex immunofluorescence panel shown in Supplementary Fig. 8a. Channels include DAPI, CD74, MIF, CD68, GSDMD-N, and KRT8.

b, Representative single-channel images corresponding to the HMGB1/GSDMD-N–AGER multiplex immunofluorescence panel shown in Supplementary Fig. 8e. Channels include DAPI, HMGB1, AGER, CD68, GSDMD-N, and KRT8.

Single-channel images are shown to verify marker-specific localization and channel separation. Scale bars, 50 μm.

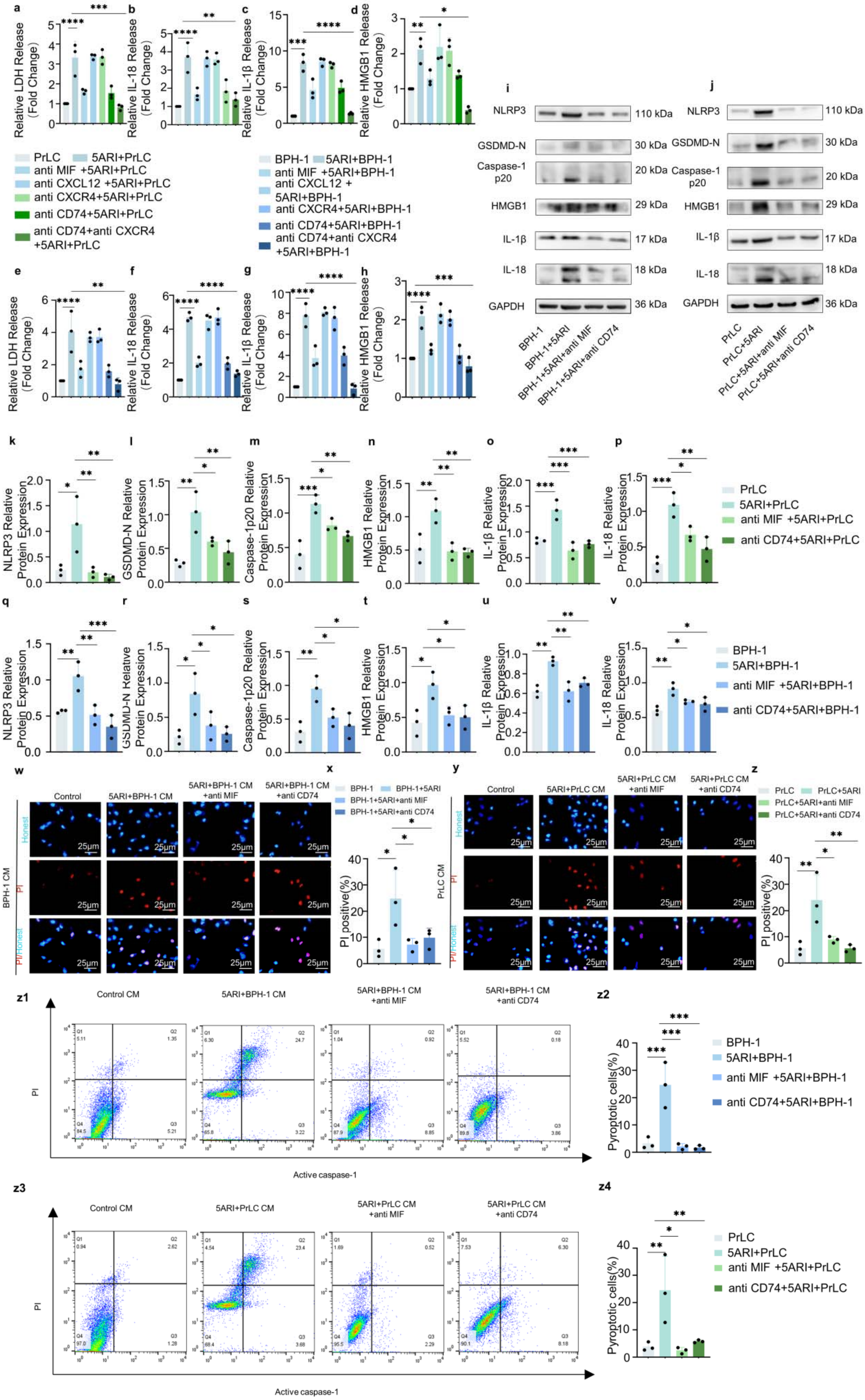

a
Relative LDH Release (Fold Change)
b
Relative IL-18 Release (Fold Change)
c
Relative IL-1β Release (Fold Change)
d
Relative HMGB1 Release (Fold Change)
PrLC
5ARI+PrLC
anti MIF +5ARI+PrLC
anti CXCL12 +5ARI+PrLC
anti CXCR4+5ARI+PrLC
anti CD74+5ARI+PrLC
anti CD74+anti CXCR4 +5ARI+PrLC
BPH-1
5ARI+BPH-1
anti MIF +5ARI+BPH-1
anti CXCL12 + 5ARI+BPH-1
anti CXCR4+5ARI+BPH-1
anti CD74+5ARI+BPH-1
anti CD74+anti CXCR4 +5ARI+BPH-1
e
Relative LDH Release (Fold Change)
f
Relative IL-18 Release (Fold Change)
g
Relative IL-1β Release (Fold Change)
h
Relative HMGB1 Release (Fold Change)
i
NLRP3
110 kDa
GSDMD-N
30 kDa
Caspase-1 p20
20 kDa
HMGB1
29 kDa
IL-1β
17 kDa
IL-18
18 kDa
GAPDH
36 kDa
BPH-1
BPH-1+5ARI
BPH-1+5ARI+anti MIF
BPH-1+5ARI+anti CD74
j
NLRP3
110 kDa
GSDMD-N
30 kDa
Caspase-1 p20
20 kDa
HMGB1
29 kDa
IL-1β
17 kDa
IL-18
18 kDa
GAPDH
36 kDa
PrLC
PrLC+5ARI
PrLC+5ARI+anti MIF
PrLC+5ARI+anti CD74
k
NLRP3 Relative Protein Expression
l
GSDMD-N Relative Protein Expression
m
Caspase-1p20 Relative Protein Expression
n
HMGB1 Relative Protein Expression
o
IL-1β Relative Protein Expression
p
IL-18 Relative Protein Expression
PrLC
5ARI+PrLC
anti MIF +5ARI+PrLC
anti CD74+5ARI+PrLC
q
NLRP3 Relative Protein Expression
r
GSDMD-N Relative Protein Expression
s
Caspase-1p20 Relative Protein Expression
t
HMGB1 Relative Protein Expression
u
IL-1β Relative Protein Expression
v
IL-18 Relative Protein Expression
BPH-1
5ARI+BPH-1
anti MIF +5ARI+BPH-1
anti CD74+5ARI+BPH-1
w
Control
5ARI+BPH-1 CM
5ARI+BPH-1 CM +anti MIF
5ARI+BPH-1 CM +anti CD74
BPH-1 CM
Honest
PI
PI/Honest
25μm
x
BPH-1
BPH-1+5ARI
BPH-1+5ARI+anti MIF
BPH-1+5ARI+anti CD74
PI positive(%)
y
Control
5ARI+PrLC CM
5ARI+PrLC CM +anti MIF
5ARI+PrLC CM +anti CD74
PrLC CM
Honest
PI
PI/Honest
25μm
z
PrLC
PrLC+5ARI
PrLC+5ARI+anti MIF
PrLC+5ARI+anti CD74
PI positive(%)
z1
Control CM
5ARI+BPH-1 CM
5ARI+BPH-1 CM +anti MIF
5ARI+BPH-1 CM +anti CD74
PI
Active caspase-1
z2
Pyroptotic cells(%)
BPH-1
5ARI+BPH-1
anti MIF +5ARI+BPH-1
anti CD74+5ARI+BPH-1
z3
Control CM
5ARI+PrLC CM
5ARI+PrLC CM +anti MIF
5ARI+PrLC CM +anti CD74
PI
Active caspase-1
z4
Pyroptotic cells(%)
PrLC
5ARI+PrLC
anti MIF +5ARI+PrLC
anti CD74+5ARI+PrLC

Supplementary Fig. 10 | 5-ARI–treated epithelial conditioned media induce macrophage pyroptosis/inflammatory release, attenuated by MIF–CD74 blockade

a–d, Soluble readouts with primary luminal epithelial conditioned media (PrLC-CM). Compared with control PrLC-CM, 5-ARI+PrLC-CM markedly increases LDH release (a), IL-18 release (b), IL-1β release (c), and HMGB1 release (d). Neutralizing MIF or blocking its receptor CD74 substantially reduces these readouts, whereas blocking CXCL12 or its receptor CXCR4 has minimal impact on these release metrics, as shown. All values are normalized to Ctrl-CM = 1.

e–h, Soluble readouts with BPH-1 epithelial conditioned media. Compared with control BPH-1-CM, 5-ARI+BPH-1-CM similarly increases LDH release (e), IL-18 release (f), IL-1β release (g), and HMGB1 release (h). Anti-MIF or anti-CD74 partially or robustly reverses these increases, whereas anti-CXCL12 or anti-CXCR4 has minimal effect on these pyroptosis-associated release readouts.

i–j, Representative western blots. Under 5-ARI-CM exposure, macrophages upregulate NLRP3, GSDMD-N, and cleaved caspase-1 p20, accompanied by increased HMGB1, IL-1β, and IL-18. These changes are attenuated by anti-MIF or anti-CD74. Panel i shows the BPH-1-CM condition, and panel j shows the PrLC-CM condition.

k–p, Quantified western blot data for the PrLC-CM condition: NLRP3 (k), GSDMD-N (l), cleaved caspase-1 p20 (m), HMGB1 (n), IL-1β (o), and IL-18 (p).

q–v, Quantified western blot data for the BPH-1-CM condition: NLRP3 (q), GSDMD-N (r), cleaved caspase-1 p20 (s), HMGB1 (t), IL-1β (u), and IL-18 (v).

w–y, Immunofluorescence analysis of macrophage pyroptosis. Representative images show Hoechst, PI, and merged PI/Hoechst staining after exposure to control CM, 5-

ARI-CM, 5-ARI-CM+anti-MIF, or 5-ARI-CM+anti-CD74. Panel w shows the BPH-1-CM condition, and panel y shows the PrLC-CM condition. Scale bars, 25 μm.

x, z, Quantification of PI-positive macrophages in the immunofluorescence assay. The percentage of PI-positive cells increases after 5-ARI-CM exposure and decreases after MIF neutralization or CD74 blockade. Panel x shows the BPH-1-CM condition, and panel z shows the PrLC-CM condition.

z1–z4, Flow-cytometric analysis of macrophage pyroptosis using active caspase-1 FAM-FLICA and PI staining. Representative gating plots are shown for the BPH-1-CM condition (z1) and the PrLC-CM condition (z3). Quantification of active caspase-1+PI+ pyroptotic macrophages is shown for the BPH-1-CM condition (z2) and the PrLC-CM condition (z4). 5-ARI-CM increases the pyroptotic macrophage fraction, whereas MIF neutralization or CD74 blockade reduces it toward baseline.

Statistics: Bars/dots show mean ± s.e.m.; n = 3 independent biological replicates unless otherwise indicated. Multi-group comparisons were performed using the panel-specific omnibus and multiplicity-adjusted post hoc procedures described in the Methods. Significance: ns, not significant; *P < 0.05; **P < 0.01; ***P < 0.001; ****P < 0.0001. Exact P values are provided in the Source Data file.

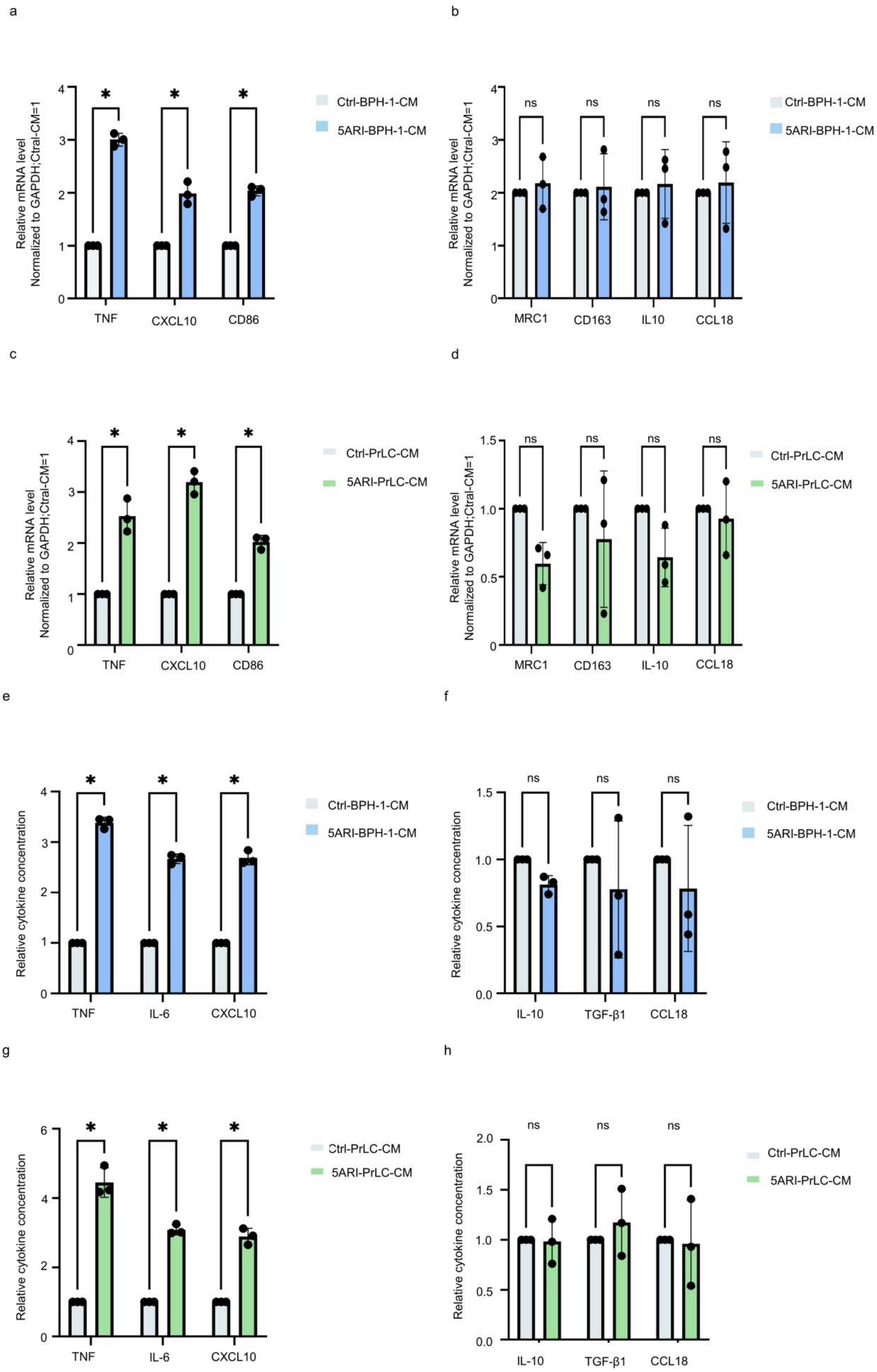

a
Relative mRNA level
Normalized to GAPDH;Ctral-CM=1
TNF
CXCL10
CD86
Ctrl-BPH-1-CM
5ARI-BPH-1-CM
b
Relative mRNA level
Normalized to GAPDH;Ctral-CM=1
MRC1
CD163
IL10
CCL18
ns
Ctrl-BPH-1-CM
5ARI-BPH-1-CM
c
Relative mRNA level
Normalized to GAPDH;Ctral-CM=1
TNF
CXCL10
CD86
Ctrl-PrLC-CM
5ARI-PrLC-CM
d
Relative mRNA level
Normalized to GAPDH;Ctral-CM=1
MRC1
CD163
IL-10
CCL18
ns
Ctrl-PrLC-CM
5ARI-PrLC-CM
e
Relative cytokine concentration
TNF
IL-6
CXCL10
Ctrl-BPH-1-CM
5ARI-BPH-1-CM
f
Relative cytokine concentration
IL-10
TGF-β1
CCL18
ns
Ctrl-BPH-1-CM
5ARI-BPH-1-CM
g
Relative cytokine concentration
TNF
IL-6
CXCL10
Ctrl-PrLC-CM
5ARI-PrLC-CM
h
Relative cytokine concentration
IL-10
TGF-β1
CCL18
ns
Ctrl-PrLC-CM
5ARI-PrLC-CM

Supplementary Fig. 11 | 5-ARI-conditioned epithelial secretomes induce M1-like inflammatory features without consistent induction of M2-like remodeling/regulatory markers.

a–b, qRT–PCR analysis of selected macrophage functional-state markers in hMDMs exposed to Ctrl-BPH-1-CM or 5-ARI-BPH-1-CM. a, M1-like inflammatory markers, including TNF, CXCL10, and CD86. b, M2-like remodeling/regulatory markers, including MRC1, CD163, IL10, and CCL18.

c–d, qRT–PCR analysis of selected macrophage functional-state markers in hMDMs exposed to Ctrl-PrLC-CM or 5-ARI-PrLC-CM. c, M1-like inflammatory markers, including TNF, CXCL10, and CD86. d, M2-like remodeling/regulatory markers, including MRC1, CD163, IL10, and CCL18.

e–f, ELISA-based relative cytokine profiling in hMDMs exposed to Ctrl-BPH-1-CM or 5-ARI-BPH-1-CM. e, Inflammatory cytokines/chemokines, including TNF-α, IL-6, and CXCL10. f, Remodeling/regulatory factors, including IL-10, TGF-β1, and CCL18.

g–h, ELISA-based relative cytokine profiling in hMDMs exposed to Ctrl-PrLC-CM or 5-ARI-PrLC-CM. g, Inflammatory cytokines/chemokines, including TNF-α, IL-6, and CXCL10. h, Remodeling/regulatory factors, including IL-10, TGF-β1, and CCL18.

For qRT–PCR, expression values were normalized to GAPDH and are shown as fold change relative to the matched Ctrl-CM group, which was set to 1. For ELISA, cytokine concentrations were normalized to the matched Ctrl-CM group and are shown as relative cytokine concentrations, with the matched Ctrl-CM group set to 1. Dots represent independent biological replicates, and bars show mean ± s.e.m.; $n = 3$ independent biological replicates. For each readout, normalized values in the 5-ARI-CM group were compared with the theoretical control value of 1 using a two-sided

one-sample t test. Exact P values are provided in the Source Data file.

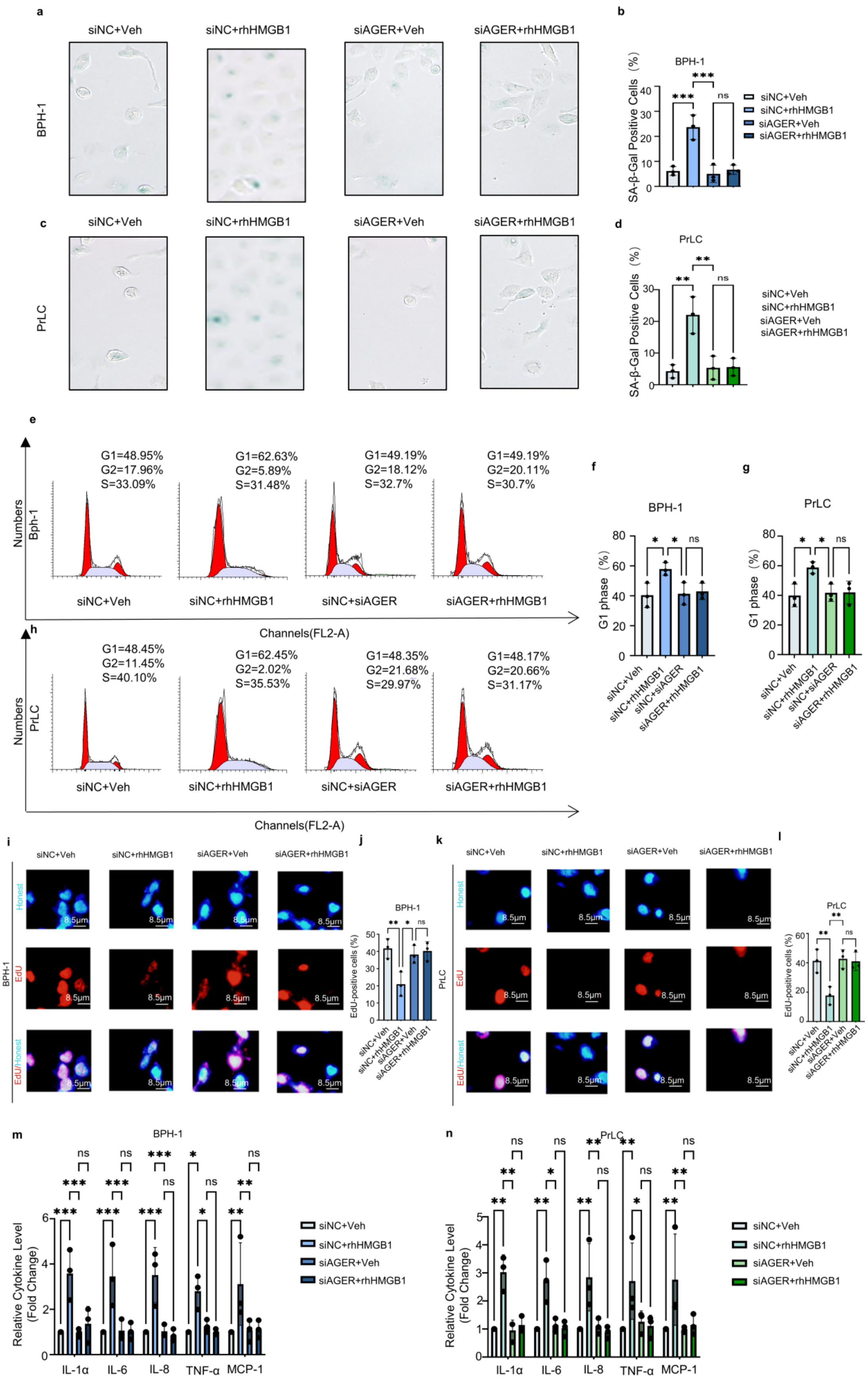

Supplementary Fig. 12 | Recombinant HMGB1 induces epithelial senescence and proliferation restraint in an AGER-dependent manner

a–d, Representative SA-β-gal staining and quantification in BPH-1 cells and primary PrLCs treated with vehicle or recombinant human HMGB1 (rhHMGB1) after transfection with negative-control siRNA (siNC) or AGER-targeting siRNA (siAGER).

e–h, Representative DNA-content flow-cytometry profiles and quantification of G1-phase fraction under the same conditions.

i–l, Representative EdU/Hoechst staining and quantification of EdU-positive cells.
m–n, Relative secreted protein levels of SASP-associated cytokines measured by ELISA, including IL-1α, IL-6, IL-8, TNF-α, and MCP-1, in BPH-1 cells and PrLCs. Data are shown as mean ± s.e.m.; n = 3 independent biological replicates per group. For each quantitative readout, group differences were analyzed using ordinary one-way ANOVA followed by Tukey's multiple-comparisons test unless otherwise indicated. For panel f, Šídák's multiple-comparisons test was used for the three prespecified pairwise comparisons shown in the panel. For the cytokine measurements in panels m and n, each cytokine was analyzed separately. ns, not significant; *P < 0.05; **P < 0.01; ***P < 0.001; ****P < 0.0001. Exact P values are provided in the Source Data file.

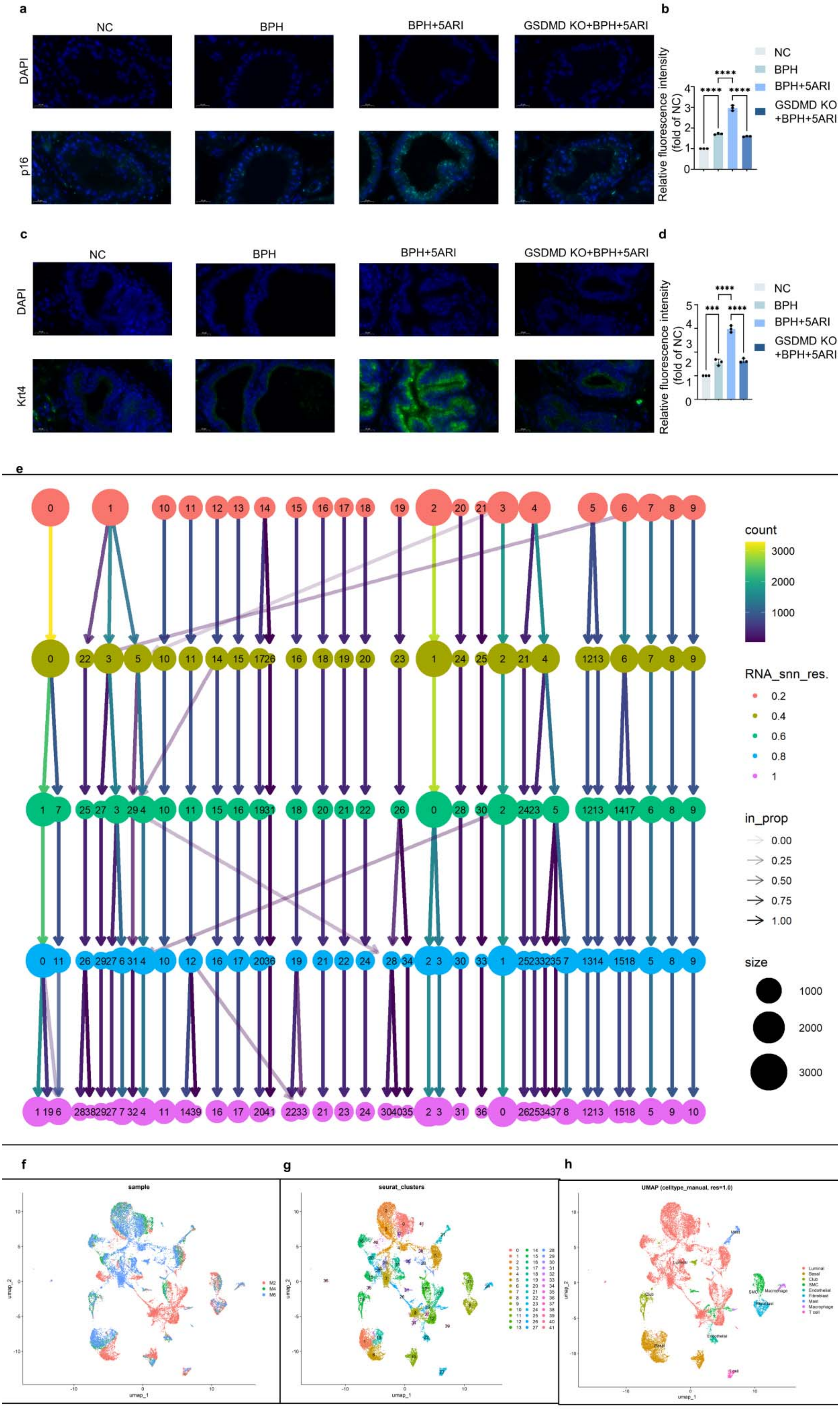

a
b
NC
BPH
BPH+5ARI
GSDMD KO+BPH+5ARI
DAPI
p16
Relative fluorescence intensity (fold of NC)
GSDMD KO +BPH+5ARI
c
d
Krt4
e
count
3000
2000
1000
RNA_snn_res.
0.2
0.4
0.6
0.8
1
in_prop
0.00
0.25
0.50
0.75
1.00
size
1000
2000
3000
f
g
h
sample
seurat_clusters
UMAP (celltype_manual, res=1.0)
umap_1
umap_2

Supplementary Fig. 13 | p16/Krt4 immunofluorescence in the Gsdmd mouse model and clustering stability of public Srd5a2-deficient prostate scRNA-seq data

a–b, Representative immunofluorescence images and quantification of p16 across the NC, BPH, BPH+5-ARI, and Gsdmd-KO+BPH+5-ARI groups. DAPI is shown in blue and p16 in green. Bars show relative fluorescence intensity normalized to the NC group; data are presented as mean ± s.e.m.; n = 3 independent animals per group. p16 expression was increased in the BPH+5-ARI group and reduced following Gsdmd loss. Scale bar, 15 μm.

c–d, Representative immunofluorescence images and quantification of the club-cell marker Krt4 across the same four groups. Bars show relative fluorescence intensity normalized to the NC group; data are presented as mean ± s.e.m.; n = 3 independent animals per group. Krt4 expression was increased in the BPH+5-ARI group and reduced in Gsdmd-deficient mice. Scale bar, 15 μm.
e, Clustree visualization of cluster relationships across the tested clustering resolutions, used to assess clustering stability and select the 42-cluster solution for downstream analysis.

f, UMAP projection of the integrated GSE235514 mouse prostate scRNA-seq dataset colored by sample identity. M2 represents the Srd5a2−/− sample, whereas M4 and M6 represent the Srd5a2+/− samples.

g, UMAP projection colored by Seurat cluster identity, showing the 42 clusters used for downstream annotation (clusters 0–41).

h, UMAP projection after manual annotation and consolidation into nine major cell classes—luminal, basal, club, smooth muscle, endothelial, fibroblast, mast, macrophage, and T cells—based on canonical-marker expression.

Statistics: For panels b and d, group differences were assessed using ordinary one-way ANOVA followed by Tukey's multiple-comparisons test. All tests were two-sided. Significance: ns, not significant; *P < 0.05; **P < 0.01; ***P < 0.001; ****P < 0.0001. Exact P values are provided in the Source Data file.

a

Club cells

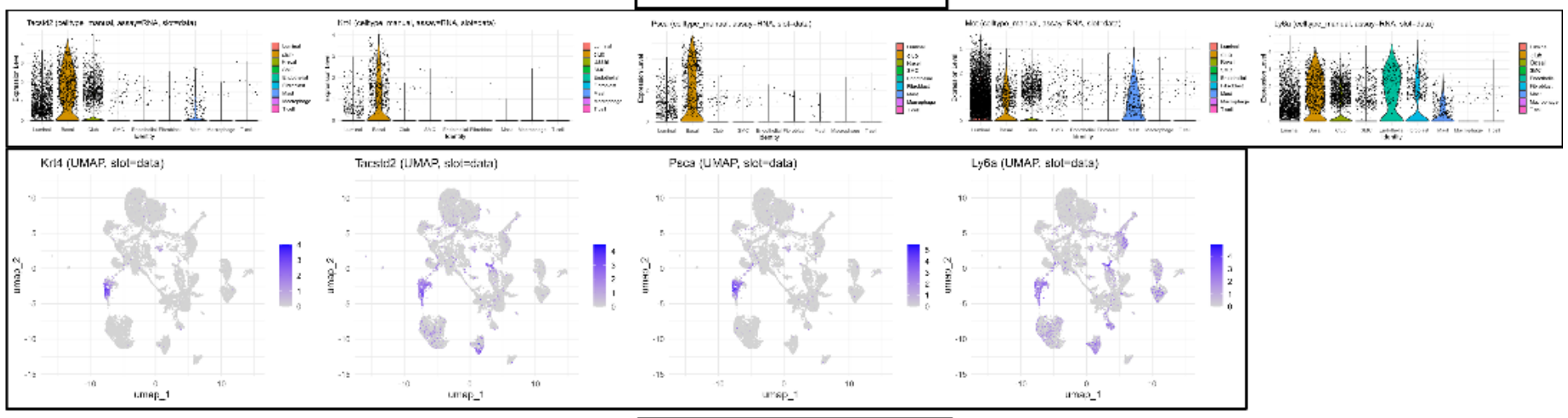

b

Basal cells

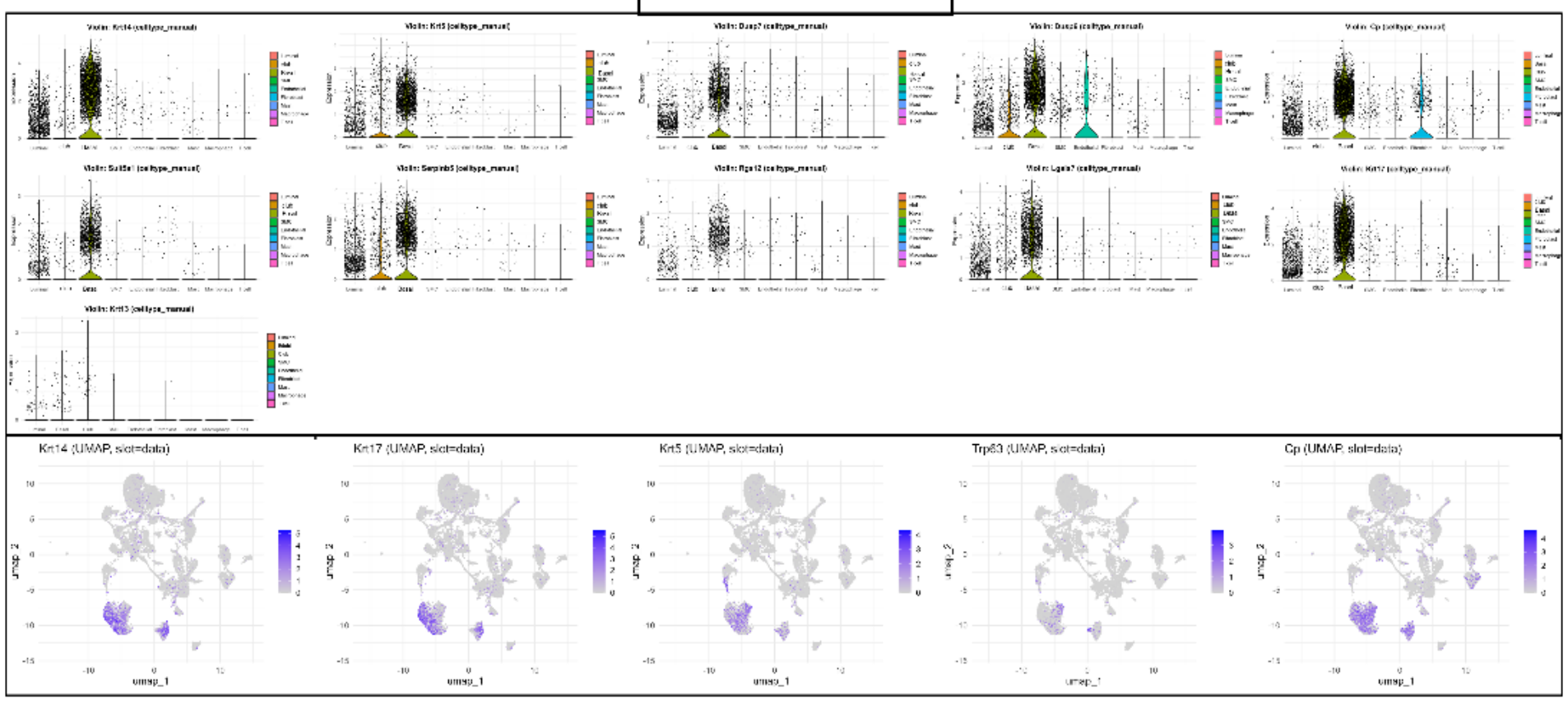

c

luminal cells

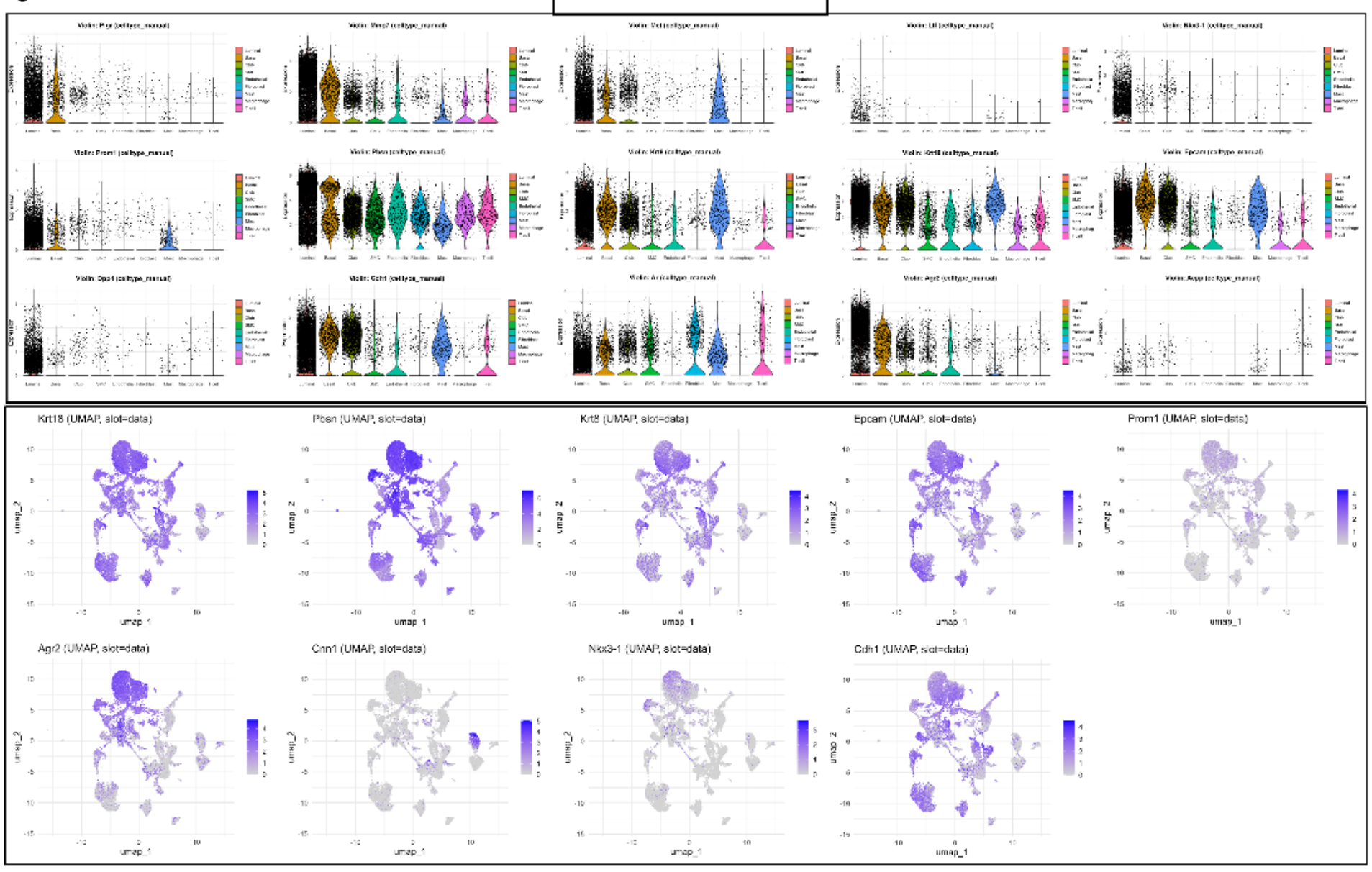

d

Endothelial cells

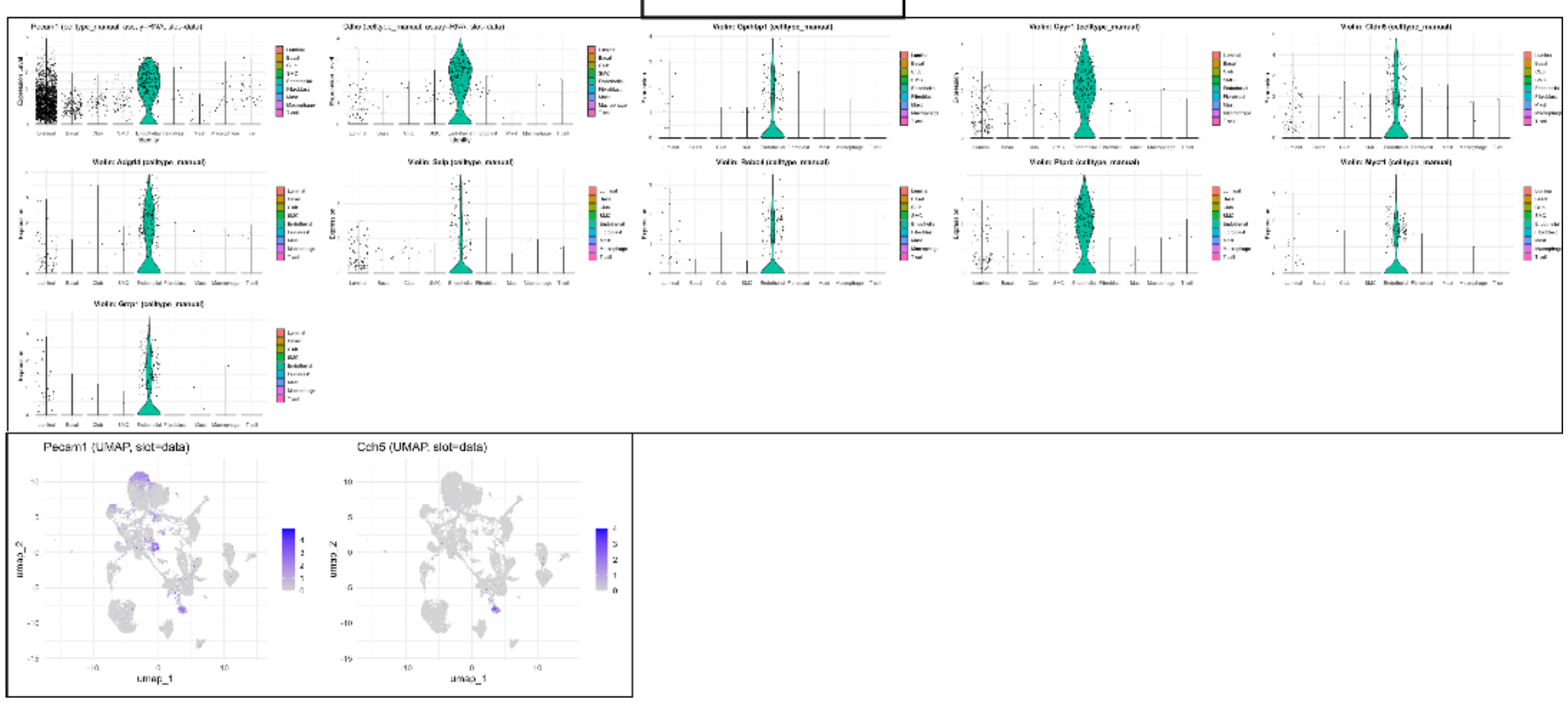

Supplementary Fig. 14 | Joint visualization of epithelial/endothelial lineage markers (violin plots + UMAP FeaturePlots)

a Club cells: Cp/Scgb1a1, Dusp6, Krt17 distribution across clusters and localization on UMAP (top: violins; bottom: FeaturePlots).

b Basal cells: Krt5, Krt14, Trp63 highly enriched in basal clusters with low background elsewhere.

c Luminal cells: Krt8, Krt18, Epcam and secretory/differentiation genes (e.g., Pbsn, Nkx3-1) are prominent in luminal clusters, largely exclusive of basal/club lineages.

d Endothelial cells: Pecam1, Cdh5 strongly expressed in endothelial clusters with low background in others.

Notes: Visualizations are based on the integrated GSE235514 dataset using the same UMAP embedding as Supplementary Fig. 13f–h. Violins depict within-cluster distributions/outliers; FeaturePlots display spatial enrichment. Illustrative only; no statistical testing performed.

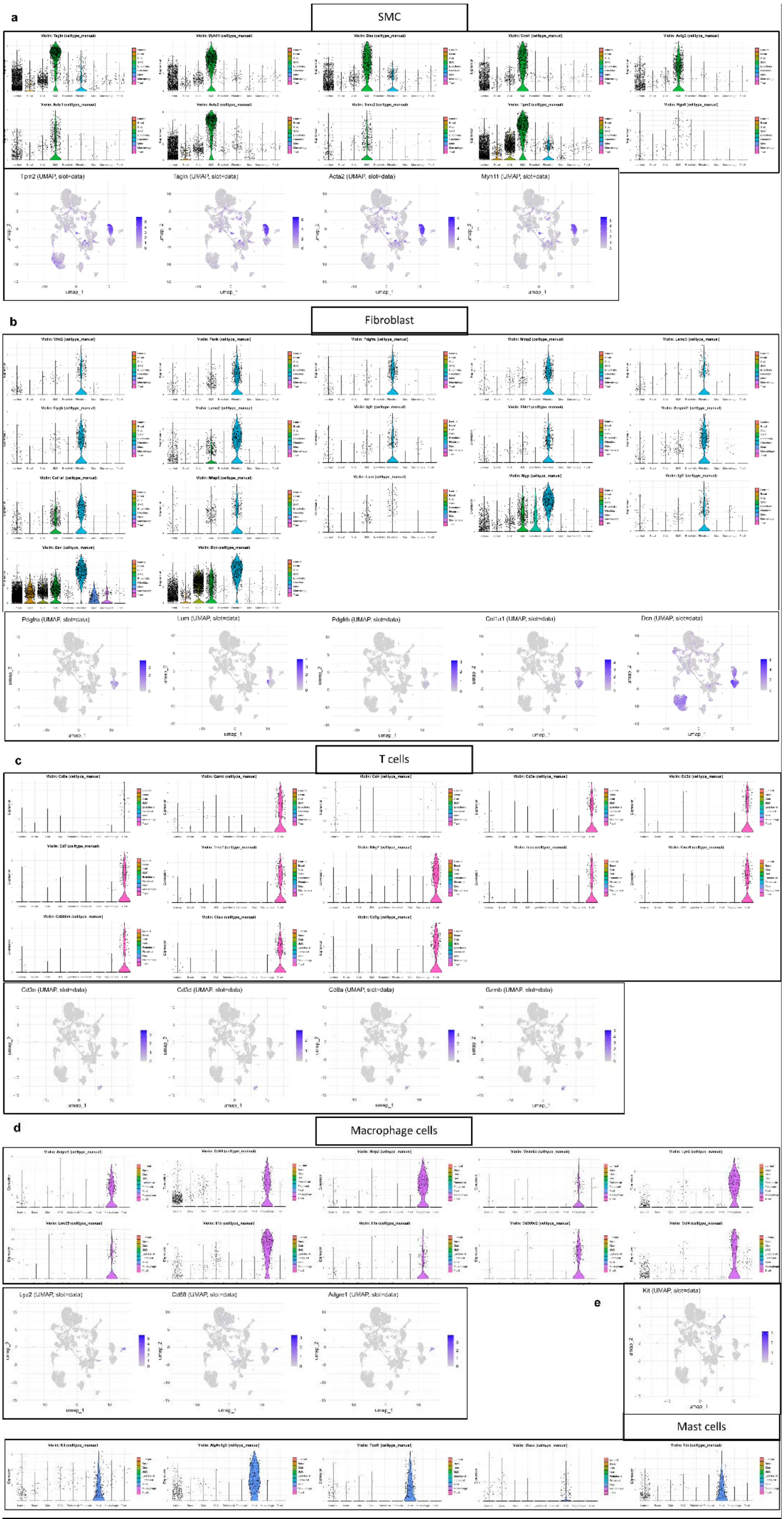
a
SMC
Tpm2 (UMAP, slot=data)
Tagln (UMAP, slot=data)
Acta2 (UMAP, slot=data)
Myh11 (UMAP, slot=data)
b
Fibroblast
Pdgfra (UMAP, slot=data)
Lum (UMAP, slot=data)
Pdgfrb (UMAP, slot=data)
Col1a1 (UMAP, slot=data)
Dcn (UMAP, slot=data)
c
T cells
Cd3e (UMAP, slot=data)
Cd3d (UMAP, slot=data)
Cd8a (UMAP, slot=data)
Gzmb (UMAP, slot=data)
d
Macrophage cells
Lyz2 (UMAP, slot=data)
Cd68 (UMAP, slot=data)
Adgre1 (UMAP, slot=data)
e
Kit (UMAP, slot=data)
Mast cells

Supplementary Fig. 15 | Joint visualization of stromal and immune lineage markers (violin plots + UMAP FeaturePlots)

a SMC (smooth muscle cells): Acta2, Myh11, Tagln, Tpm2 are highly expressed within SMC clusters, with low background elsewhere. Top: violin plots across clusters (x-axis: clusters; y-axis: log-normalized expression). Bottom: UMAP FeaturePlots (color scale from low to high expression).

b Fibroblasts: Col1a1, Dcn, Pdgfra/Pdgfrb and matrix-remodeling genes are enriched in fibroblast clusters, appearing as locally continuous regions on UMAP.

c T cells: Cd3e, Cd3d, Cd8a (with additional effector genes such as Gzmb indicated in-figure) show lineage-specific high expression in T-cell clusters and low background elsewhere.

d Macrophages: Adgre1 (F4/80), Cd68, Lyz2 are strongly enriched in macrophage clusters, forming discrete immune islands largely exclusive of epithelial/stromal clusters.

e Mast cells: Kit (and tryptase-related genes as labeled) is highly expressed in mast-cell clusters, which appear as small, compact groups on UMAP.

Notes: Visualizations are based on the integrated GSE235514 mouse prostate scRNA-seq dataset using the same UMAP embedding as Supplementary Fig. 13f–h. Violins depict within-cluster distributions/outliers; FeaturePlots illustrate spatial enrichment. Together with Supplementary Fig. 14, these panels establish the molecular specificity of stromal and immune lineages. Illustrative only; no statistical testing performed.

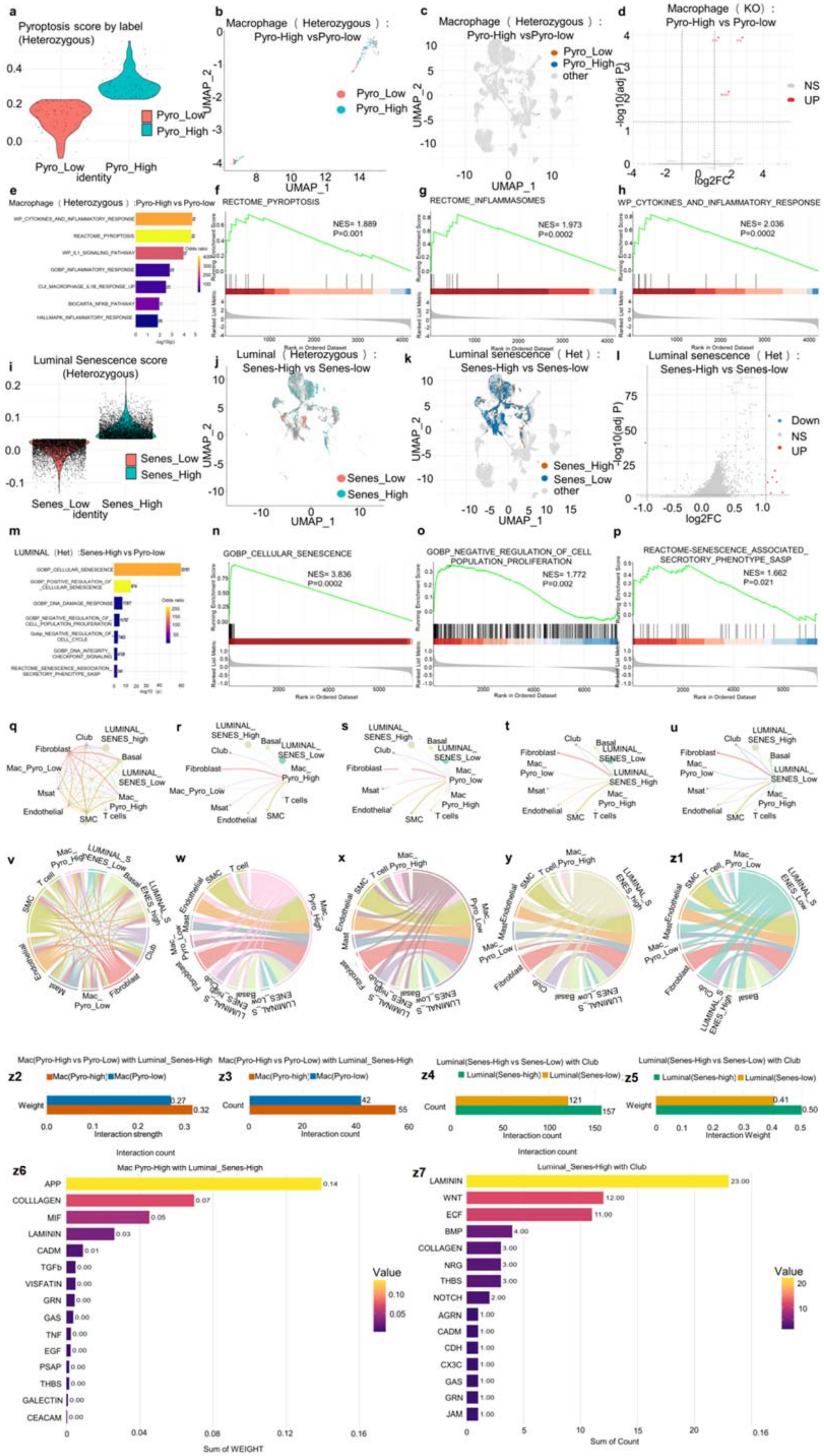

Pyroptosis score by label (Heterozygous)
Macrophage（Heterozygous）: Pyro-High vsPyro-low
Macrophage（KO）: Pyro-High vs Pyro-low
RECTOME_PYROPTOSIS
RECTOME_INFLAMMASOMES
WP_CYTOKINES_AND_INFLAMMATORY_RESPONSE
Luminal Senescence score (Heterozygous)
Luminal（Heterozygous）: Senes-High vs Senes-low
Luminal senescence（Het）: Senes-High vs Senes-low
GOBP_CELLULAR_SENESCENCE
GOBP_NEGATIVE_REGULATION_OF_CELL_POPULATION_PROLIFERATION
REACTOME-SENESCENCE_ASSOCIATED_SECRETORY_PHENOTYPE_SASP
Mac(Pyro-High vs Pyro-Low) with Luminal_Senes-High
Luminal(Senes-High vs Senes-Low) with Club
Mac Pyro-High with Luminal_Senes-High
Luminal_Senes-High with Club

Supplementary Fig. 16 | Cell–cell communication reveals selective coupling between "Pyro-High macrophages ↔ Senes-High luminal" and "Senes-High luminal ↔ club" (Srd5a2+/− cohort)

a–c Scores and distributions in the heterozygous cohort: violin plot of the pyroptosis score (a) and UMAP localization of Pyro-High/Pyro-Low macrophages (b,c).

d Volcano plot of DEGs: Pyro-High vs Pyro-Low macrophages.

e ORA bar plot highlighting inflammation/pyroptosis pathways.

f–h Macrophage GSEA: positive enrichment of REACTOME_PYROPTOSIS, REACTOME_INFLAMMASOMES, and WP_CYTOKINES_AND_INFLAMMATORY_RESPONSE.

i–k Luminal senescence scoring and UMAP localization: Senes-High vs Senes-Low.

l Volcano plot: luminal Senes-High vs Senes-Low.

m–p Luminal ORA/GSEA: enrichment for GOBP_CELLULAR_SENESCENCE, REACTOME_SASP, and GOBP_NEGATIVE_REGULATION_OF_CELL_POPULATION_PROLIFERATION.

q Whole-atlas interaction-count network view.

r–s Pyroptosis-stratified macrophage interaction-count networks: Pyro-High (r) versus Pyro-Low (s).

t–u Senescence-stratified luminal interaction-count networks: Senes-High (t) versus Senes-Low (u).

v–z1 Corresponding interaction-strength (weight) views for the networks shown in q–u: the whole atlas (v), Pyro-High macrophages (w), Pyro-Low macrophages (x), Senes-High luminal cells (y), and Senes-Low luminal cells (z1).

z2–z3 Pairwise contrasts showing greater Mac_Pyro-High ↔ Luminal_Senes-High communication than Mac_Pyro-Low ↔ Luminal_Senes-High communication in interaction strength (z2) and interaction count (z3).

z4–z5 Pairwise contrasts showing greater Luminal_Senes-High ↔ Club communication than Luminal_Senes-Low ↔ Club communication in interaction count (z4) and interaction strength (z5).

z6 Pathway ranking for “Mac_Pyro-High ↔ Luminal_Senes-High” communication (bidirectional, weight): MIF, including MIF–CD74 signaling, ranks among the leading pathways, together with APP, COLLAGEN, LAMININ, and other ECM/injury-related programs.

z7 Pathway ranking for “Luminal_Senes-High ↔ Club” communication (incoming, count): LAMININ, WNT, and EGF are the leading pathways, with a detectable NOTCH axis.

Statistics and thresholds: unless noted, tests are two-sided; ORA/GSEA report OR or ES, P, and FDR-q (cutoffs and multiple-testing control in Methods). Stratification used within-group medians of the respective scores. Units and color keys for network/chord diagrams are given in the figure legends.

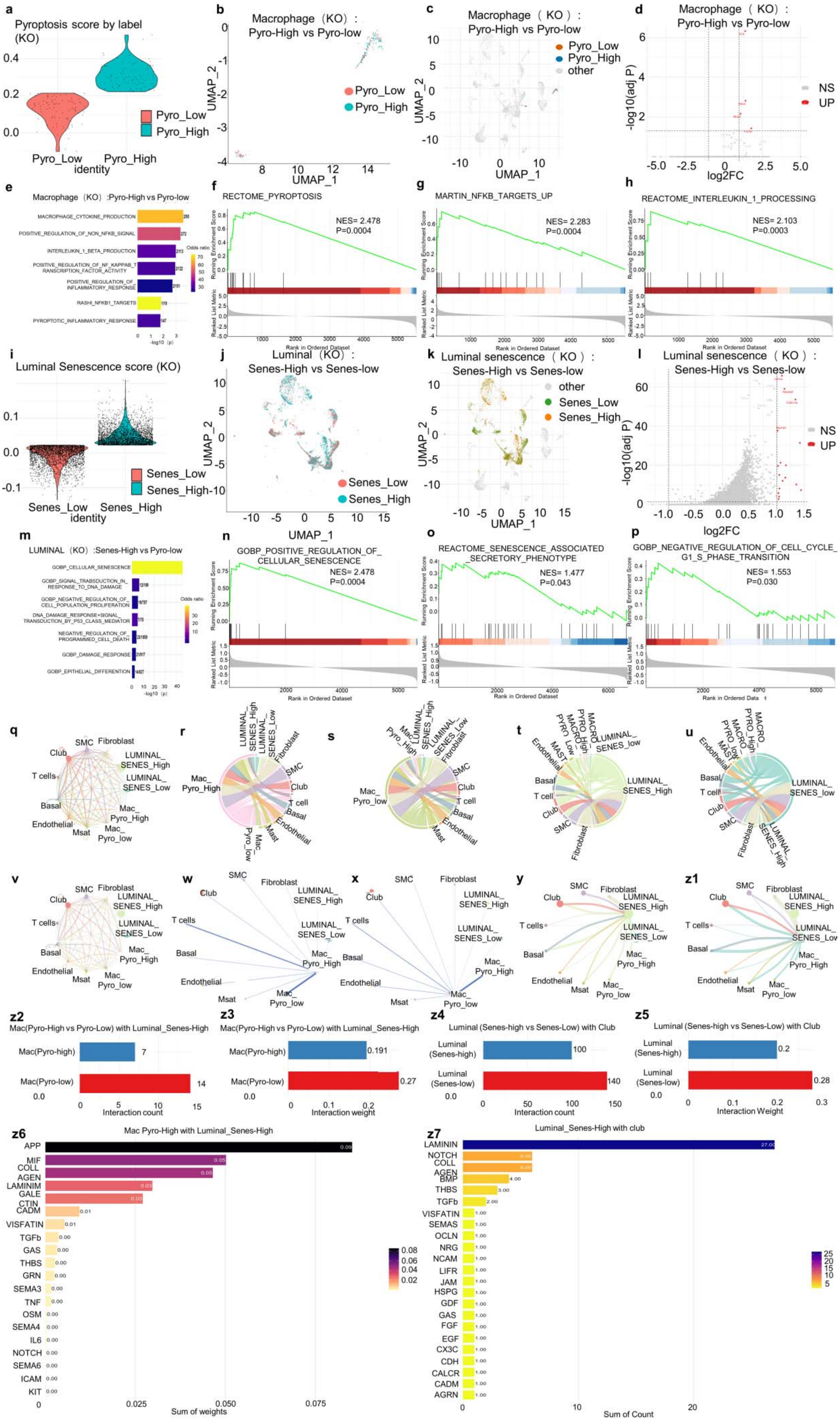

a
Pyroptosis score by label (KO)
Pyro_Low
Pyro_High
identity
b
Macrophage（KO）: Pyro-High vs Pyro-low
UMAP_1
UMAP_2
c
Macrophage（KO）: Pyro-High vs Pyro-low
other
d
Macrophage（KO）: Pyro-High vs Pyro-low
-log10(adj P)
log2FC
NS
UP
e
Macrophage（KO）:Pyro-High vs Pyro-low
MACROPHAGE_CYTOKINE_PRODUCTION
POSITIVE_REGULATION_OF_NON_NFKB_SIGNAL
INTERLEUKIN_1_BETA_PRODUCTION
POSITIVE_REGULATION_OF_NF_KAPPAB_TRANSCRIPTION_FACTOR_ACTIVITY
POSITIVE_REGULATION_OF_INFLAMMATORY_RESPONSE
RASHI_NFKB1_TARGETS
PYROPTOTIC_INFLAMMATORY_RESPONSE
Odds ratio
-log10（p）
f
RECTOME_PYROPTOSIS
NES= 2.478
P=0.0004
g
MARTIN_NFKB_TARGETS_UP
NES= 2.283
P=0.0004
h
REACTOME_INTERLEUKIN_1_PROCESSING
NES= 2.103
P=0.0003
Running Enrichment Score
Ranked List Metric
Rank in Ordered Dataset
i
Luminal Senescence score (KO)
Senes_Low
Senes_High
Senes_High-10
identity
j
Luminal（KO）: Senes-High vs Senes-low
k
Luminal senescence（KO）: Senes-High vs Senes-low
l
Luminal senescence（KO）: Senes-High vs Senes-low
m
LUMINAL（KO）:Senes-High vs Pyro-low
GOBP_CELLULAR_SENESCENCE
GOBP_SIGNAL_TRABSDUCTION_IN_RESPONSE_TO_DNA_DAMAGE
GOBP_NEGATIVE_REGULATION_OF_CELL_POPULATION_PROLIFERATION
DNA_DAMAGE_RESPONSE+SIGNAL_TRANSDUCTION_BY_P53_CLASS_MEDIATOR
NEGATIVE_REGULATION_OF_PROGRAMMED_CELL_DEATH
GOBP_DAMAGE_RESPONSE
GOBP_EPITHELIAL_DIFFERENTION
n
GOBP_POSITIVE_REGULATION_OF_CELLULAR_SENESCENCE
NES= 2.478
P=0.0004
o
REACTOME_SENESCENCE_ASSOCIATED_SECRETORY_PHENOTYPE
NES= 1.477
P=0.043
p
GOBP_NEGATIVE_REGULATION_OF_CELL_CYCLE_G1_S_PHASE_TRANSITION
NES= 1.553
P=0.030
q
r
s
t
u
v
w
x
y
z1
SMC
Fibroblast
Club
T cells
Basal
Endothelial
Msat
Mast
LUMINAL_SENES_High
LUMINAL_SENES_Low
Mac_Pyro_High
Mac_Pyro_low
z2
Mac(Pyro-High vs Pyro-Low) with Luminal_Senes-High
Mac(Pyro-high)
Mac(Pyro-low)
Interaction count
z3
Mac(Pyro-High vs Pyro-Low) with Luminal_Senes-High
Interaction weight
z4
Luminal (Senes-high vs Senes-Low) with Club
Luminal (Senes-high)
Luminal (Senes-low)
Interaction count
z5
Luminal (Senes-high vs Senes-Low) with Club
Interaction Weight
z6
Mac Pyro-High with Luminal_Senes-High
APP
MIF
COLL
AGEN
LAMININ
GALE
CTIN
CADM
VISFATIN
TGFb
GAS
THBS
GRN
SEMA3
TNF
OSM
SEMA4
IL6
NOTCH
SEMA6
ICAM
KIT
Sum of weights
z7
Luminal_Senes-High with club
LAMININ
NOTCH
COLL
AGEN
BMP
THBS
TGFb
VISFATIN
SEMAS
OCLN
NRG
NCAM
LIFR
JAM
HSPG
GDF
GAS
FGF
EGF
CX3C
CDH
CALCR
CADM
AGRN
Sum of Count

Supplementary Fig. 17 | Ligand–receptor communication reveals selective coupling between “Pyro-High macrophages ↔ Senes-High luminal” and “Senes-High luminal ↔ club” (Srd5a2−/− cohort)

a–c UMAP distributions of Pyro-High/Low macrophages and Senes-High/Low luminal cells (stratified by within-group medians).

d Volcano plot of differential expression: Pyro-High vs Pyro-Low.

e ORA bar plot showing significant enrichment of inflammatory/pyroptosis pathways.

f–h GSEA: positive enrichment of REACTOME_PYROPTOSIS, REACTOME_INFLAMMASOMES, and WP_CYTOKINES_AND_INFLAMMATORY_RESPONSE.

i–k UMAP distributions for Senes-High/Low; **l** corresponding volcano plot.

m–p ORA/GSEA: significant enrichment of GOBP_CELLULAR_SENESCENCE, REACTOME_SASP, GOBP_NEGATIVE_REGULATION_OF_CELL_POPULATION_PROLIFERATION, and DNA-damage/cell-cycle checkpoint programs.

q Whole-atlas interaction-count (count) network.

r–s Macrophage networks stratified by pyroptosis score: Pyro-High (**r**) vs Pyro-Low (**s**).

t–u Luminal networks stratified by senescence score: Senes-High (**t**) vs Senes-Low (**u**).

v–z1 Matching chord/arc diagrams for interaction strength (weight) corresponding to **r–u**.

z2–z3 Pairwise comparisons: Mac_Pyro-High ↔ Luminal_Senes-High exceeds Mac_Pyro-Low ↔ Luminal_Senes-High in interaction count (**z2**) and strength (**z3**).

z4–z5 Pairwise comparisons: Luminal_Senes-High ↔ Club exceeds Luminal_Senes-Low ↔ Club in interaction count (**z4**) and strength (**z5**).

z6 Pathway ranking (bidirectional, weight) for “Mac_Pyro-High ↔ Luminal_Senes-High”: MIF (MIF–CD74) among top signals, alongside APP/COLLAGEN/LAMININ ECM/injury programs.

z7 Pathway ranking (incoming, count) for “Luminal_Senes-High ↔ Club”:

LAMININ, WNT, and EGF dominate, with enrichment of the NOTCH axis.**Statistics and thresholds:** Unless otherwise indicated, tests are two-sided. ORA/GSEA report OR/ES, P, and FDR-q (thresholds and multiple-testing control in Methods). Stratification thresholds are the within-group medians of the respective scores.
